# Domino Grignard Addition/Cope–House Reaction for the Synthesis of Polyhydroxylated 3‑Methylindolizidines Analogous to Castanospermine

**DOI:** 10.1021/acs.orglett.5c03187

**Published:** 2025-09-02

**Authors:** Thomas Lulli, Filippo Dei, Macarena Martínez-Bailén, Camilla Matassini, Cristina Faggi, Francesca Cardona, Andrea Goti

**Affiliations:** Dipartimento di Chimica ‘Ugo Schiff’ (DICUS), 9300 Università di Firenze, via della Lastruccia 3-13, 50019 Sesto Fiorentino (FI), Italy

## Abstract

The first synthesis of polyhydroxylated 3-methylindolizidines is reported. A straightforward domino butenylmagnesium bromide addition/Cope-House reaction to carbohydrate-derived nitrones afforded efficiently the methyl pyrrolidine moiety with good stereoselectivity, which can be reversed by use of Lewis acids. A subsequent one-pot deprotection/deoxygenation/reductive amination step furnished the desired bicyclic architecture, allowing us to afford the desired final products in 3–4 steps from the starting nitrones and 18–30% overall yields.

## Graphical abstract

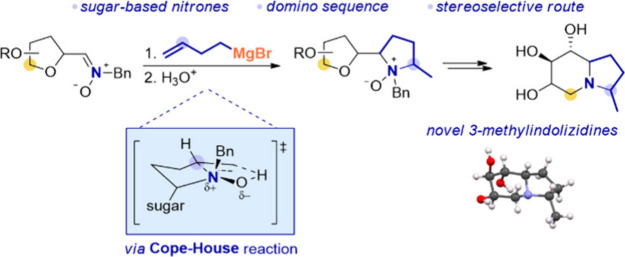

Castanospermine **1** (Figure 1) is a well-known indolizidine iminosugar, first isolated in 1981 from the seeds of *Castanospermum australe* and known to be poisonous for animals, especially cattle and horses.^1^ It exhibits strong α- and β-glucosidase inhibitory properties, affecting also the glycogen catabolism in the lysosomes.^2^ From the same species, several analogs (**2**–**5**, Figure 1) were isolated^3–7^ which also showed α-glucosidase inhibition (data are absent for 6,8-di-*epi*-castanospermine **5**) to a greater or lesser extent, with the most effective being castanospermine itself (*K*_i_ = 8 μM).^2,8^ These congeners were also found to inhibit other glycosidases.^5,7^ Castanospermine **1** displays a broad spectrum of biological effects (plant growth inhibition,^9^ feeding deterrence,^10^ insecticidal,^11^ and antiparasitic activity^12^ and antimetastatic and anticancer activities^13^) but is mainly known for its antiviral activity.^14^

**Figure 1:**
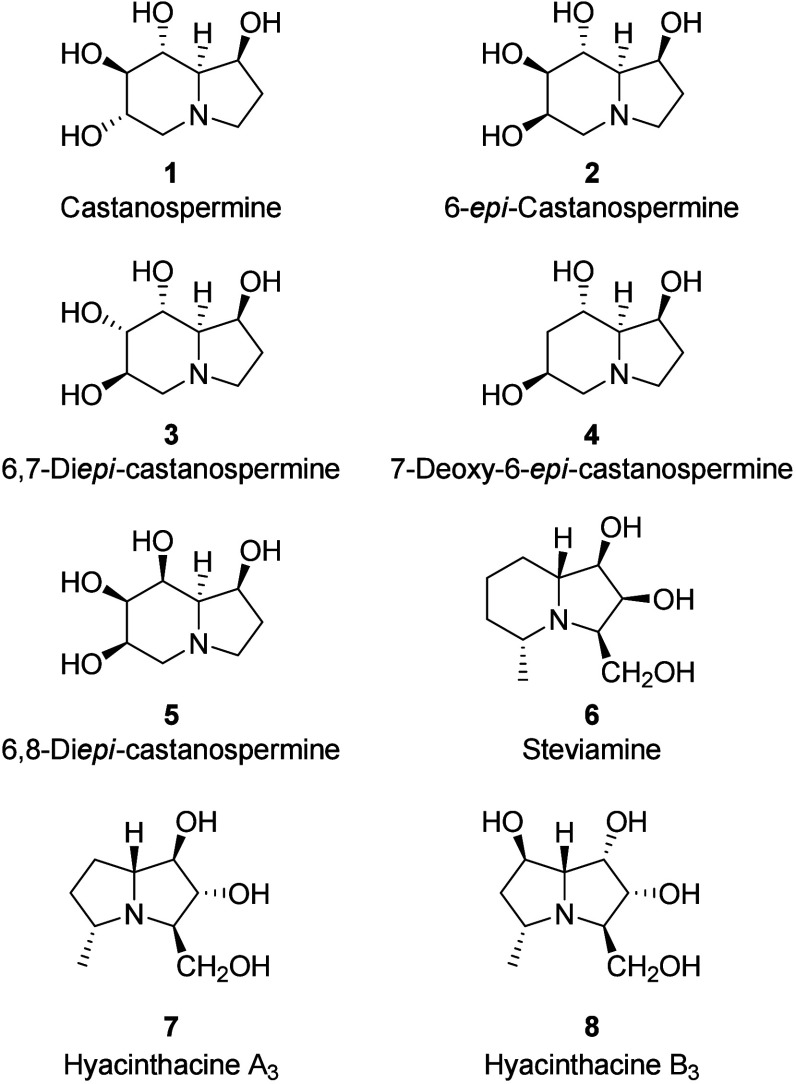
Examples of biologically active polyhydroxylated indolizidines **1**–**6** and pyrrolizidines **7** and **8**.

Many other natural and unnatural polyhydroxylated indolizidines are known for their biological activity and pharmaceutical potentialities.^15^ Even though extensive synthetic efforts have been made to access these compounds, the subject continues to attract much interest, in particular to tackle the obtainment of novel castanospermine analogs.^16^ Very few examples of polyhydroxylated indolizidines with an alkyl substituent adjacent to the bridgehead nitrogen atom have been reported so far. The more recently discovered steviamine **6** has been isolated from *Stevia rebaudiana* and *Veltheimia capensis* ^17^ and is the only natural indolizidine with a methyl substituent at C-5 in the piperidine ring (and hydroxymethylene group at C-3 in the pyrrolidine one). Besides this unique structural feature, steviamine did not show interesting glycosidase inhibitory activity, like any of the related derivatives that have been later synthesized.^18^

Apart from these few examples of indolizidine α-methyl-substituted at the piperidine ring, the synthesis and biological activity of polyhydroxylated indolizidines bearing a methyl substituent at the other position adjacent to the nitrogen atom, i.e., in the pyrrolidine moiety, remain unknown.

Conversely, polyhydroxylated pyrrolizidines—which may be considered ring-contracted forms of indolizidines^1b,19^ —with a methyl group adjacent to the nitrogen atom are diffuse in Nature, e.g, hyacinthacines A_3_ (**7**) and B_3_ (**8**), which are known to inhibit rat intestinal lactase and to display antidiabetic activity.^20^ This prompted us to envision a synthetic method to access polyhydroxylated 3-methylindolizidines, a new family of castanospermine analogs that eventually may display interesting biological features.

Most of the syntheses of hyacinthacine alkaloids and unnatural congeners involve intramolecular nucleophilic substitution,^21a–21e^ reductive amination on the ketone functionality,^21a–21c,21f–21h^ or, rarely, Bruylants alkylation reaction^21i,21j^ for the construction of the methyl pyrrolidine moiety. These strategies generally allow the control of the stereochemistry of the newly formed stereogenic centers to a certain degree but need a series of protection/deprotection steps that are step, time, and chemical wasting. Instead, for our purposes, we envisaged that the Cope–House (or reverse-Cope) reaction^22^ (Figure 2a) could be an attractive alternative to building the five-membered ring in a single step, overcoming these drawbacks.

**Figure 2:**
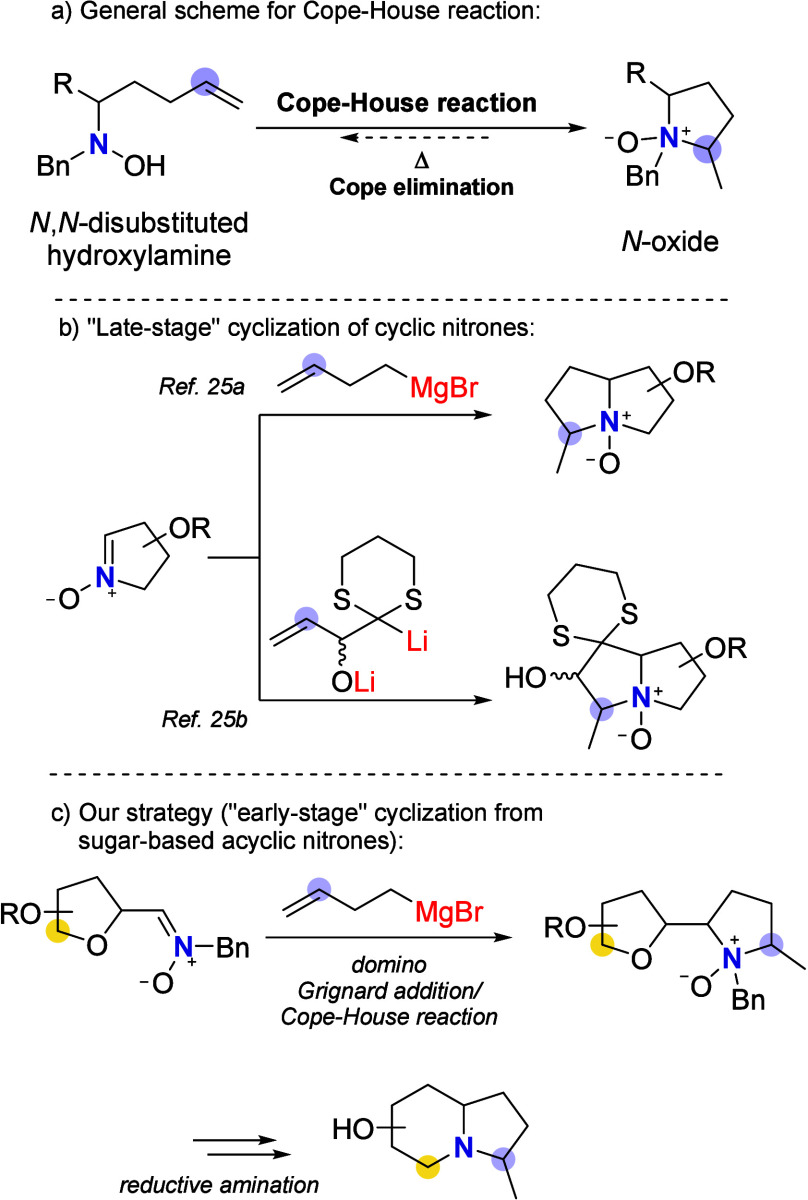
a) Schematic illustration of the Cope–House reaction. b) Previously reported use of the Cope–House reaction for the synthesis of bicyclic pyrrolizidine iminosugars. c) Our proposed strategy for the synthesis of polyhydroxylated 3-methylindolizidine.

Although this reaction has been known for several years, its use in the synthesis of complex compounds remains underexplored, mainly because the required unsaturated hydroxylamines are not easy to prepare.^22^ Nonetheless, a few remarkable results were achieved in the synthesis of elaborated molecules.^23^

Jäger and co-workers have used the Cope–House reaction as a key step for obtaining simple monocyclic iminosugars.^24^ Only two examples for the synthesis of bicyclic iminosugars with a pyrrolizidine nucleus have been reported, both based on the addition of a suitable organometallic reagent to preconstructed sugar-based cyclic nitrones for a “late-stage” formation of the methylpyrrolidine ring (Figure 2b).^25^ For our purposes, we envisaged that an “early-stage” Cope–House reaction of a suitable hydroxylamine generated by the addition to an acyclic carbohydrate-derived nitrone, followed by reduction/debenzylation/reductive amination under hydrogenolytic conditions,^26,27^ would afford the desired indolizidine moiety in two simple steps (Figure 2c). High versatility of this strategy would be ensured by the structural diversity of inexpensive carbohydrates available.

The appropriate unsaturated hydroxylamine precursors able to undergo a Cope–House cyclization were generated by the addition of butenyl nucleophiles to hexose-derived nitrones. The addition of 3-butenylmagnesium bromide to nitrone **9**, obtained on a gram scale from d-mannose,^27^ in THF at −78 °C was studied first (Scheme 1). TLC control after quenching of the reaction mixture attested the formation of highly polar compounds, either after quenching overnight or after 10 min of stirring, suggesting a rapid cyclization to *N*-oxide species. Surprisingly, no other manipulation (i.e., stirring the crude reaction mixture in CHCl_3_ or other solvents, which proved to be beneficial in previous cases^22^) was required to favor the Cope–House reaction, which occurred very rapidly after quenching in the mixture of THF and ammonium chloride solution. A complex mixture of diastereoisomers was obtained, as evidenced by the ^1^H NMR spectrum of the crude reaction mixture, from which no information about the diastereomeric ratio could be achieved. It is worth stressing that in this domino addition/cyclization three new stereocenters are formed. Therefore, each of the two possible *N*-hydroxylamines formed by addition to the nitrone diastereofaces may give two possible cyclization products—assuming that the Cope–House reaction occurs via the usual concerted mechanism, that no degradation of the hydroxylamine steps occurs (e.g., oxidation to the corresponding nitrone^27^), and that the cyclization is highly favored—for a total of at least four possible products.

**Scheme 1:**
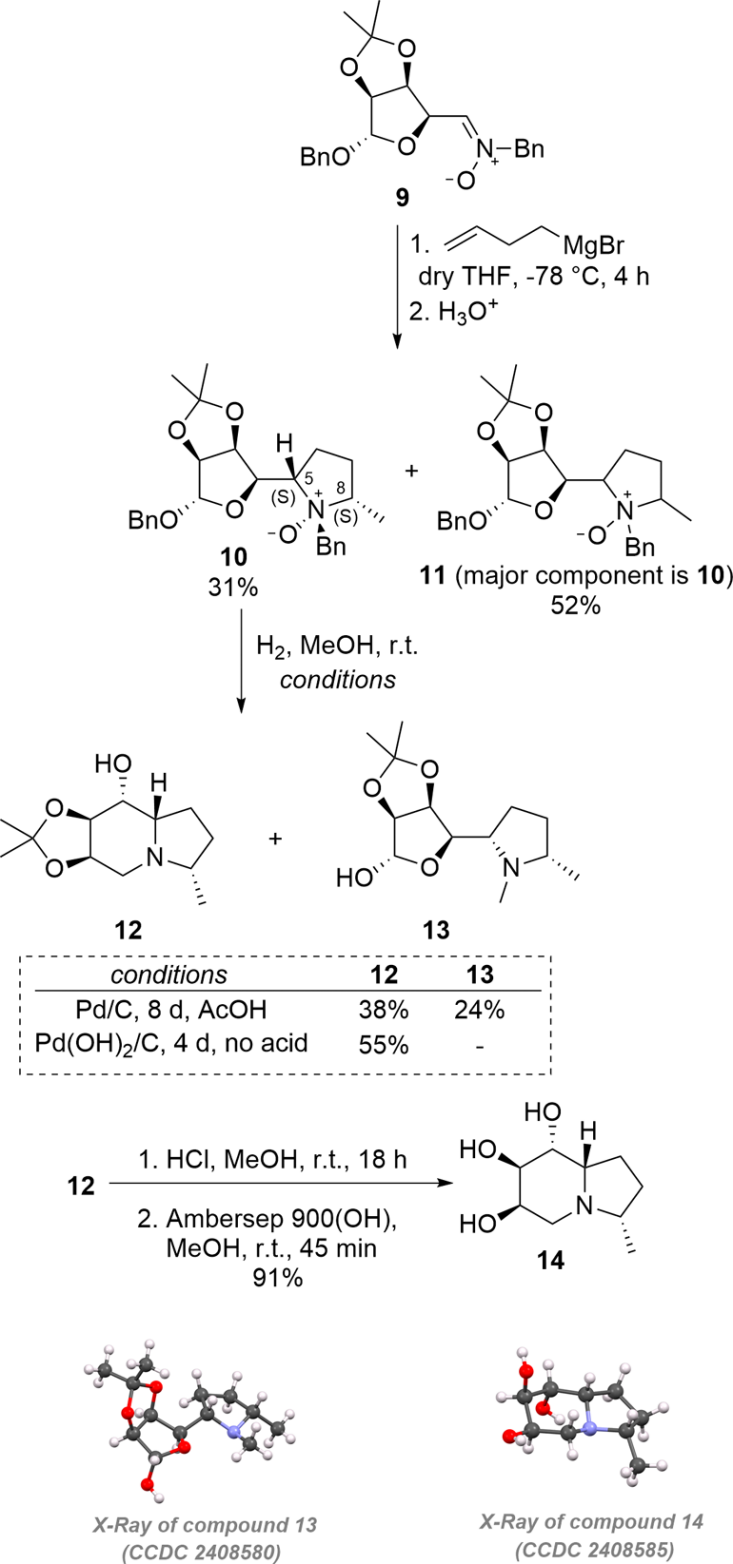
Synthesis of d-Mannose-Derived Polyhydroxylated 3-Methylindolizidine **14**

However, column chromatography allowed the isolation of pure *N*-oxide **10**—whose configuration at the newly formed stereocenters was assessed by analysis of the 1D-NOESY spectra—along with a complex mixture (52%) of the same product **10** as the major component and, most likely, the *N*-oxide(s) **11** arising from the cyclization of the hydroxylamine with the opposite configuration at C-5 (Scheme 1). Although the high complexity of the reaction mixture did not allow for the precise quantification of the diastereomeric ratio of the addition, it appears highly stereoselective, as inferred by the obtainment of diastereoisomers only in traces. The (*S*) absolute configuration at C-5 resulted from a preferred attack to the *Si* face of nitrone **9** as previously observed with other Grignard reagents,^27^ while the absolute configuration at C-8 can be explained considering a favored antiperiplanar arrangement between the bulky sugar moiety and the benzyl at nitrogen (Figure 3).^22^ The *trans* disposition of the methyl and benzyl groups is consistent with the stereospecificity of the pericyclic rearrangement. The reductive amination step to build the protected indolizidine **12** was first attempted using Pd/C as catalyst in the presence of acetic acid, which required a long reaction time and gave **12** in low yield because of the formation of the *N*-methyl byproduct **13** in the acidic methanolic solution. The structure of **13** was undoubtedly established by X-ray crystallographic analysis, which confirmed the stereochemical outcome of both the Grignard addition and the subsequent Cope–House reaction. Pd­(OH)_2_ proved to be a better catalyst for the cyclization step, giving a satisfactory yield of **12** for five individual steps (90% average yield), four of which are hydrogenations with no byproduct formation. The coupling of these two cascade processes is remarkable, consisting in the most concise approach to a polyhydroxy­indolizidine skeleton.

**Figure 3:**
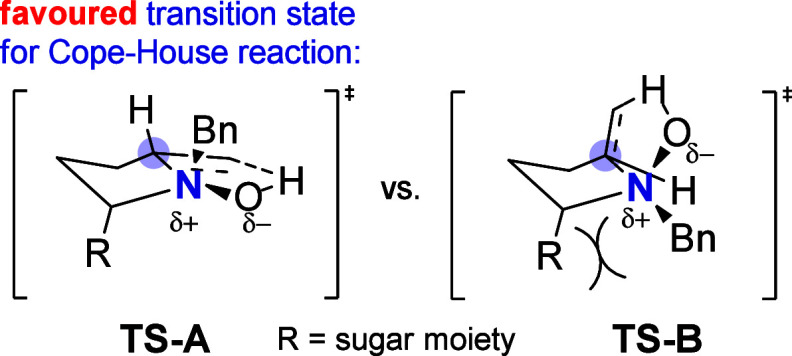
Favored transition state **TS-A** for the Cope–House reaction (left) and unfavored **TS-B** (right) due to the steric repulsion between the sugar moiety (R) and the benzyl group on the nitrogen atom.

Acidic deprotection of the acetonide functionality, followed by basic treatment, gave the final 3-methylindolizidine **14**, whose structure was confirmed by X-ray analysis (Scheme 1).

The possibility of inverting the absolute configuration at C-8a in the final indolizidine—that is, C-5 in the *N*-oxide—and investigating the subsequent Cope–House reaction was explored by using Lewis acids as additives in the addition of 3-butenylmagnesium bromide^27^ (Scheme 2). Et_2_AlCl and BF_3_·Et_2_O were chosen, with the latter giving the best results, as we previously observed with other Grignard reagents.^27^ Unfortunately, no information on diastereoselectivity could be drawn from the complex ^1^H NMR spectra of crude reaction mixtures, and the expected inversion of the configuration was then investigated after the next steps. A complex mixture of diastereomeric *N*-oxides **11** was obtained, with **10** as the minor one. All attempts to isolate the products at this stage or assign signals in the ^1^H NMR spectrum were unsuccessful.

**Scheme 2:**
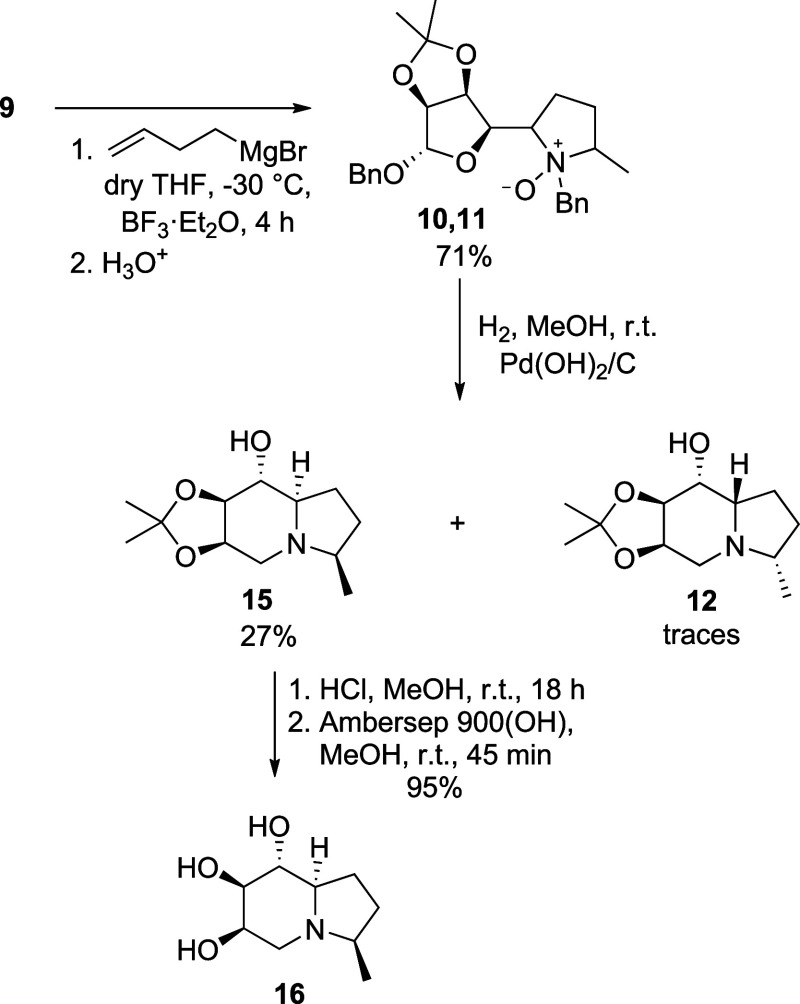
Synthesis of d-Mannose-Derived Polyhydroxylated 3-Methylindolizidine **16**

Consequently, the mixture was subjected to catalytic hydrogenation that furnished the two diastereomeric indolizidines **12** as the minor (traces) and **15** as the major one, whose structure was ascertained by 1D-NOESY spectra. The absolute configuration at C-8a proves the inverted stereoselectivity of the addition of the Grignard reagent in the presence of Lewis acids, and the relative *cis* C-3/C-8a configuration confirms the preferred antiperiplanar arrangement between the bulky sugar moiety and *N*-benzyl substituent in the Cope–House transition state (Figure 3). Nonetheless, the reductive amination step gave a lower yield compared to that of the one involving *N*-oxide **10** alone, suggesting competing or degradation phenomena. Final acidic deprotection and basic neutralization gave 3-methylindolizidine **16** (Scheme 2).

In order to study the scope of the process, the same domino procedure was applied to nitrone **17** (Scheme 3),^28^ readily obtained from commercially available diacetonide-d-glucose, which might furnish an indolizidine possessing the same absolute configuration as castanospermine. However, the addition of 3-butenylmagnesium bromide to **17** in THF at −78 °C gave a very complex mixture of products, and further reactions gave disappointing results. A low degree of stereoselectivity in the addition of other Grignard reagents to **17** which may justify the observed outcome has previously been observed, as well as a beneficial enhancement when using Lewis acids as additives.^29^ Consequently, it was deemed reasonable to use BF_3_·Et_2_O or Et_2_AlCl to simplify the reaction mixture. Better results were obtained with the latter, giving a higher yield and less complicated NMR spectra. As in the case of nitrone **9**, a domino addition/Cope–House cyclization occurred rapidly from **17** on quenching the Grignard addition without other manipulations of the crude reaction mixture, as suggested by the formation of highly polar compounds at the TLC control. An inseparable mixture of *N*-oxides was obtained, but no assignment could be done due to the complexity of the spectrum. In this case, the presence of signals of unsaturated *N*-hydroxylamine **20** suggested formation of the two diastereomeric *N*-oxides, **18** and **19**, in equilibrium through the open form. The (*R*) absolute configuration at C-5 of the *N*-oxides was assumed on the basis of the literature^29a^ and confirmed at a later stage. Stirring in CHCl_3_ or CH_3_OH for days did not alter the ratio among products or favor the cyclization.

**Scheme 3:**
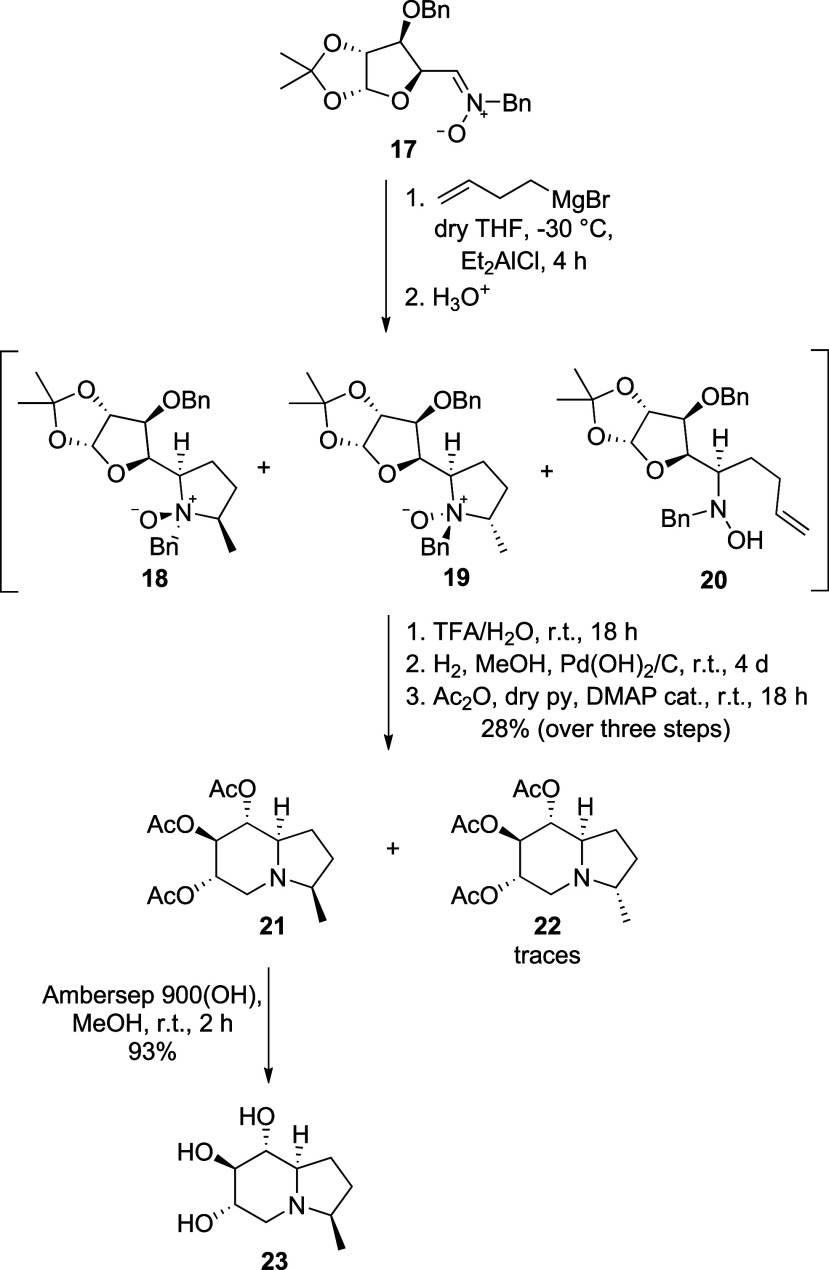
Synthesis of d-Glucose-Derived 3-Methyl-1-deoxy Castanospermine **23**

Deprotection of the acetonide functionality,^29b,30^ hydrogenation of the crude reaction mixture, and acetylation of free hydroxy groups gave indolizidine **21** as the major product, with the epimer **22** formed in traces and isolated in very low amounts after combining the products from a few reaction runs. Both compounds display the same absolute configuration at C-8a, deriving from a preferred attack of 3-butenylmagnesium bromide to the *Re* face,^27,29a^ confirming the high diastereoselectivity of the addition. Final removal of the acetyl groups in **21** furnished 3-methyl-1-deoxy castanospermine **23**.

In conclusion, a concise and stereoselective route to polyhydroxylated 3-methylindolizidines, an unprecedented family of castanospermine analogs, has been developed, starting from readily available inexpensive sugar-based nitrones and taking advantage of a domino organometallic addition/Cope–House process to build rapidly and stereoselectively the methyl pyrrolidine moiety. Although the yields of the key domino addition/Cope–House reaction are only moderate, the conciseness of the process allows to obtain the final indolizidines in 3 or 4 synthetic steps from the starting nitrones (readily accessed in very high yield from d-mannose and d-glucose) and 18–30% overall yields, which compare very well with respect to previous syntheses of castanospermine and its derivatives.^16^ The process described herein is likely the shortest and most sustainable for accessing a polyhydroxyindolizidine nucleus from a starting carbohydrate derivative. Further studies are ongoing to investigate the scope and mechanistic details of the process and the biological properties of the novel compounds.

## Supplementary Material



## Data Availability

The data underlying this study are available in the published article and its Supporting Information.

## Notes

Detailed experimental procedures, 1D and 2D NMR spectra of the compounds, and crystallographic data (PDF)

## †

Departamento de Química Orgánica y Farmacéutica, Facultad de Farmacia, Universidad de Sevilla, C/Prof. García González, 2, 41012 Sevilla, Spain
