# Supplementary material for: Domino Grignard Addition/Cope–House Reaction for the Synthesis of Polyhydroxylated 3‑Methylindolizidines Analogous to Castanospermine
Source: Org Lett. 2025 Sep 2;27(36):10098–104. doi: 10.1021/acs.orglett.5c03187 (PMC12442074; doi:10.1021/acs.orglett.5c03187)
Supplement: Supplementary file 1 [file ol5c03187_si_001.pdf]

SUPPORTING INFORMATION

Domino Grignard Addition/Cope-House Reaction for the Synthesis of Polyhydroxylated 3-Methylindolizidines Analogous to Castanospermine

Thomas Lulli, Filippo Dei, Macarena Martínez-Bailén†, Camilla Matassini, Cristina Faggi, Francesca Cardona, Andrea Goti*

Dipartimento di Chimica "Ugo Schiff" (DICUS), Università di Firenze, 50019 Sesto Fiorentino (FI), Italy.

† *Present address: Departamento de Química Orgánica y Farmacéutica, Facultad de Farmacia, Universidad de Sevilla, C/Prof. García González, 2, 41012 Sevilla, Spain.*

TABLE OF CONTENTS

1. **Experimental section**……………………………………………………………...S1
   1.1. General methods……………………………………………………………S1
   1.2. Detailed experimental procedures…………………………………………….S2
2. **NMR Spectra for compounds (^{1}H, ^{13}C{^{1}H}, gCOSY, gHSQC, gHMBC, and 1D NOESY)**……………………………………………………………………………..S10
3. **Crystal structure determination for compounds 13 and 14**………………………..S34
4. **References**……………………………………………………………………………..S49

1. Experimental section

1.1. General methods

Commercial reagents were used as received. All reactions were carried out under magnetic stirring and monitored by TLC on 0.25 mm silica gel plates (Merck F254). Column chromatographies were carried out on Silica Gel 60 (32–63 μm) or on silica gel (230–400 mesh, Merck, Darmstadt, Germany). Yields refer to spectroscopically and analytically pure compounds unless otherwise stated. The following abbreviations were used to designate typical solvents and reagents: Ac_2O = acetic anhydride; EtOAc = ethyl acetate; AcOH = acetic acid; DCM = dichloromethane; DMAP = 4-dimethylaminopyridine; PEt = petroleum ether; TFA = trifluoroacetic acid; THF = tetrahydrofuran. ^{1}H and ^{13}C{^{1}H} NMR spectra were recorded on a Varian Mercury 400 MHz, or a Varian INOVA 400 MHz instrument at 25 ºC for solutions in $CDCl_3$, CD_3OD. δ are given in ppm and *J* in Hz. Chemical shifts are reported relative to $CDCl_3$ (^{1}H: δ = 7.26 ppm; ^{13}C: δ = 77.0 ppm) or to CD_3OD (^{1}H: δ = 3.31 ppm; ^{13}C: δ = 49.0 ppm). Structural assignments were made with additional information from gCOSY and gHSQC experiments, recorded on a Varian Mercury 400 MHz or a Varian INOVA 400 MHz instrument. 1D NOESY and gHMBC experiments were performed when necessary. The following abbreviations were used to designate multiplicities: s = singlet, d = doublet, t = triplet, q = quartet, sext = sextet, m = multiplet, dd = doublet of doublets, td = triplet of doublets, qt = quartet of triplets, etc., br. = broad, ap. = apparent. IR spectra were recorded with a Shimadzu IRAffinity-1 or -1S spectrophotometer. ESI-MS spectra were recorded with a Thermo Scientific LCQ fleet ion trap mass spectrometer. Elemental analyses were performed with a Thermoscientific FlashSmart Elemental Analyzer CHNS/O. Optical rotation measurements were performed on a JASCO DIP-370 polarimeter. X-ray structural analyses were performed with a Bruker Apex-II CCD diffractometer.

1.2. Detailed experimental procedures

Synthesis of *N*-oxides 10 and 11

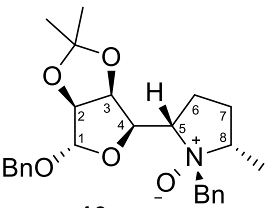

10

To a solution of nitrone **9**[1] (0.201 g, 0.523 mmol) in anhydrous THF (17 mL, 0.03 M), 3-butenylmagnesium bromide (0.5 M in THF, 3.1 mL, 1.550 mmol) was added dropwise at -78 °C, and the resulting reaction mixture was stirred at -78 °C for 4 hours. After this time, a saturated solution of NH_4Cl in water (10 mL) is added and stirred at r.t. for 10/15 minutes or overnight. The aqueous phase was extracted with DCM (3 x 10 mL), and the combined organic phases were washed with brine (1 x 20 mL), dried over Na_2SO_4, filtered, and concentrated under reduced pressure. The residue was purified by column chromatography on silica gel (EtOAc/CH_3OH 6:1) to give 0.071 g (0.162 mmol, 31%) of **10** as a yellowish oil, and 0.120 g (0.273 mmol, 52%) of a complex mixture of diastereoisomers.

Data for compound 10: $[\alpha]_D^{25}$ +41.3 (*c* 0.7, $CHCl_3$). IR ($CHCl_3$): $\tilde{\nu}$ 2936, 1220, 1211, 1080, 926 cm^{-1}. ^{1}H-NMR (400 MHz, $CDCl_3$): δ_H = 7.66-7.64 (m, 2H, NCH_2Ph), 7.40-7.24 (m, 8H, NCH_2Ph, OCH_2Ph), 5.24 (s, 1H, H-1), 5.10 (d, *J* = 13.2 Hz, 1H, NCH_2Ph), 5.05 (dd, *J* = 9.2 Hz, 3.2 Hz, 1H, H-4), 4.88 (d, *J* = 11.6 Hz, 1H, OCH_2Ph), 4.71 (dd, *J* = 6.0 Hz, 3.2 Hz, 1H, H-3), 4.67 (d, *J* = 6.0 Hz, 1H, H-2), 4.56 (d, *J* = 11.6 Hz, 1H, OCH_2Ph), 4.35 (d, *J* = 13.2 Hz, 1H, NCH_2Ph), 3.81 (ap.q, *J* = 8.9 Hz, 1H, H-5), 3.34-3.25 (m, 1H, H-8), 2.00-1.75 (m, 4H, H-6' and H-6'', H-7' and H-7''), 1.53 (d, *J* = 6.0 Hz, 3H, (C-8)-CH_3), 1.45 (s, 3H, $(CH_3)_2C_q$), 1.26 (s, 3H, $(CH_3)_2C_q$) ppm. $^{13}C\{^1H\}$-NMR (100 MHz, $CDCl_3$): δ_C = 137.1 (C, 1C, C_qPh), 132.8 (CH, 2C, NCH_2Ph), 131.7 (C, 1C, C_qPh), 129.3 (CH, 1C, Ph), 128.7 (CH, 2C, Ph), 128.5 (CH, 2C, Ph), 128.4 (CH, 2C, Ph), 127.8 (CH, 1C, Ph), 112.2 (C, 1C, $(CH_3)_2C_q$), 104.9 (CH, 1C, C-1), 84.1 (CH, 1C, C-2), 81.3 (CH, 1C, C-3), 78.1 (CH, 1C, C-4), 69.4 (CH, 1C, C-5), 68.6 (CH_2, 1C, OCH_2Ph), 67.4 (CH_2, 1C, NCH_2Ph), 67.1 (CH, 1C, C-8), 27.6 (CH_2, 1C, C-7), 26.2 (CH_3, 1C, $(CH_3)_2C_q$), 24.5 (CH_3, 1C, $(CH_3)_2C_q$), 22.6 (CH_2, 1C, C-6), 12.6 (CH_3, 1C, C8-CH_3) ppm. Anal Calcd. for $C_{26}H_{33}NO_5$: C, 71.05; H, 7.57; N, 3.19. Found: C, 71.25; H, 7.74; N, 3.14. MS (ESI) m/z : $[M + H]^+$ Calcd. for $C_{26}H_{33}NO_5$ 440.24; Found 440.17.

Synthesis of protected indolizidine 12 and by-product 13

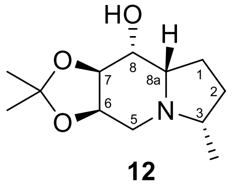

12

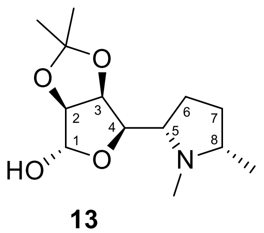

13

Procedure with Pd/C as catalyst and AcOH: To a solution of *N*-oxide **10** (0.100 g, 0.228 mmol) in dry CH_3OH (12 mL, 0.02 M), AcOH (0.04 mL, 0.700 mmol) and Pd/C (0.050 g) were added,

and the reaction mixture was stirred under an atmosphere of hydrogen for 8 days. The mixture was filtered through Celite(R), and the solvent was removed under reduced pressure. The resulting residue was purified by column chromatography on silica gel (DCM/CH_3OH/NH_3 (32%) 10:1:0.05 to 7:1:0.05 to 5:1:0.05) to give 0.020 g (0.086 mmol, 38%) of **12** as a yellowish oil and 0.015 g (0.057 mmol, 24%) of **13** as a white solid. **13** was then dissolved in CH_3OH and slow solvent evaporation gave a crystalline compound suitable for X-Ray analysis.

Procedure with $Pd(OH)_2/C$ as catalyst: To a solution of *N*-oxide **10** (0.045 g, 0.102 mmol) in dry CH_3OH (5 mL), $Pd(OH)_2/C$ (0.022 g) was added, and the reaction mixture was stirred under an atmosphere of hydrogen for 4 days. The mixture was filtered through Celite(R), and the solvent was removed under reduced pressure. The resulting residue was purified by flash column chromatography on silica gel (DCM/CH_3OH 20:1) to give 0.014 g of **12** (0.062 mmol, 61%) as a yellowish oil.

Data for compound 12: $[\alpha]_D^{24}$ +80.6 (*c* 0.3, CH_3OH). IR (CH_3OH): $\tilde{\nu}$ 3441, 2932, 1379, 1217, 1055, 861 cm^{-1}. ^1H-NMR (400 MHz, CD_3OD): δ_H = 4.31 (ddd, *J* = 9.6 Hz, 7.0 Hz, 5.0 Hz, 1H, H-6), 4.17 (dd, *J* = 5.0 Hz, 2.2. Hz, 1H, H-7), 3.88 (ap.t, *J* = 2.2 Hz, 1H, H-8), 3.14 (dd, *J* = 10.4 Hz, 7.2 Hz, 1H, H-5_{eq}), 2.45 (ap.t, *J* = 8.0 Hz, 1H, H-8a), 2.23 (qt, *J* = 6.4 Hz, 4.0 Hz, 1H, H-3), 1.98-1.87 (m, 2H, H-1', H-2'), 1.86 (ap.t, *J* = 9.4 Hz, 1H, H-5_{ax}), 1.69-1.59 (m, 1H, H-1''), 1.46 (s, 3H, ($C\underline{H}_3$)$_2C_q$), 1.40-1.29 (m, 1H, H-2''), 1.34 (s, 3H, ($C\underline{H}_3$)$_2C_q$), 1.08 (d, *J* = 6.4 Hz, 3H, (C-3)-$C\underline{H}_3$) ppm. $^{13}C\{^1H\}$-NMR (100 MHz, CD_3OD): δ_C = 110.0 (C, 1C, $(CH_3)_2\underline{C}_q$), 78.5 (CH, 1C, C-7), 72.8 (CH, 1C, C-6), 67.3 (CH, 1C, C-8), 65.6 (CH, 1C, C-8a), 61.8 (CH, 1C, C-3), 55.1 (CH_2, 1C, C-5), 32.3 (CH_2, 1C, C-2), 28.5 (CH_3, 1C, ($\underline{C}H_3$)$_2C_q$), 26.5 (CH_3, 1C, ($\underline{C}H_3$)$_2C_q$), 22.7 (CH_2, 1C, C-1), 18.0 (CH_3, 1C, (C-3)-$\underline{C}H_3$) ppm. Anal. Calcd for $C_{12}H_{21}NO_3$: C, 63.41; H, 9.31; N, 6.16. Found: C, 63.35; H, 9.33; N, 6.24. MS (ESI) m/z : $[M + H]^+$ Calcd. for $C_{12}H_{21}NO_3$ 228.16; Found 228.08.

Data for compound 13: m.p. 143-146 °C. $[\alpha]_D^{23}$ +5.0 (*c* 1.2, CH_3OH). IR (CH_3OH): $\tilde{\nu}$ 2972, 2936, 1373, 1207, 1075, 888 cm^{-1}. ^1H-NMR (400 MHz, CD_3OD): δ_H = 5.25 (s, 1H, H-1), 4.72 (dd, *J* = 5.8 Hz, 3.4 Hz, 1H, H-3), 4.47 (d, *J* = 6.0 Hz, 1H, H-2), 4.06 (dd, *J* = 9.0 Hz, 3.4 Hz, 1H, H-4), 2.68 (ap.q, *J* = 8.5 Hz, 1H, H-5), 2.46 (s, 3H, $NC\underline{H}_3$), 2.37-2.28 (m, 1H, H-8), 2.06 (dddd, *J* = 12.8 Hz, 9.6 Hz, 8.2 Hz, 6.6 Hz, 1H, H-6'), 1.93 (dddd, *J* = 11.8 Hz, 10.2 Hz, 6.6 Hz, 3.8 Hz, 1H, H-7'), 1.59-1.49 (m, 1H, H-6''), 1.46-1.34 (m, 1H, H-7''), 1.41 (s, 3H, ($C\underline{H}_3$)$_2C_q$), 1.28 (s, 3H, ($C\underline{H}_3$)$_2C_q$), 1.13 (d, *J* = 6.0 Hz, 3H, (C-8)-$C\underline{H}_3$) ppm. $^{13}C\{^1H\}$-NMR (100 MHz, CD_3OD): δ_C = 113.3 (C, 1C, $(CH_3)_2\underline{C}_q$), 102.6 (CH, 1C, C-1), 86.5 (CH, 1C, C-2), 85.1 (CH, 1C, C-4), 82.8 (CH, 1C, C-3), 67.7 (CH, 1C, C-5), 64.9 (CH, 1C, C-8), 40.7 (CH_3, 1C, $N\underline{C}H_3$), 32.4 (CH_2, 1C, C-7), 26.4 (CH_3, 1C, ($\underline{C}H_3$)$_2C_q$), 26.1 (CH_2, 1C, C-6), 25.0 (CH_3, 1C, ($\underline{C}H_3$)$_2C_q$), 18.3 (CH_3, 1C, (C-8)-$\underline{C}H_3$) ppm. Anal. Calcd for $C_{13}H_{23}NO_4$: C, 60.68; H, 9.01; N, 5.44. Found: C, 60.83; H, 8.93; N, 5.29. MS (ESI) m/z : $[M + H]^+$ Calcd. for $C_{13}H_{23}NO_4$ 258.17; Found 258.17.

Synthesis of indolizidine 14:

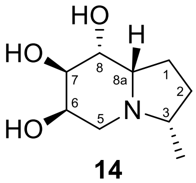

14

To a solution of protected indolizidine **12** (0.014 g, 0.070 mmol) in CH_3OH (4.7 mL, 0.015 M) two drops of 12 M HCl were added, and the solution was left stirring at room temperature for 18 h. The crude mixture was concentrated to yield the hydrochloride salt of the final indolizidine **14**. The corresponding free amine was obtained by dissolving the residue in CH_3OH, then the strongly basic resin Ambersep 900-OH was added, and the mixture was stirred for 2 hours. The resin was removed by filtration, and the crude product was purified on silica gel by flash column chromatography (DCM/CH_3OH/NH_3 (32%) 7:1:0.05) to afford 0.012 g (0.065 mmol, 91%) of **14** as a white solid. **14** was then dissolved in CH_3OH, and slow solvent evaporation gave a crystalline compound suitable for X-ray analysis.

Data for compound 14: m.p. 136-139 °C. $[\alpha]_D^{19}$ +56.0 (*c* 0.7, CH_3OH). IR (neat): $\tilde{\nu}$ 3389, 3332, 2900, 2823, 1393, 1060, 938 cm^{-1}. ^{1}H-NMR (400 MHz, CD_3OD): δ_H = 3.96 (ddd, *J* = 10.8 Hz, 5.2 Hz, 3.2 Hz, 1H, H-6), 3.84 (ap.t, *J* = 3.4 Hz, 1H, H-7), 3.67 (dd, *J* = 3.4 Hz, 1H, H-8), 2.92 (dd, *J* = 9.8 Hz, 5.2 Hz, 1H, H-5_{eq}), 2.53 (ddd, *J* = 9.6 Hz, 7.2 Hz, 1.4 Hz, 1H, H-8a), 2.26 (tq, *J* = 8.4 Hz, 6.0 Hz, 1H, H-3), 2.06 (ap.t, *J* = 6.4 Hz, 1H, H-5_{ax}), 1.94 (dddd, *J* = 12.4 Hz, 10.0 Hz, 7.6 Hz, 6.0 Hz, 1H, H-2'), 1.79 (dddd, *J* = 12.6 Hz, 12.0 Hz, 10.0 Hz, 5.8 Hz, 1H, H-1'), 1.55 (dddd, *J* = 12.0 Hz, 10.4 Hz, 7.2 Hz, 4.8 Hz, 1H, H-1''), 1.34 (dddd, *J* = 12.2 Hz, 12.0 Hz, 9.2 Hz, 4.6 Hz, 1H, H-2''), 1.10 (d, *J* = 6.0 Hz, 3H, (C-3)-C$\underline{H}_3$) ppm. ^{13}C{^{1}H}-NMR (100 MHz, CD_3OD): δ_C = 72.5 (CH, 1C, C-7), 70.6 (CH, 1C, C-8), 67.6 (CH, 1C, C-6), 63.6 (CH, 1C, C-8a), 61.4 (CH, 1C, C-3), 52.4 (CH_2, 1C, C-5), 32.2 (CH_2, 1C, C-2), 22.8 (CH_2, 1C, C-1), 17.9 (CH_3, 1C, (C-3)-C$\underline{H}_3$) ppm. Anal. Calcd for $C_9H_{17}NO_3$: C, 57.73; H, 9.15; N, 7.48. Found: C, 57.69; H, 9.18; N, 7.54. MS (ESI) m/z : $[M + H]^+$ Calcd. for $C_9H_{17}NO_3$ 188.13; Found 188.08.

General procedure for the synthesis of the mixture of N-oxides 10 and 11 with Lewis acid

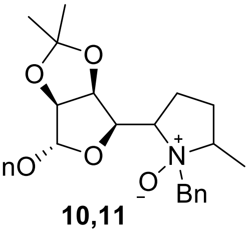

10,11

To a solution of nitrone **9**[1] (1 eq) in anhydrous THF (0.03 M), Lewis acid (1.1 eq) was added dropwise. After stirring for 20 minutes at r.t., the solution was cooled to -30 °C, and 3-butenylmagnesium bromide (0.5 M in THF, 3 eq.) was added dropwise; the resulting reaction mixture was stirred at -30 °C for 4 hours. After this time, a saturated solution of NH_4Cl in water (15 mL) is added and stirred at r.t. for 10/15 minutes or overnight. The aqueous phase was

extracted with DCM (3 x 10 mL), and the combined organic phases were washed with brine (1 x 20 mL), dried over Na_2SO_4, filtered, and concentrated under reduced pressure. The residue was purified by column chromatography on silica gel (EtOAc/CH_3OH 6:1) to give a mixture of **10**, **11**. No assignment in the ^{1}H-NMR spectrum was possible, and the mixture of *N*-oxides was used in the subsequent reductive amination step.

Synthesis of the mixture of *N*-oxides 10 and 11 with $BF_3·Et_2O$

The general procedure was applied to nitrone **9** (0.152 g, 0.396 mmol) in anhydrous THF (14 mL, 0.03 M), and $BF_3·Et_2O$ (0.06 mL, 0.436 mmol) was used as Lewis acid. 2.4 mL of a 0.5 M solution (1.20 mmol) of 3-butenylmagnesium bromide in THF was added dropwise at -30 °C. Following the general quenching and work-up procedure, the residue was purified by column chromatography on silica gel (EtOAc/CH_3OH 6:1) to give 0.123 g (0.280 mmol, 71%, yellowish sticky oil) of **10**, **11**.

Synthesis of the mixture of *N*-oxides 10 and 11 with Et_2AlCl

The general procedure was applied to nitrone **9** (0.156 g, 0.407 mmol) in anhydrous THF (14 mL, 0.03 M), and Et_2AlCl (1.0 M in hexane, 0.45 mL, 0.450 mmol) was used as Lewis acid. 2.4 mL of a 0.5 M solution (1.20 mmol) of 3-butenylmagnesium bromide in THF was added dropwise at -30 °C. Following the general quenching and work-up procedure, the residue was purified by column chromatography on silica gel (EtOAc/CH_3OH 6:1) to give 0.088 g (0.200 mmol, 50%, yellowish sticky oil) of **10**, **11**.

Synthesis of protected indolizidine 15

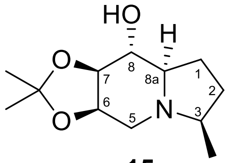

15

To a solution of the mixture of *N*-oxides **10**, **11** (0.094 g, 0.214 mmol) in dry CH_3OH (14 mL), $Pd(OH)_2/C$ (0.050 g) was added, and the reaction mixture was stirred under an atmosphere of hydrogen for 4 days. The mixture was filtered through Celite(R), and the solvent was removed under reduced pressure. The resulting residue was purified by column chromatography on silica gel (PEt/EtOAc/CH_3OH 10:5:1) to give 0.013 g of **15** (0.057 mmol, 27%, yellowish oil).

Data for compound 15: $[\alpha]_D^{22}$ – 92.0 (*c* 0.8, CH_3OH). IR (CH_3OH): $\tilde{\nu}$ 3390, 2937, 2503, 2065, 1658, 1456, 1379, 1024, 847 cm^{-1}. ^{1}H-NMR (400 MHz, CD_3OD): δ_H = 4.36 (ddd, *J* = 5.2 Hz, 3.6 Hz, 1.6 Hz, 1H, H-6), 3.81 (dd, *J* = 13.0 Hz, 1.0 Hz, 1H, H-5$_{eq}$), 3.45 (dd, *J* = 7.0 Hz, 2.6 Hz, 1H, H-8), 2.33-2.25 (m, 1H, H-3), 2.23 (dd, *J* = 13.2 Hz, 3.2 Hz, 1H, H-5$_{ax}$), 2.00-1.88 (m, 3H, H-1', H-2', H-8a), 1.55-1.42 (m, 2H, H-1'', H-2''), 1.52 (s, 3H, $(C\underline{H}_3)_2C_q$), 1.35 (s, 3H, $(C\underline{H}_3)_2C_q$), 1.14 (d, *J* = 6.0 Hz, 3H, (C-3)-$C\underline{H}_3$) ppm. $^{13}C\{^1H\}$-NMR (100 MHz, CD_3OD): δ_C = 110.3 (C, 1C, $(CH_3)_2\underline{C}_q$), 82.0 (CH, 1C, C-7), 77.4 (CH, 1C, C-6), 75.9 (CH, 1C, C-8), 68.8 (CH, 1C, C-8a), 62.8 (CH, 1C, C-3), 52.1 (CH_2, 1C, C-5), 31.9 (CH_2, 1C, C-2), 28.3 (CH_3, 1C, $(\underline{C}H_3)_2C_q$), 27.0 (CH_2, 1C, C-1), 26.5 (CH_3, 1C, $(\underline{C}H_3)_2C_q$), 18.0 (CH_3, 1C, (C-3)-$C\underline{H}_3$) ppm. Anal. Calcd for $C_{12}H_{21}NO_3$: C, 63.41; H, 9.31; N, 6.16. Found: C, 63.38; H, 9.29; N, 6.19. MS (ESI) m/z : $[M + H]^+$ Calcd. for $C_{12}H_{21}NO_3$ 228.16; Found 228.08.

Synthesis of indolizidine 16:

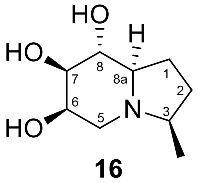

16

To a solution of protected indolizidine **15** (0.023 g, 0.101 mmol) in CH_3OH (6.5 mL, 0.015 M), two drops of 12 M HCl were added, and the solution was left stirring at room temperature for 18 h. The crude mixture was concentrated to yield the hydrochloride salt of the final indolizidine **16**. The corresponding free amine was obtained by dissolving the residue in CH_3OH, then the strongly basic resin Ambersep 900-OH was added, and the mixture was stirred for 2 hours. The resin was removed by filtration and the crude product was purified on silica gel by flash column chromatography (DCM/CH_3OH/NH_3 (32%) 3:1:0.01) to afford 0.018 g (0.096 mmol, 95%) of **16** as a transparent oil.

Data for compound 16: $[\alpha]_D^{19}$ – 63.4 (*c* 0.9, CH_3OH). IR (neat): $\tilde{\nu}$ 3331, 2960, 2872, 2797, 1379, 1092, 1057 cm^{-1}. ^{1}H-NMR (400 MHz, CD_3OD): δ_H = 3.91 (ddd, *J* = 3.2 Hz, 3.0 Hz, 1.8 Hz, 1H, H-6), 3.48 (ap.t, *J* = 9.2 Hz, 1H, H-8), 3.30 (dd, *J* = 9.2 Hz, 3.6 Hz, 1H, H-7), 3.15 (dd, *J* = 12.0 Hz, 2.8 Hz, 1H, H-5_{eq}), 2.30 (dqd, *J* = 8.4 Hz, 6.0 Hz, 6.0 Hz, 1H, H-3), 2.06 (dd, *J* = 11.8 Hz, 1.8 Hz, 1H, H-5_{ax}), 2.00-1.89 (m, 3H, H-1', H-2', H-8a), 1.62-1.52 (m, 1H, H-1''), 1.48-1.40 (m, 1H, H-2''), 1.10 (d, *J* = 6.0 Hz, 3H, (C-3)-C$\underline{H}_3$) ppm. ^{13}C{^{1}H}-NMR (100 MHz, CD_3OD): δ_C = 77.3 (CH, 1C, C-7), 75.0 (CH, 1C, C-8), 70.5 (CH, 1C, C-6), 70.4 (CH, 1C, C-8a), 61.4 (CH, 1C, C-3), 55.4 (CH_2, 1C, C-5), 32.0 (CH_2, 1C, C-2), 27.1 (CH_2, 1C, C-1), 18.4 (CH_3, 1C, (C-3)-C$\underline{H}_3$) ppm. Anal. Calcd for $C_9H_{17}NO_3$: C, 57.73; H, 9.15; N, 7.48. Found C, 57.54; H, 9.35; N, 7.29. MS (ESI) m/z : = $[M + H]^+$ Calcd. for $C_9H_{17}NO_3$ 188.13; Found 188.08.

General procedure for the synthesis of the mixture of N-oxides 18 and 19 with Lewis acid

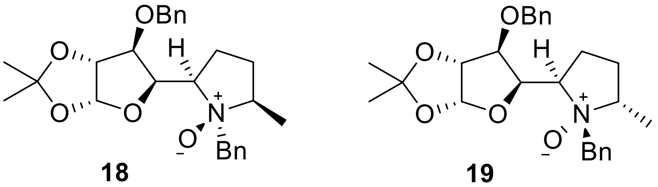

18 **19**

To a solution of nitrone **17**[2] (1 eq) in anhydrous THF (0.03 M), Lewis acid (1.1 eq) was added dropwise. After stirring for 20 minutes at r.t., the solution was cooled to -30 °C, and 3-butenylmagnesium bromide (0.5 M in THF, 3 eq.) was added dropwise; the resulting reaction mixture was stirred at -30 °C for 4 hours. After this time, a saturated solution of NH_4Cl in water (15 mL) is added and stirred at r.t. for 10/15 minutes or overnight. The aqueous phase was extracted with DCM (3 x 10 mL), and the combined organic phases were washed with brine (1 x 20 mL), dried over Na_2SO_4, filtered, and concentrated under reduced pressure. The residue was purified by column chromatography on silica gel (EtOAc/CH_3OH 5:1) to give a mixture

of **18** and **19**. No assignment in the ^{1}H-NMR spectrum was possible, and the mixture of *N*-oxides was used in the subsequent reductive amination step.

Synthesis of the mixture of *N*-oxides 18 and 19 with $BF_3 \cdot Et_2O$

The general procedure was applied to nitrone **17** (0.200 g, 0.522 mmol) in anhydrous THF (17 mL, 0.03 M), and $BF_3 \cdot Et_2O$ (0.07 mL, 0.574 mmol) was used as Lewis acid. 3.1 mL of a 0.5 M solution (1.550 mmol) of 3-butenylmagnesium bromide in THF was added dropwise at -30 °C. Following the general quenching and work-up procedure, the residue was purified by column chromatography on silica gel (EtOAc/CH_3OH 5:1) to give 0.110 g (0.256 mmol, 49%, sticky oil) of **18** and **19**.

Synthesis of the mixture of *N*-oxides 18 and 19 with Et_2AlCl

The general procedure was applied to nitrone **17** (0.200 g, 0.522 mmol) in anhydrous THF (17 mL, 0.03 M), and Et_2AlCl (1.0 M in hexane, 0.57 mL, 0.57 mmol) was used as Lewis acid. 3.1 mL of a 0.5 M solution (1.550 mmol) of 3-butenylmagnesium bromide in THF was added dropwise at -30 °C. Following the general quenching and work-up procedure, the residue was purified by column chromatography on silica gel (EtOAc/CH_3OH 5:1) to give 0.122 g (0.277 mmol, 53%, sticky oil) of **18** and **19**.

Synthesis of protected indolizidines 21 and 22

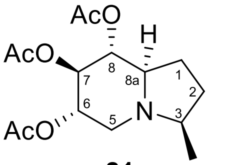

21

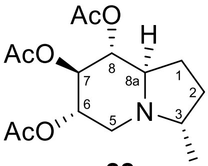

22

The mixture of *N*-oxides **18** and **19** (0.097 g, 0.2207 mmol) is dissolved in a TFA/H_2O 3:2 (2.2 mL, 0.10 M) mixture at 0 °C, stirred at such temperature for 15 minutes, and then left stirring at room temperature for 18 h. TFA was co-evaporated with toluene under reduced pressure to furnish the corresponding hemiacetal (brownish sticky oil). The crude reaction mixture was used in the subsequent reductive amination step without further purification.

The intermediates aldehydes were dissolved in dry CH_3OH (15 mL), $Pd(OH)_2/C$ (0.050 g) was added, and the reaction mixture was stirred under an atmosphere of hydrogen for 4 days. The mixture was filtered through Celite(R), and the solvent was removed under reduced pressure. The crude reaction mixture was then dissolved in anhydrous pyridine (0.3 mL); Ac_2O (0.2 mL), and a catalytic quantity of DMAP were added. The resulting mixture was left stirring for 18h at r.t. Pyridine was co-evaporated with toluene, and the resulting residue was purified by column chromatography on silica gel (PEt/EtOAc 3:1) to afford 0.019 g (0.061 mmol, 24%, white solid) of **21** as the major product (white solid). **22** (0.006 g, 0.020 mmol, yellowish oil) was also recovered as the minor product, albeit in a very low quantity and not sufficiently pure to be characterized. After combining a few attempts, **22** was purified by column chromatography (PEt/EtOAc 2:1), obtaining 0.008 g (0.026 mmol) as a yellowish oil

sufficiently pure to be characterized. Due to the very low quantity of **22**, the final deprotection of acetyl groups was not carried out.

Data for compound 21: m.p. 109-111 °C. $[\alpha]_D^{25}$ + 4.8 (*c* 0.9, $CHCl_3$). IR (neat): $\tilde{\nu}$ 2951, 2918, 2848, 1737, 1215, 1028 cm^{-1}. ^1H-NMR (400 MHz, $CDCl_3$): δ_H = 5.07-4.99 (m, 2H, H-6, H-7), 4.89 (ap.t, *J* = 9.2 Hz, 1H, H-8), 3.29 (dd, *J* = 10.4 Hz, 4.4 Hz, 1H, H-5_{eq}), 2.37 (tq, *J* = 7.7 Hz, 6.3 Hz, 1H, H-3), 2.28 (dt, *J* = 9.5 Hz, 6.9 Hz, 1H, H-8a), 2.01-2.00 (m, 9H, 3x$OCOC\underline{H}_3$), 1.97-1.86 (m, 2H, H-2', H-5_{ax}), 1.79-1.71 (m, 1H, H-1'), 1.54 (ap.tdd, *J* = 11.8 Hz, 9.6 Hz, 6.0 Hz, 1H, H-1''), 1.42 (ap.tdd, 12.2 Hz, 8.0 Hz, 3.6 Hz, 1H, H-2''), 1.06 (d, *J* = 6.0 Hz, 3H, (C-3)-$C\underline{H}_3$) ppm. $^{13}C\{^1H\}$-NMR (100 MHz, CD_3OD): δ_C = 170.7 (C, 1C, $O\underline{C}OCH_3$), 170.2 (C, 1C, $O\underline{C}OCH_3$), 170.1 (C, 1C, $O\underline{C}OCH_3$), 75.2 (CH, 1C, C-7), 74.4 (CH, 1C, C-6), 71.2 (CH, 1C, C-8), 66.4 (CH, 1C, C-8a), 59.4 (CH, 1C, C-3), 51.0 (CH_2, 1C, C-5), 31.3 (CH_2, 1C, C-1), 26.0 (CH_2, 1C, C-2), 21.0 (CH_3, 1C, $OCO\underline{C}H_3$), 20.9 (CH_3, 2C, 2x$OCO\underline{C}H_3$), 19.0 (CH_3, 1C, (C-3)-$\underline{C}H_3$) ppm. Anal. Calcd for $C_{15}H_{23}NO_6$: C, 57.50; H, 7.40; N, 4.47. Found: C, 57.37; H, 7.47; N, 4.37. MS (ESI) m/z : $[M + Na]^+$ Calcd. for $C_{15}H_{23}NO_6$ 336.14; Found 336.00.

Data for compound 22: $[\alpha]_D^{20}$ + 36.0 (*c* 1.0, $CHCl_3$). IR (neat): $\tilde{\nu}$ 2968, 2953, 1736, 1215, 1026 cm^{-1}. ^1H-NMR (400 MHz, $CDCl_3$): δ_H = 5.05 (ap.t., *J* = 9.0 Hz, 1H, H-7), 5.00 (ap.td, *J* = 9.2 Hz, 4.7 Hz, 1H, H-6), 4.85 (ap.t, *J* = 9.4 Hz, 1H, H-8), 3.33-3.27 (m, 1H, H-3), 3.24 (dd, *J* = 12.2 Hz, 4.6 Hz, 1H, H-5_{eq}), 2.90 (dt, *J* = 9.7 Hz, 6.1 Hz, 1H, H-8a), 2.56 (dd, *J* = 12.0 Hz, 10.0 Hz, 1H, H-5_{ax}), 2.17-1.85 (m, 11H, H-1', H-2', 3x$OCOC\underline{H}_3$), 1.61-1.52 (m, 1H, H-1''), 1.45-1.37 (m, 1H, H-2''), 0.98 (d, *J* = 6.0 Hz, 3H, (C-3)-$C\underline{H}_3$) ppm. $^{13}C\{^1H\}$-NMR (100 MHz, CD_3OD): δ_C = 170.5 (C, 1C, $O\underline{C}OCH_3$), 170.4 (C, 1C, $O\underline{C}OCH_3$), 170.3 (C, 1C, $O\underline{C}OCH_3$), 75.1 (CH, 1C, C-7), 73.2 (CH, 1C, C-6), 69.6 (CH, 1C, C-8), 61.5 (CH, 1C, C-8a), 55.5 (CH, 1C, C-3), 47.3 (CH_2, 1C, C-5), 31.2 (CH_2, 1C, C-1), 26.7 (CH_2, 1C, C-2), 21.0 (CH_3, 1C, $OCO\underline{C}H_3$), 20.9 (CH_3, 2C, 2x$OCO\underline{C}H_3$), 17.5 (CH_3, 1C, (C-3)-$C\underline{H}_3$) ppm. Anal. Calcd for : C, 57.50; H, 7.40; N, 4.47. Found: C, 57.39; H, 7.32; N, 4.60. MS (ESI) m/z : $[M + H]^+$ Calcd. for $C_{15}H_{23}NO_6$ 314.16; Found 314.08.

Synthesis of indolizidine 23

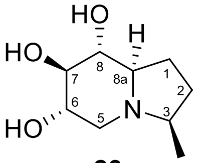

23

Compound **21** was dissolved in CH_3OH, and the strongly basic resin Ambersep 900-OH was added; the mixture was stirred for 3 hours. The resin was removed by filtration, and the crude product was purified on silica gel by flash column chromatography (DCM/CH_3OH/NH_3 (32%) 7:1:0.05) to afford 0.011 mg (0.059 mmol, quantitative yield) of **23** as a white solid.

Data for compound 23: m.p. 149-151 °C. $[\alpha]_D^{24}$ – 13.2 (*c* 0.8, CH_3OH). IR (neat): $\tilde{\nu}$ 3302, 2960, 2904, 2796, 1354, 1089, 1024, 1008 cm^{-1}. ^1H-NMR (400 MHz, CD_3OD): δ_H = 3.58 (ddd, *J* = 10.4 Hz, 8.4 Hz, 5.2 Hz, 1H, H-6), 3.20 (ap.t, *J* = 9.0 Hz, 1H, H-8), 3.18 (dd, *J* = 11.2 Hz, 5.2 Hz, 1H, H-5_{eq}), 3.15 (ap.t, *J* = 8.8 Hz, 1H, H-7), 2.38 (ap.sext, *J* = 6.6 Hz, 1H, H-3), 2.12-2.06 (m, 1H, H-8a), 2.04-1.93 (m, 2H, H-1', H-2'), 1.87 (t, *J* = 10.6 Hz, 1H, H-5_{ax}), 1.57-1.37 (m, 2H, H-1'', H-2''), 1.13 (d, *J* = 6.0 Hz, 3H, (C-3)-$C\underline{H}_3$) ppm. $^{13}C\{^1H\}$-NMR (100 MHz,

CD_3OD): δ_C = 80.8-76.4 (CH, 2C, C-7, C-8), 72.2 (CH, 1C, C-6), 70.2 (CH, 1C, C-8a), 61.4 (CH, 1C, C-3), 55.6 (CH_2, 1C, C-5), 32.0 (CH_2, 1C, C-2), 27.0 (CH_2, 1C, C-1), 18.3 (CH_3, 1C, (C-3)-$\underline{C}H_3$) ppm. Anal. Calcd for $C_9H_{17}NO_3$: C, 57.73; H, 9.15; N, 7.48. Found: C, 57.80; H, 9.18; N, 7.57. MS (ESI) m/z : $[M + Na]^+$ Calcd. for $C_9H_{17}NO_3$ 210.11; Found 210.00.

2. **NMR Spectra for compounds (^{1}H, ^{13}C{^{1}H}, gCOSY, gHSQC, gHMBC and 1D NOESY spectra)**

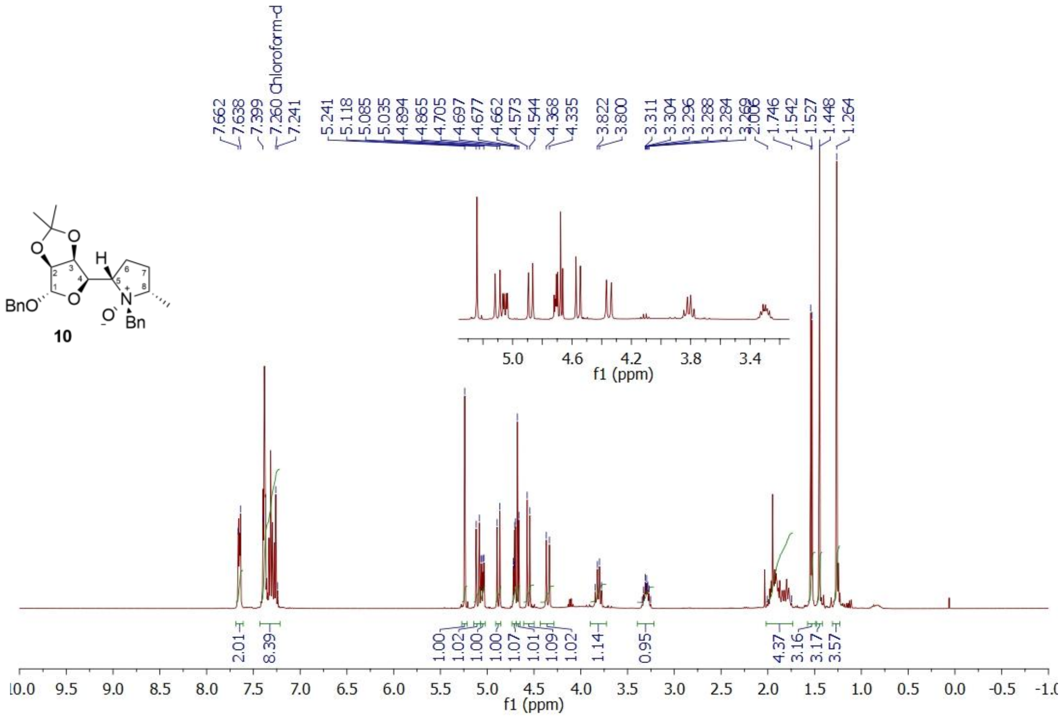

Figure S1. ^{1}H-NMR (400 MHz, $CDCl_3$) of compound **10**.

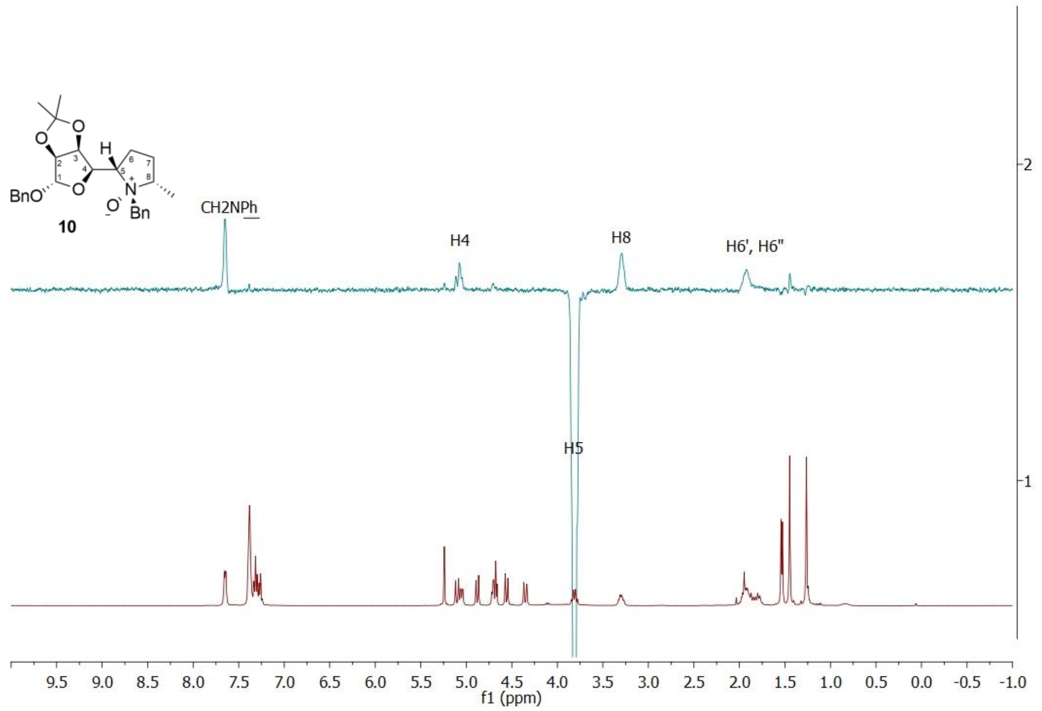

Figure S2. 1D-NOESY spectrum (400 MHz, $CDCl_3$) of compound **10**; selective irradiation of H-5 (3.81 ppm) gave NOE correlation with H-8.

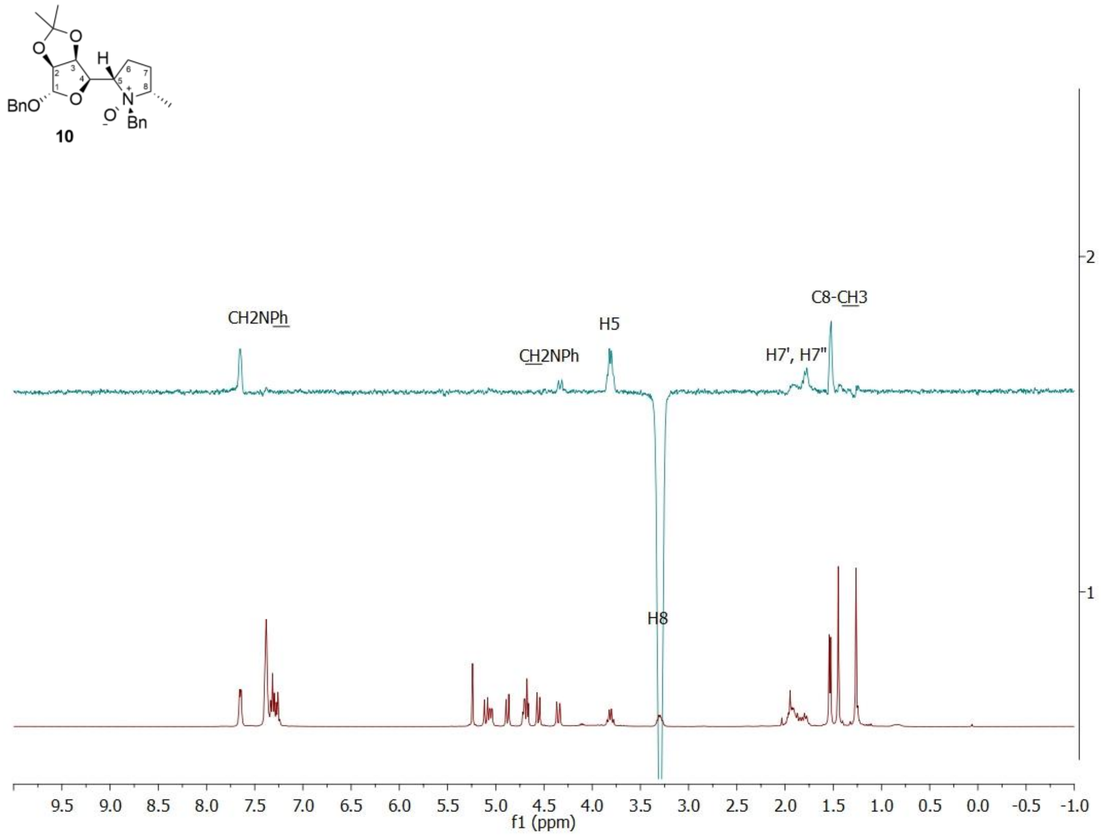

Figure S3. 1D-NOESY spectrum (400 MHz, $CDCl_3$) of compound **10**; selective irradiation of H-8 (3.34-3.25 ppm) gave NOE correlation with H-5.

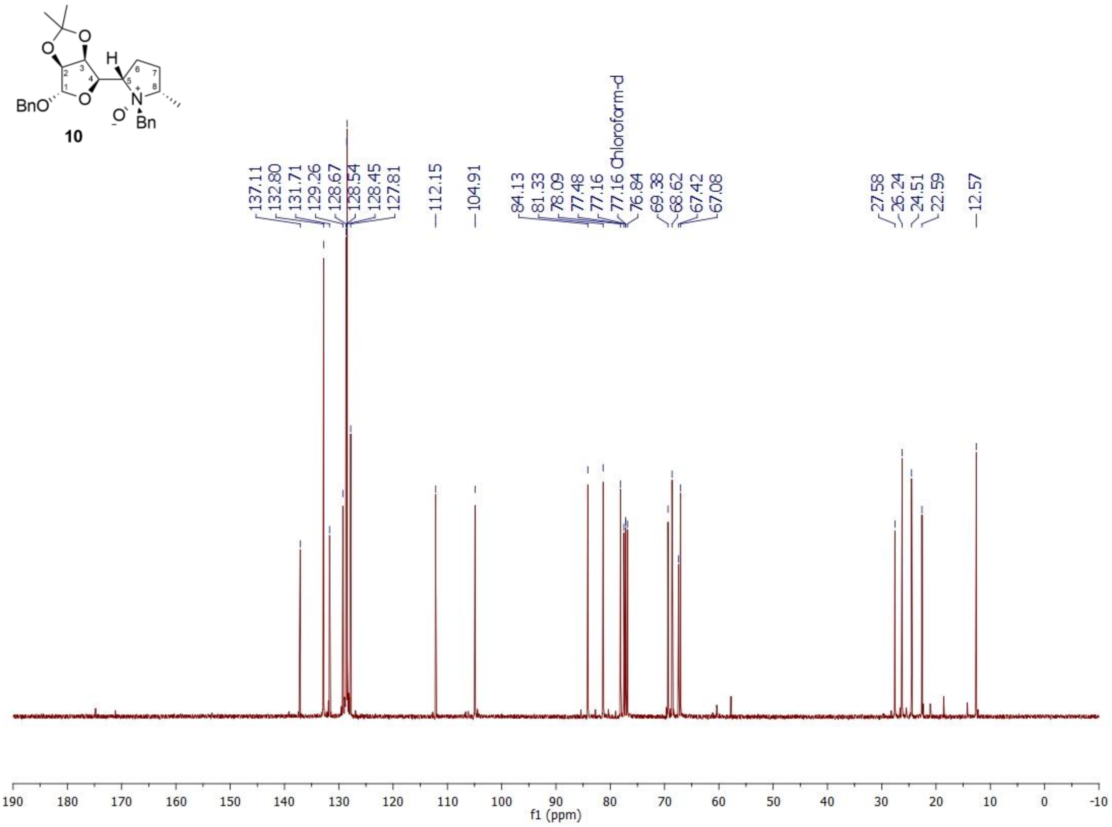

Figure S4. $^{13}C\{^1H\}$-NMR (100 MHz, $CDCl_3$) of compound **10**.

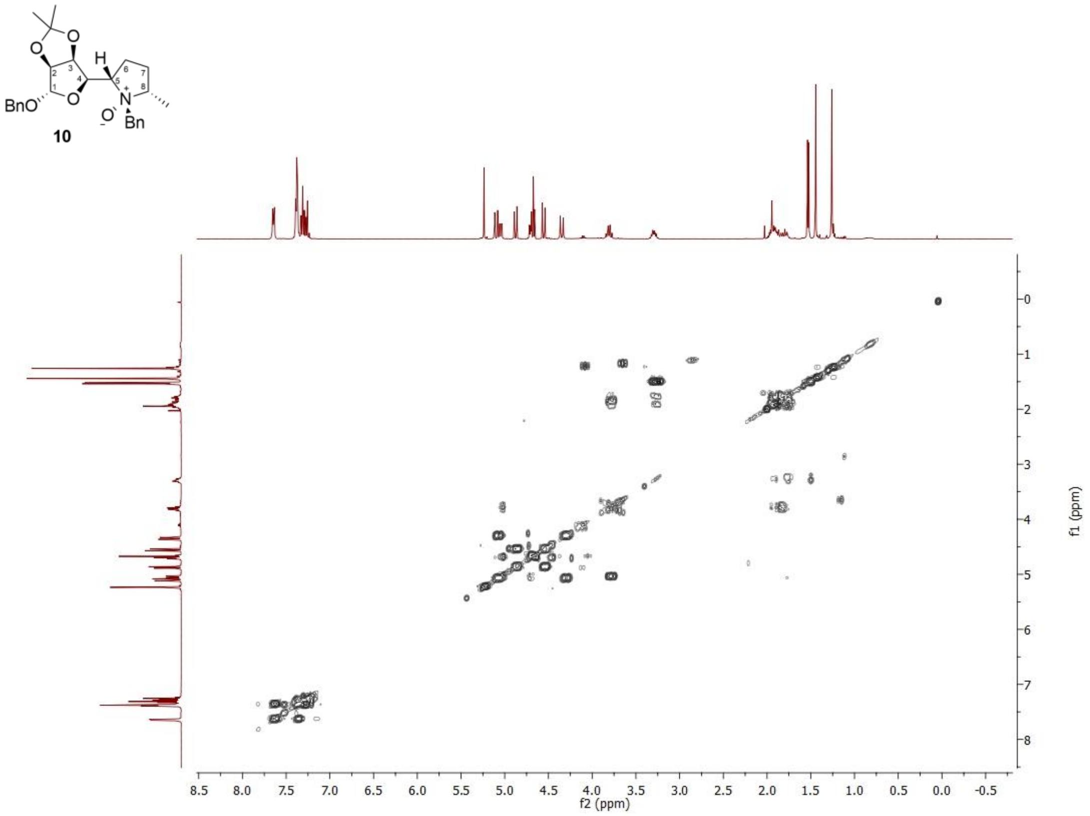

Figure S5. ^{1}H/^{1}H gCOSY (400 MHz, $CDCl_3$) of compound **10**.

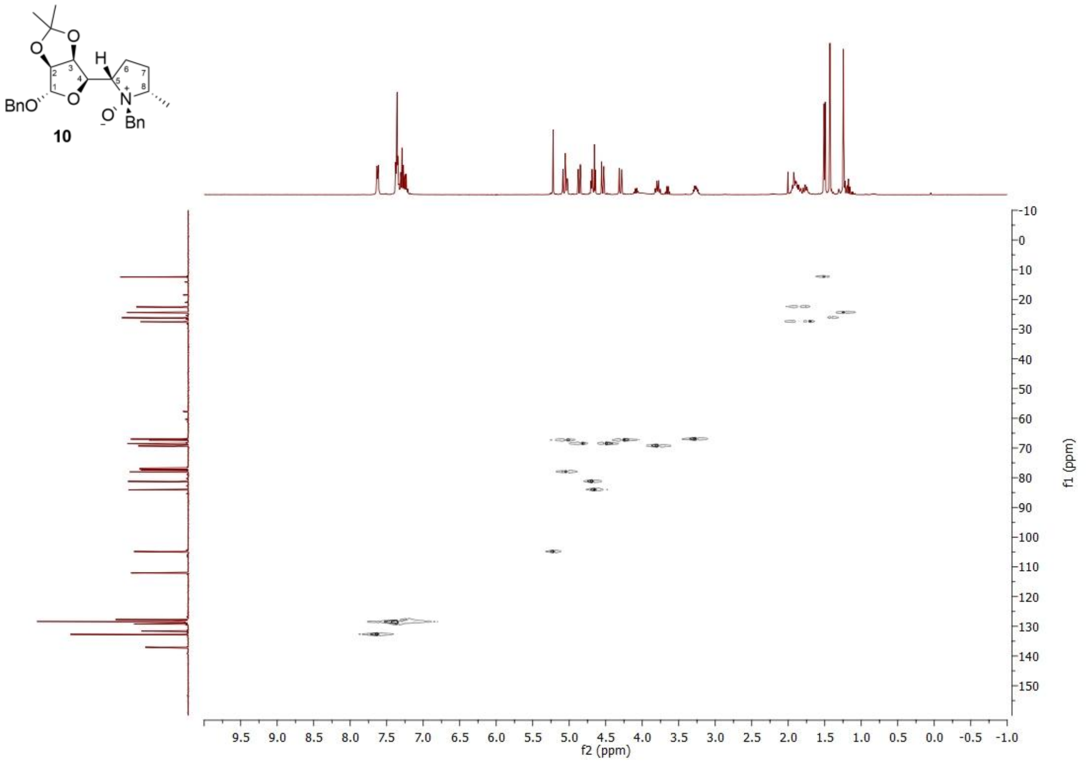

Figure S6. ^{1}H/^{13}C gHSQC (400/100 MHz, $CDCl_3$) of compound **10**.

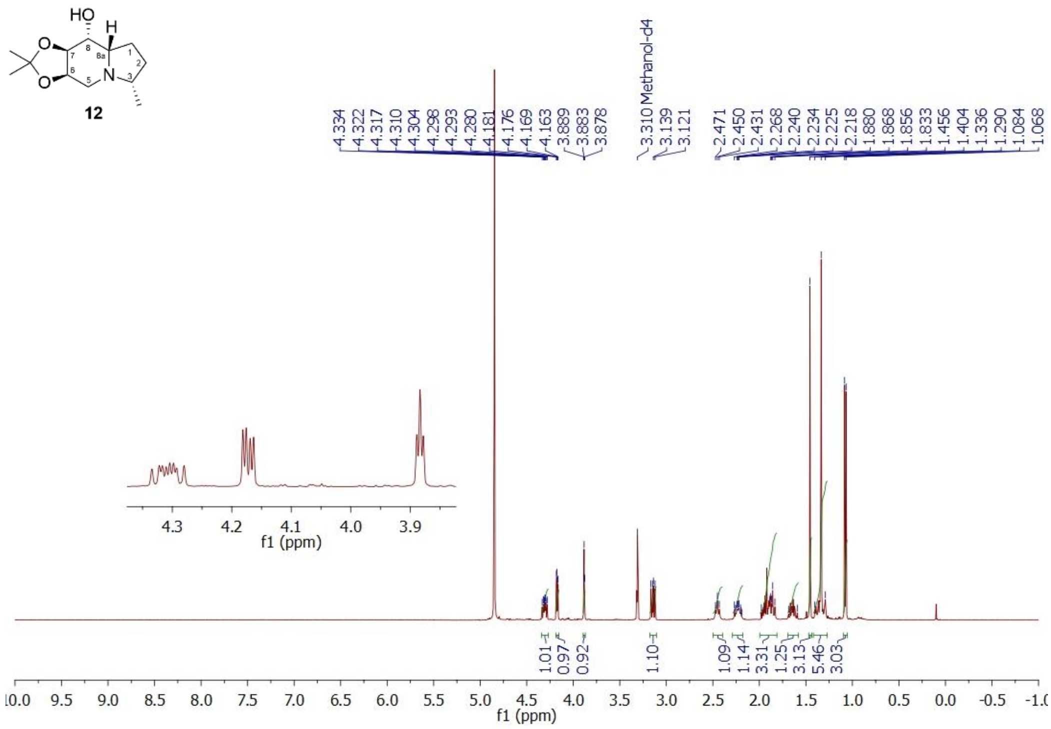

Figure S7. ^{1}H-NMR (400 MHz, CD_3OD) of compound **12**.

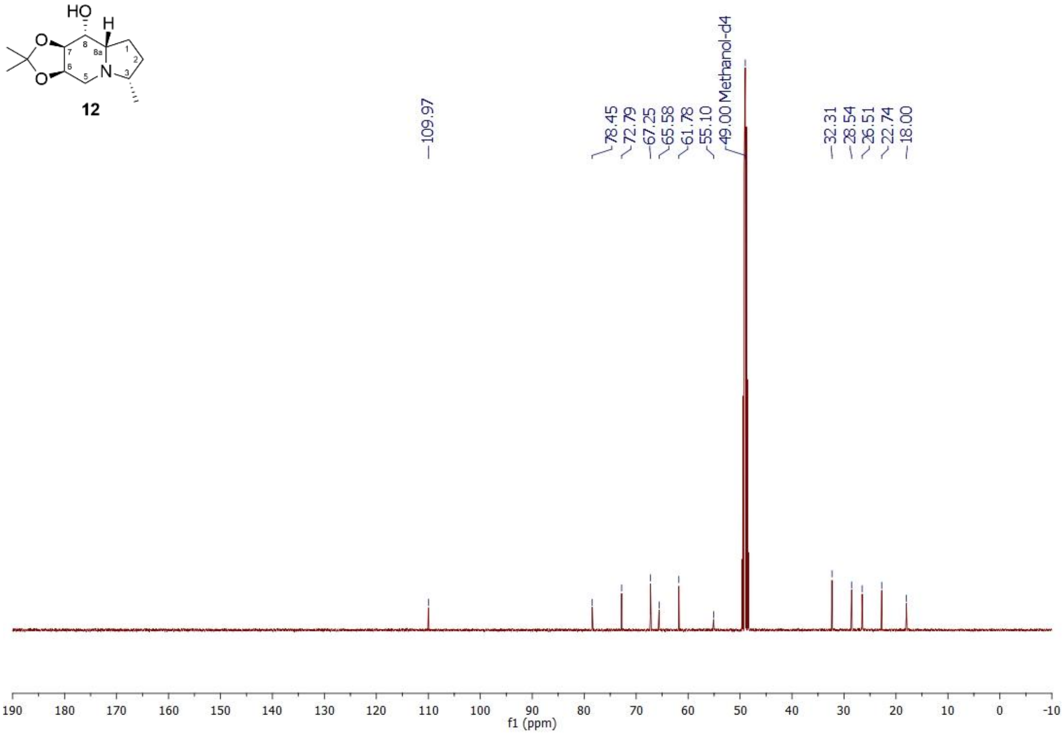

Figure S8. $^{13}C\{^{1}H\}$-NMR (100 MHz, CD_3OD) of compound **12**.

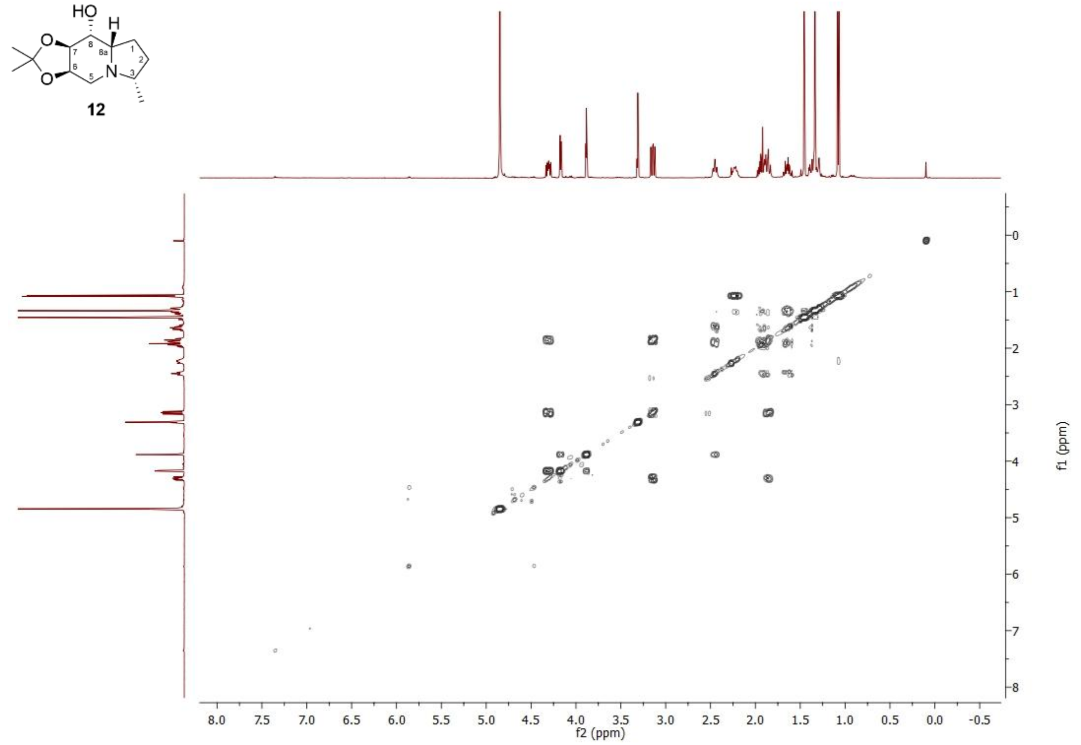

Figure S9. ^{1}H/^{1}H gCOSY (400 MHz, CD_3OD) of compound **12**.

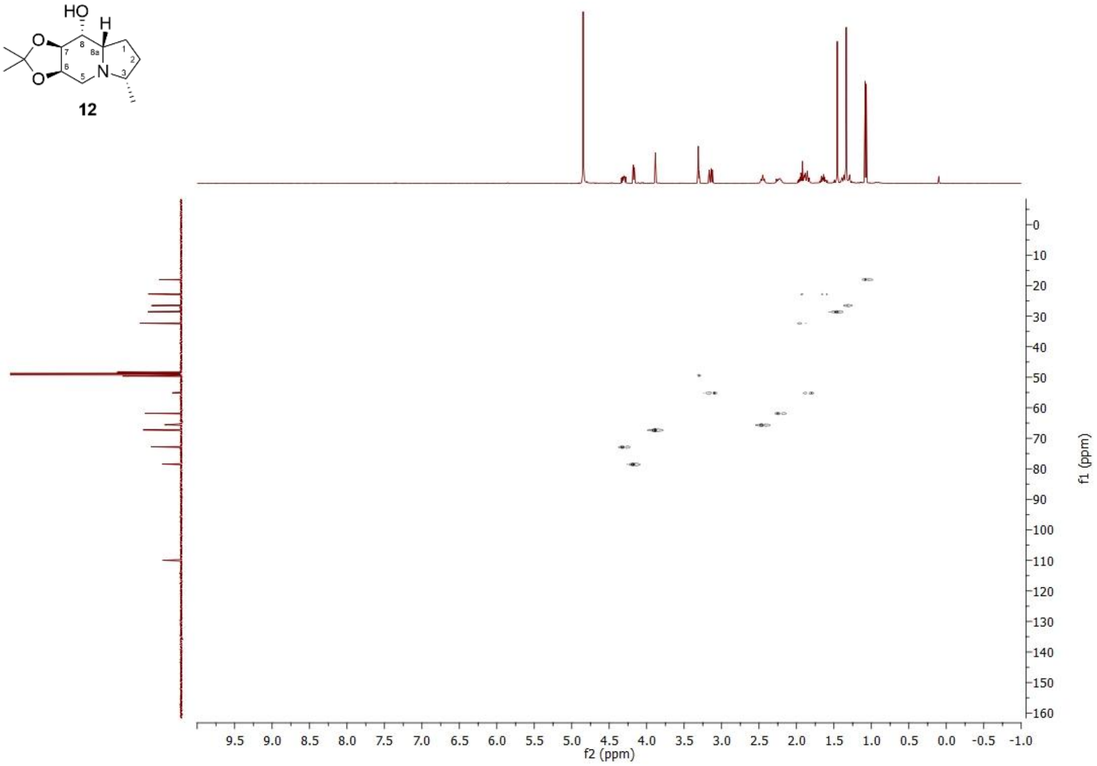

Figure S10. ^{1}H/^{13}C gHSQC (400/100 MHz, CD_3OD) of compound **12**.

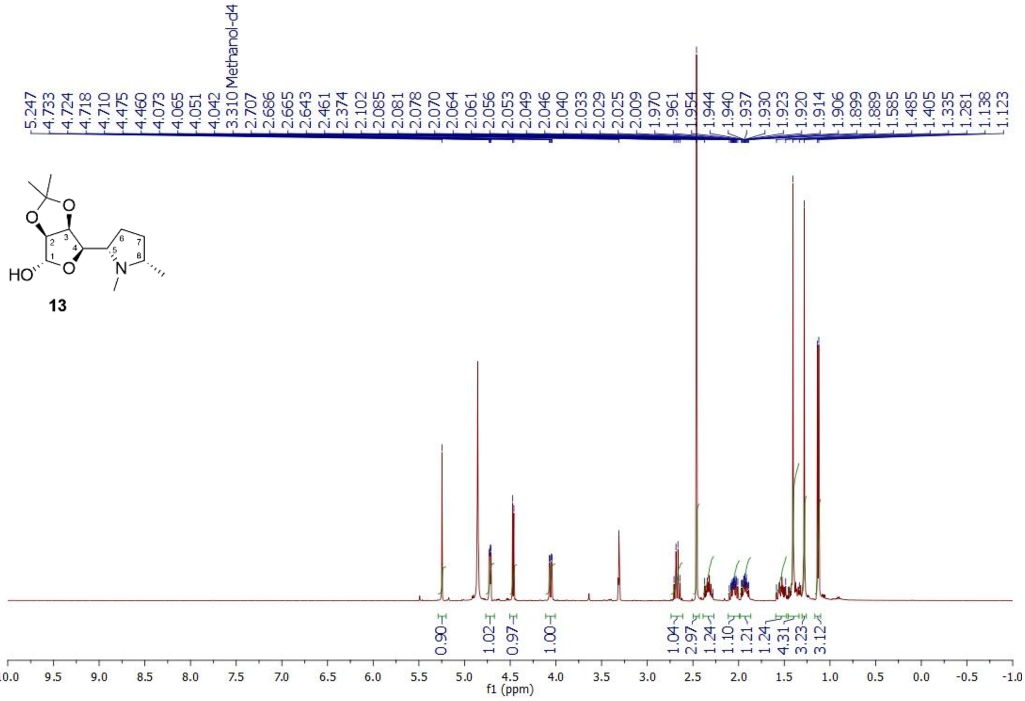

Figure S11. ^{1}H-NMR (400 MHz, CD_3OD) of compound **13**.

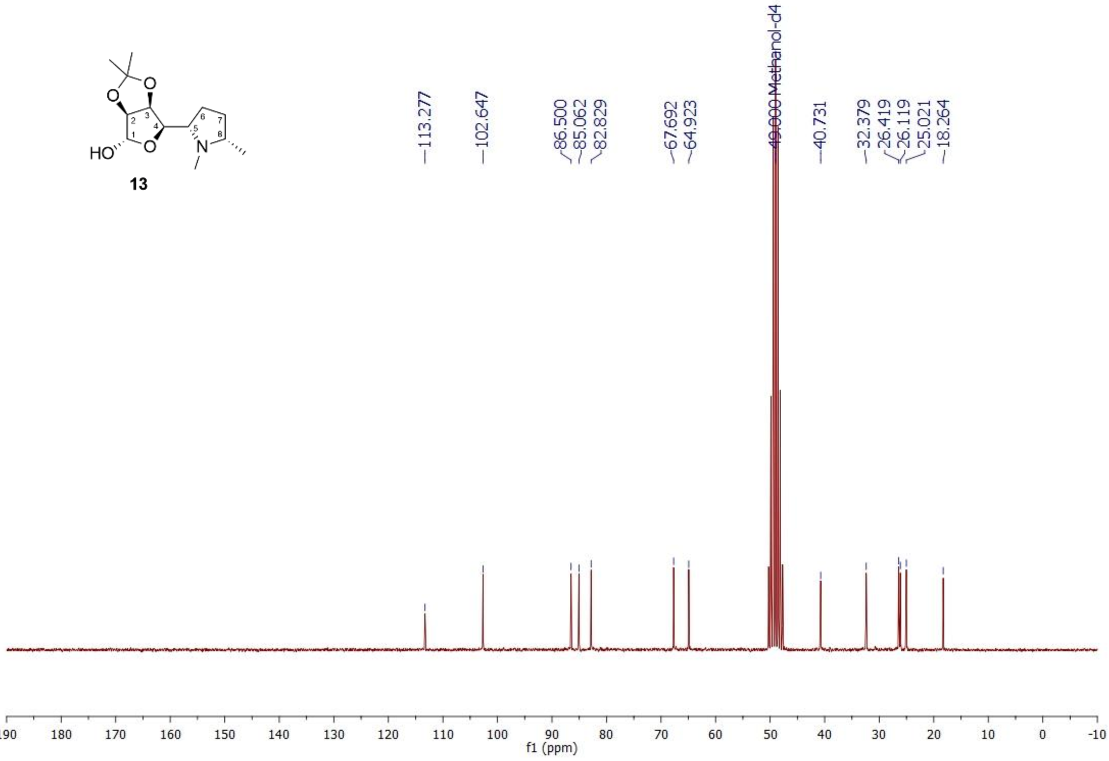

Figure S12. $^{13}C\{^{1}H\}$-NMR (100 MHz, CD_3OD) of compound **13**.

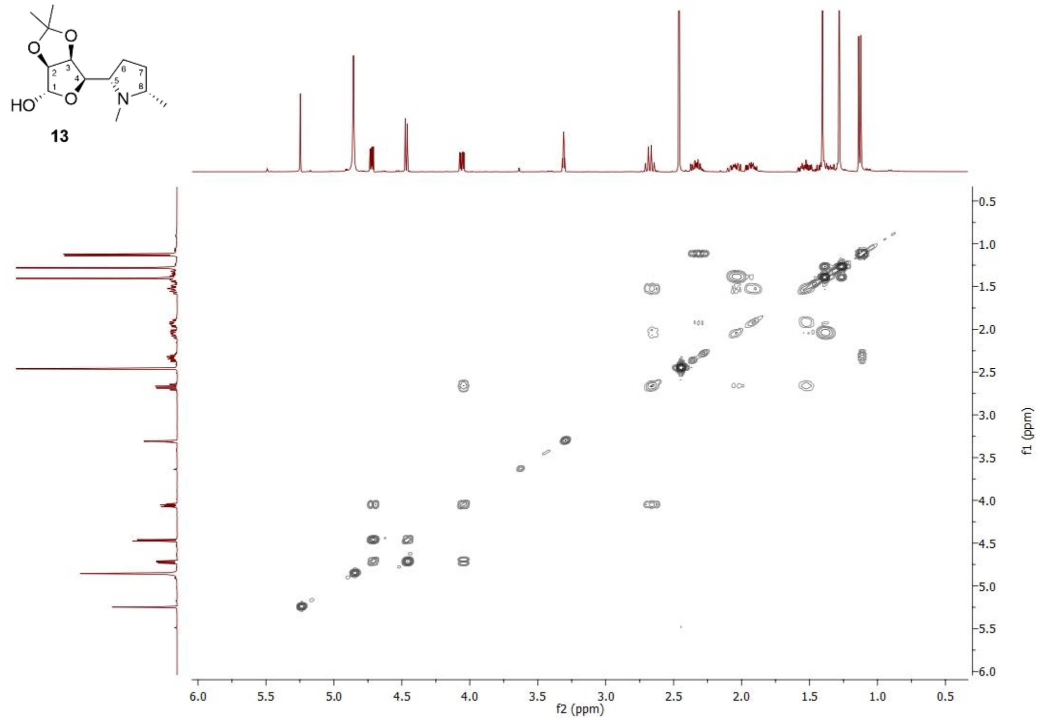

Figure S13. $^{1}H/^{1}H$ gCOSY (400 MHz, CD_3OD) of compound **13**.

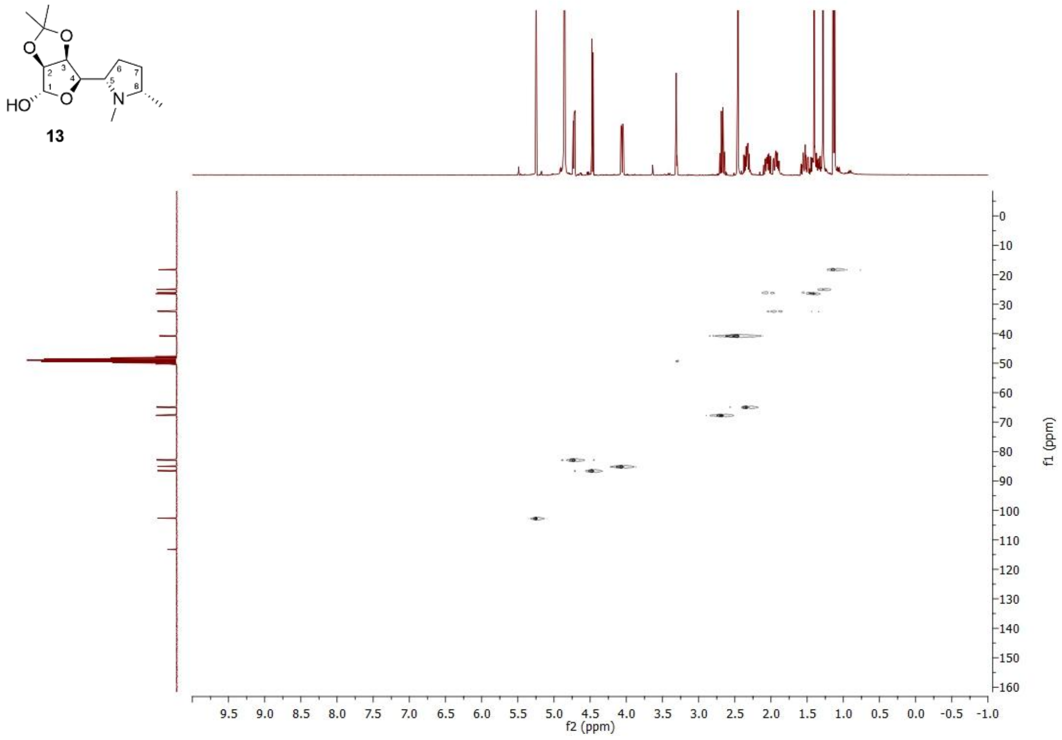

Figure S14. $^{1}H/^{13}C$ gHSQC (400/100 MHz, CD_3OD) of compound **13**.

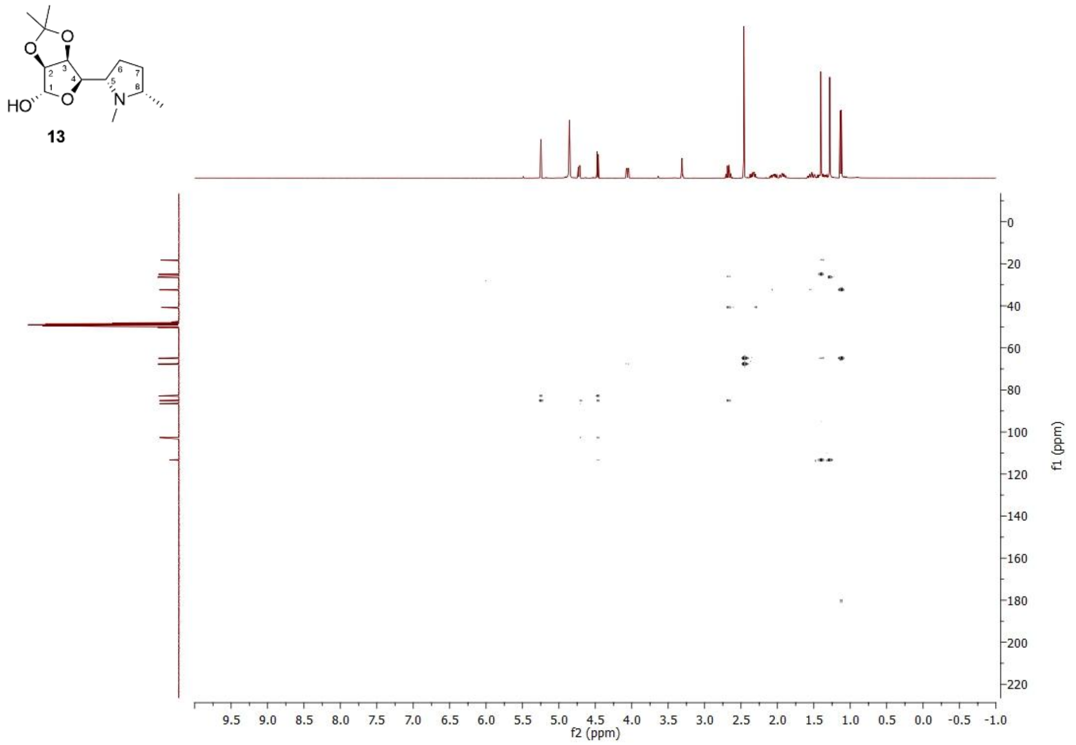

Figure S15. $^{1}H/^{13}C$ gHMBC (400/100 MHz, CD_3OD) of compound **13**.

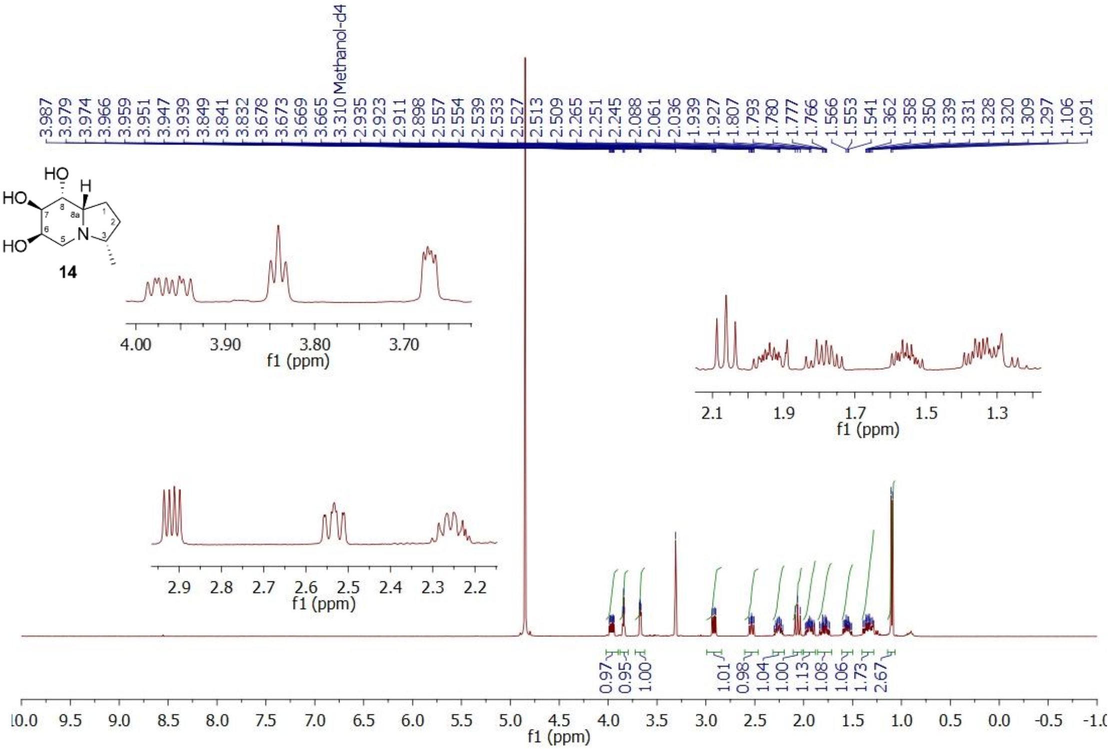

Figure S16. ^{1}H-NMR (400 MHz, CD_3OD) of compound **14**.

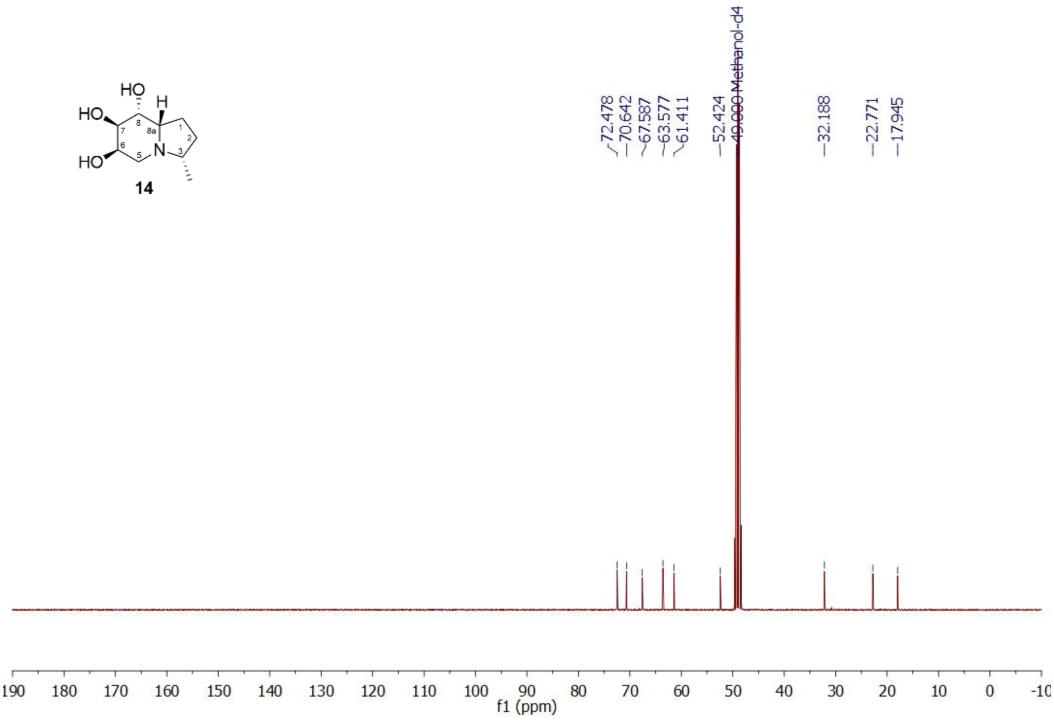

Figure S17. $^{13}C\{^{1}H\}$-NMR (100 MHz, CD_3OD) of compound **14**.

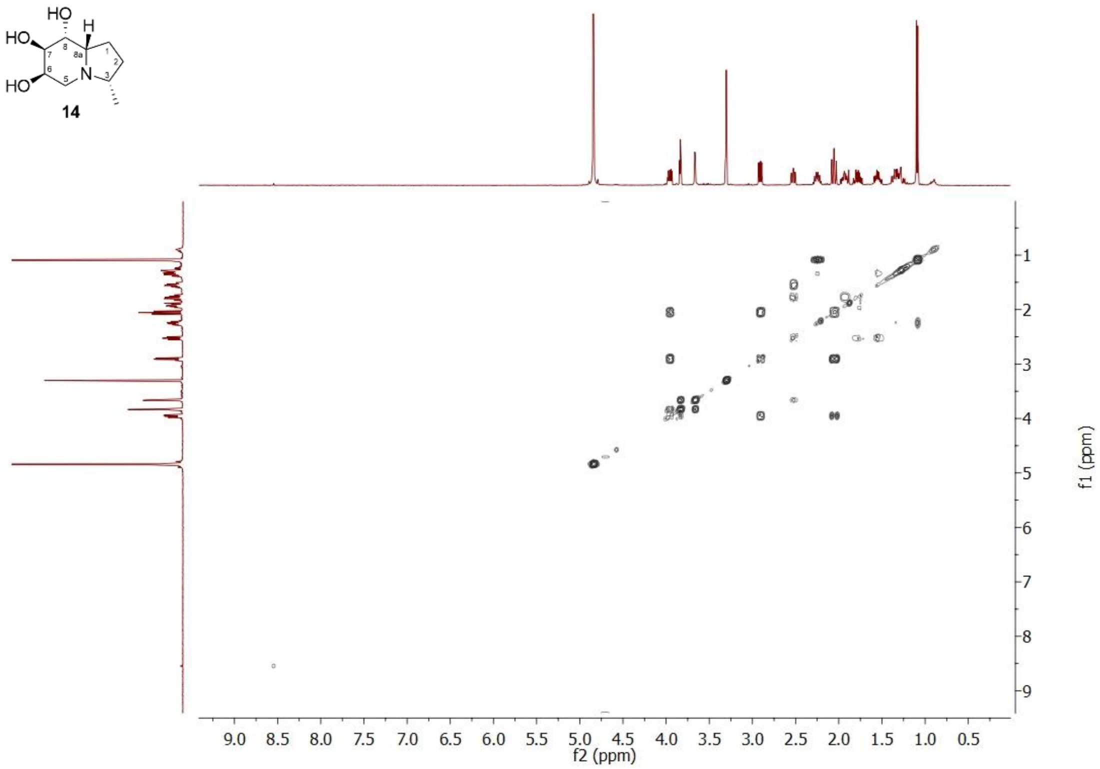

Figure S18. $^{1}H/^{1}H$ gCOSY (400 MHz, CD_3OD) of compound **14**.

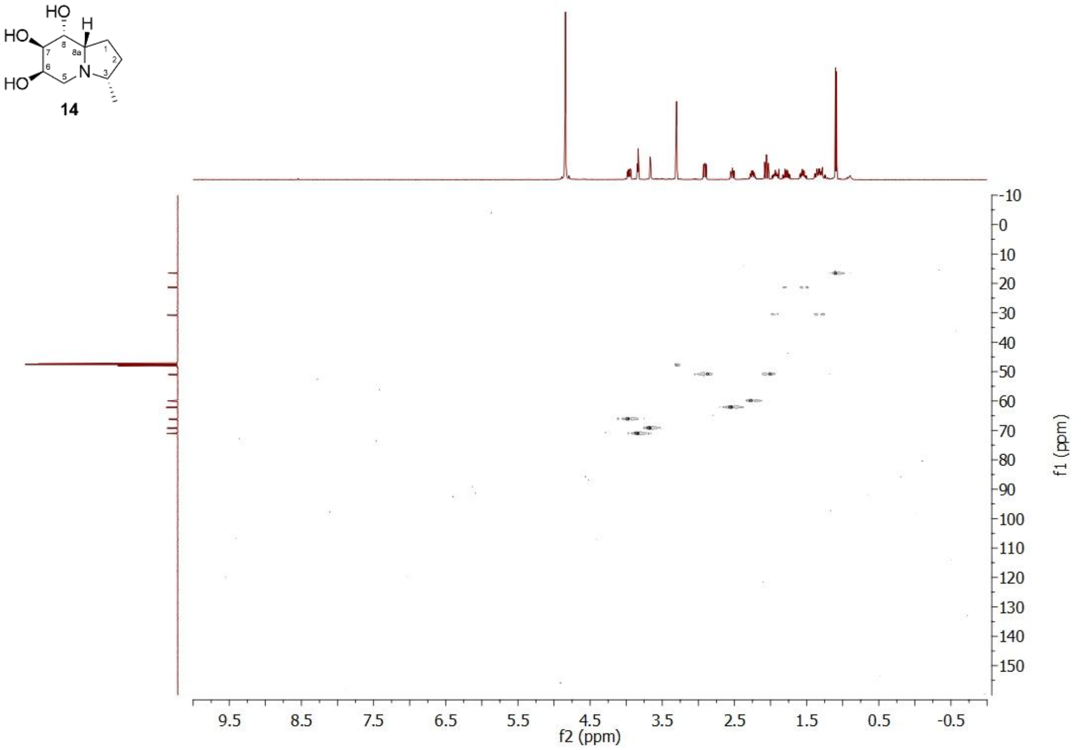

Figure S19. $^{1}H/^{13}C$ gHSQC (400/100 MHz, CD_3OD) of compound **14**.

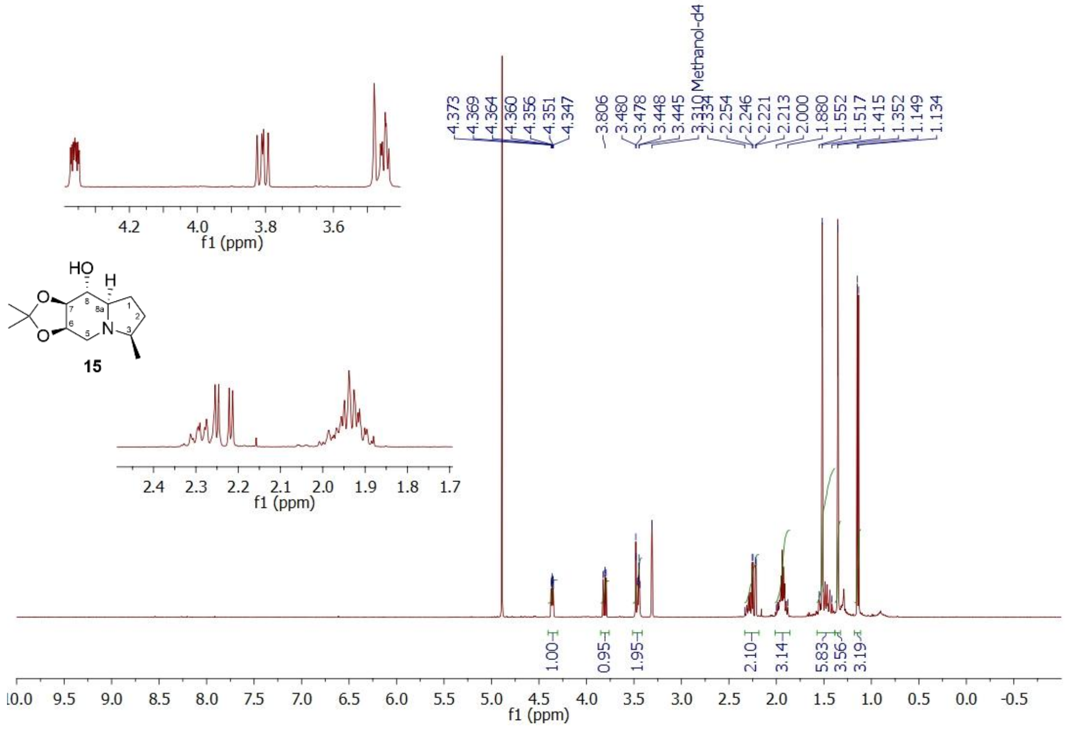

Figure S20. ^{1}H-NMR (400 MHz, CD_3OD) of compound **15**.

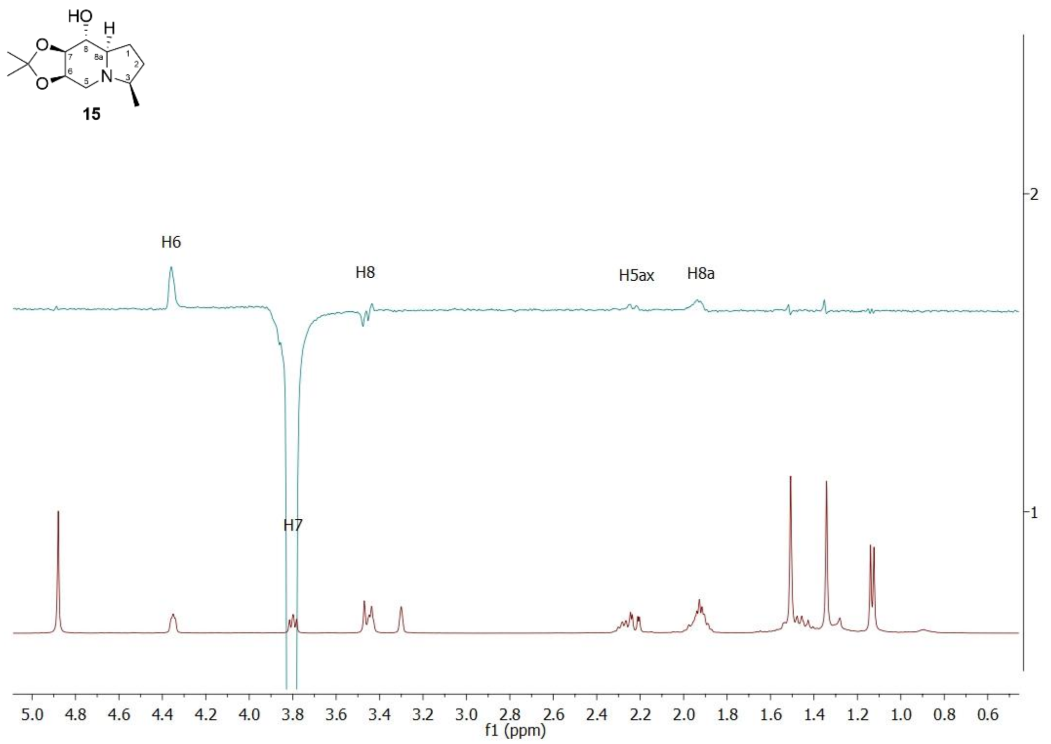

Figure S21. 1D-NOESY spectrum (400 MHz, CD_3OD) of compound **15**; selective irradiation of H-7 (3.81 ppm) gave NOE correlations with H-5_{ax} and H-8a.

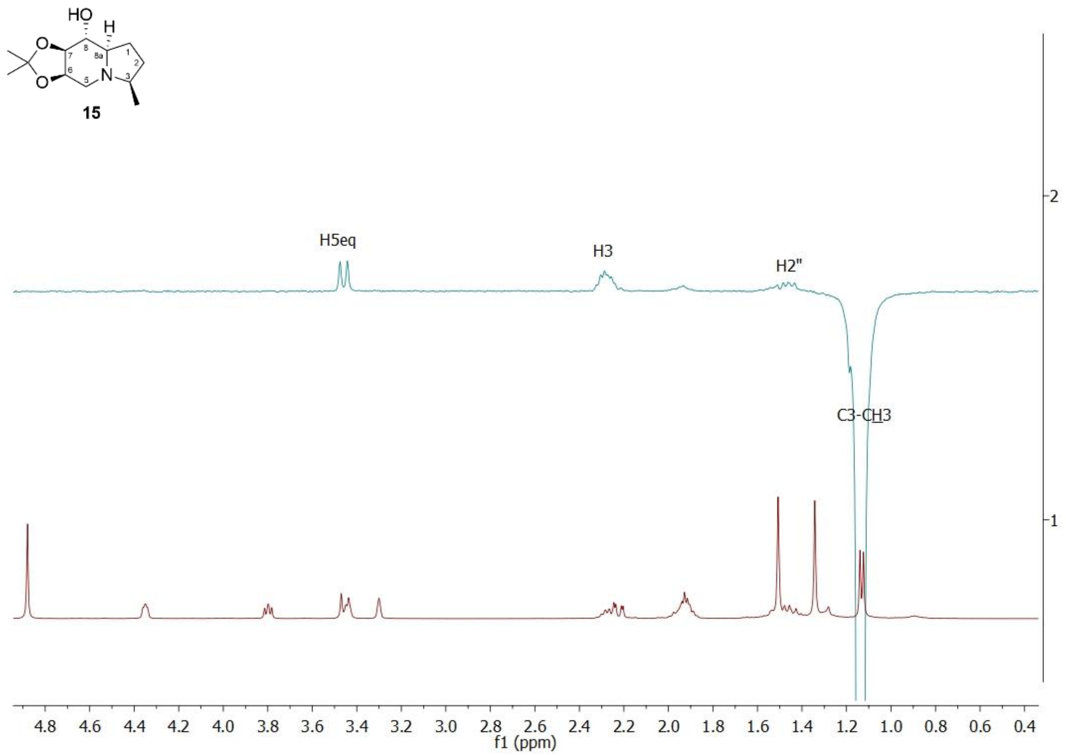

Figure S22. 1D-NOESY spectrum (400 MHz, CD_3OD) of compound **15**; selective irradiation of (C-3)-C$\underline{H}_3$ (1.14 ppm) gave NOE correlation with H-5_{eq}.

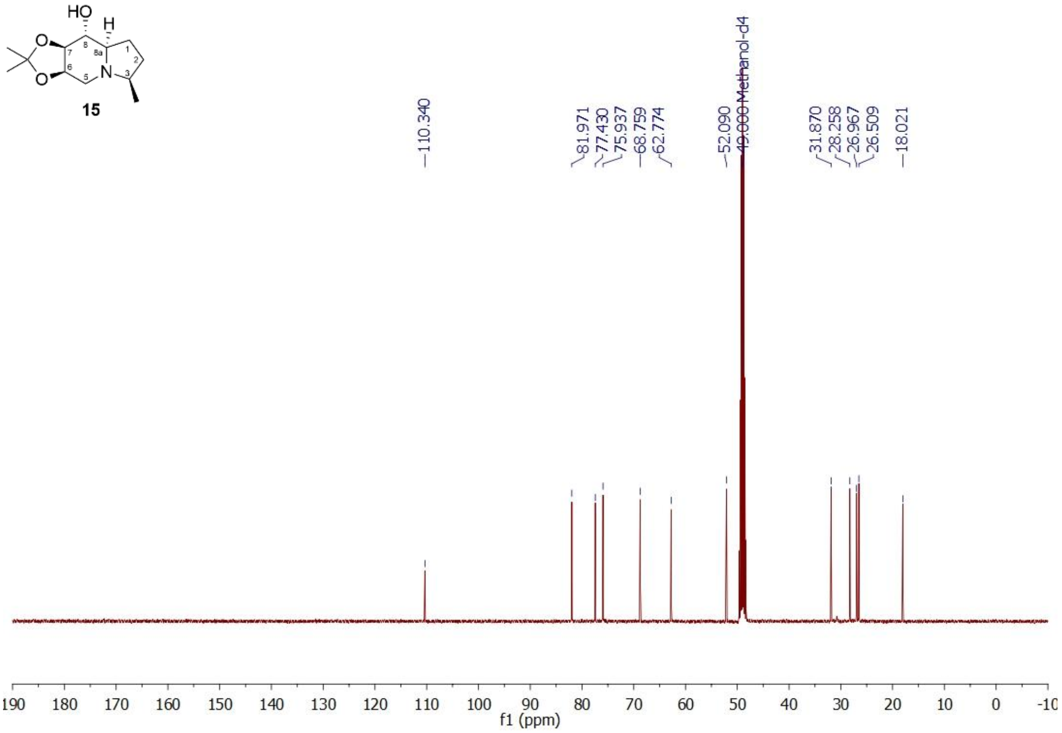

Figure S23. $^{13}C\{^{1}H\}$-NMR (100 MHz, CD_3OD) of compound **15**.

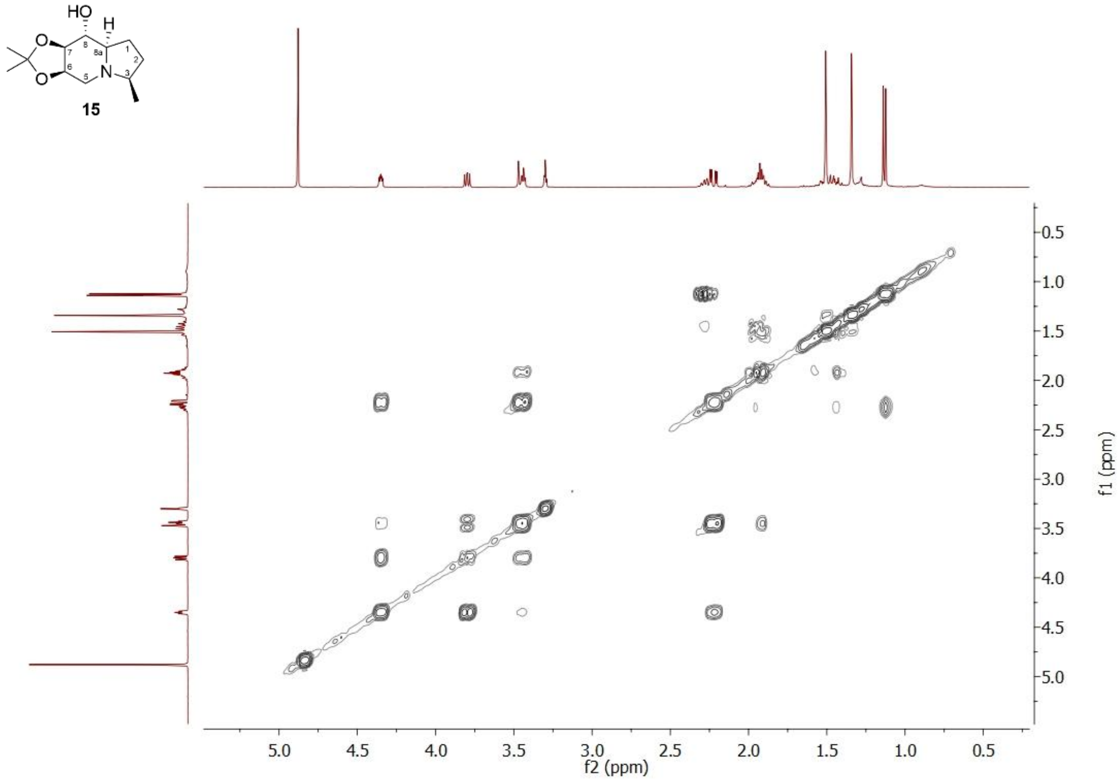

Figure S24. $^{1}H/^{1}H$ gCOSY (400 MHz, CD_3OD) of compound **15**.

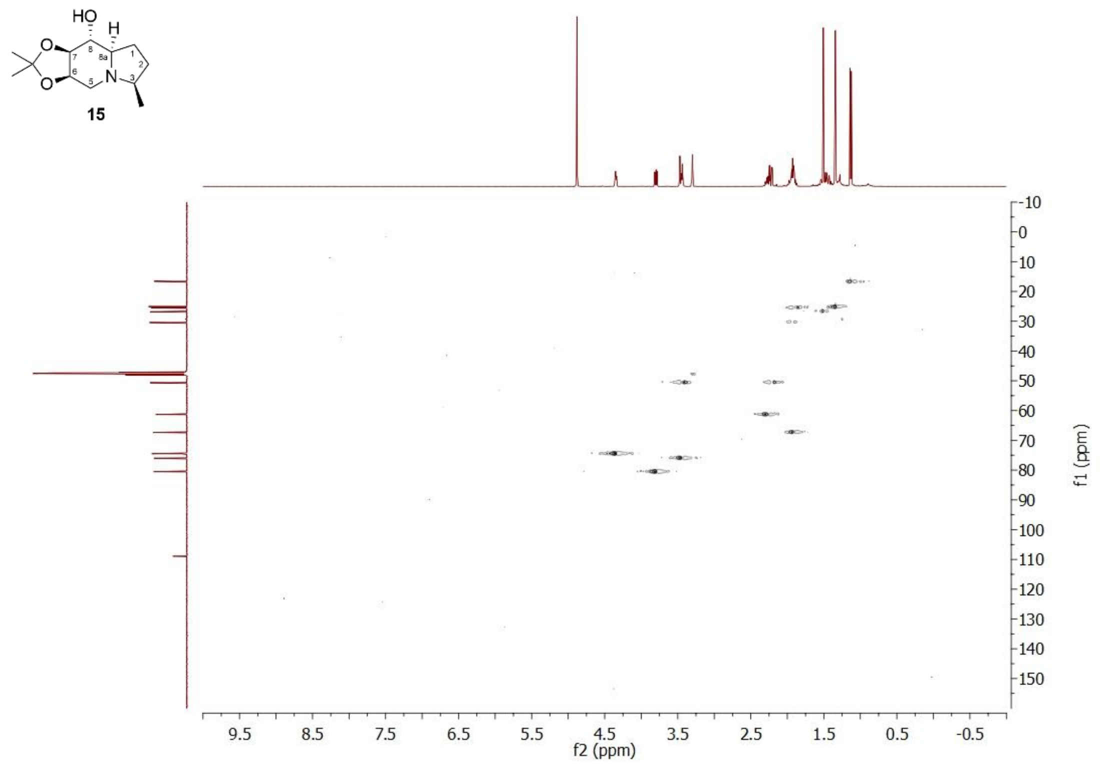

Figure S25. $^{1}H/^{13}C$ gHSQC (400/100 MHz, CD_3OD) of compound **15**.

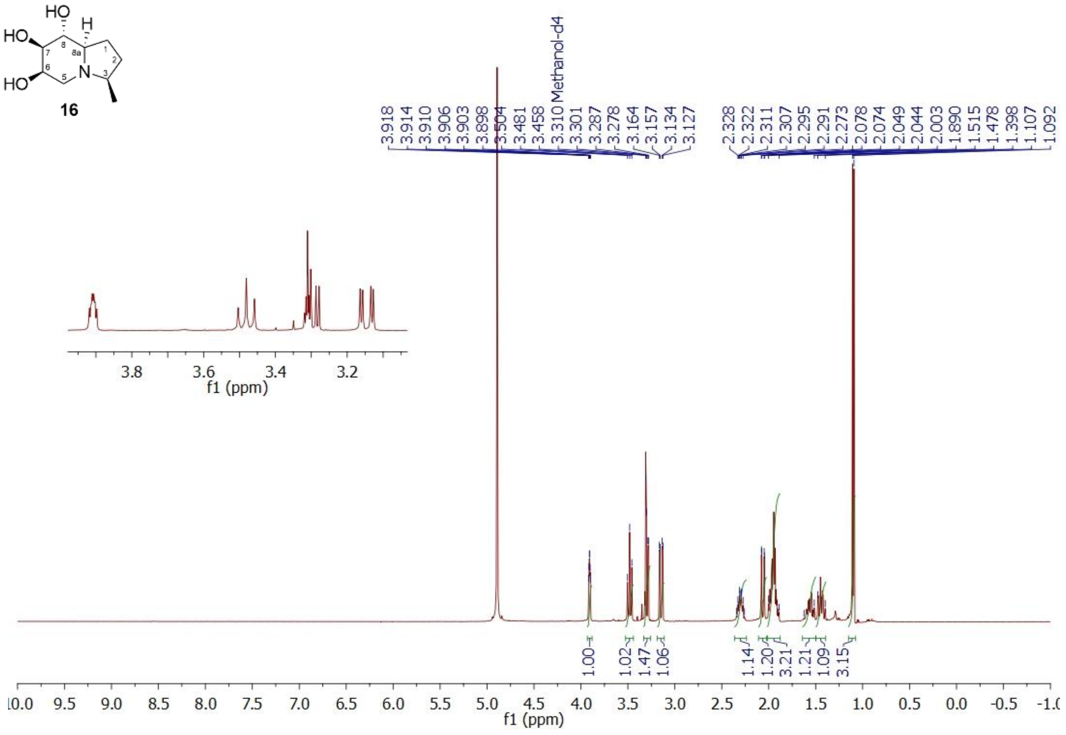

Figure S26. ^{1}H-NMR (400 MHz, CD_3OD) of compound **16**.

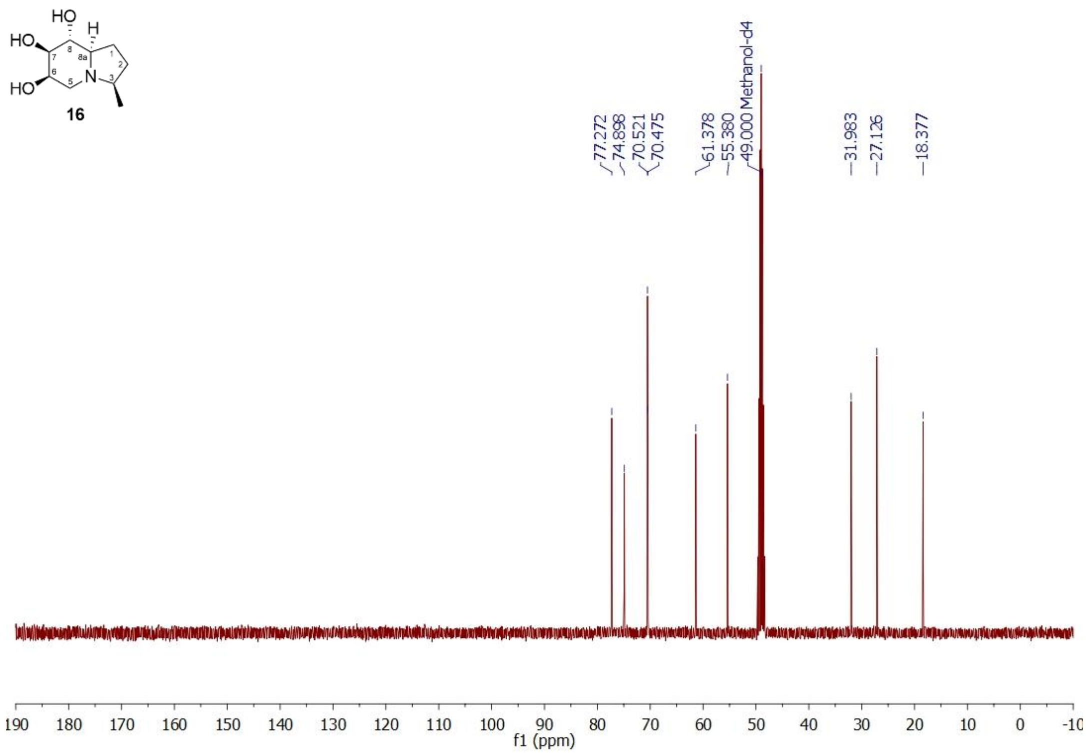

Figure S27. $^{13}C\{^{1}H\}$-NMR (100 MHz, CD_3OD) of compound **16**.

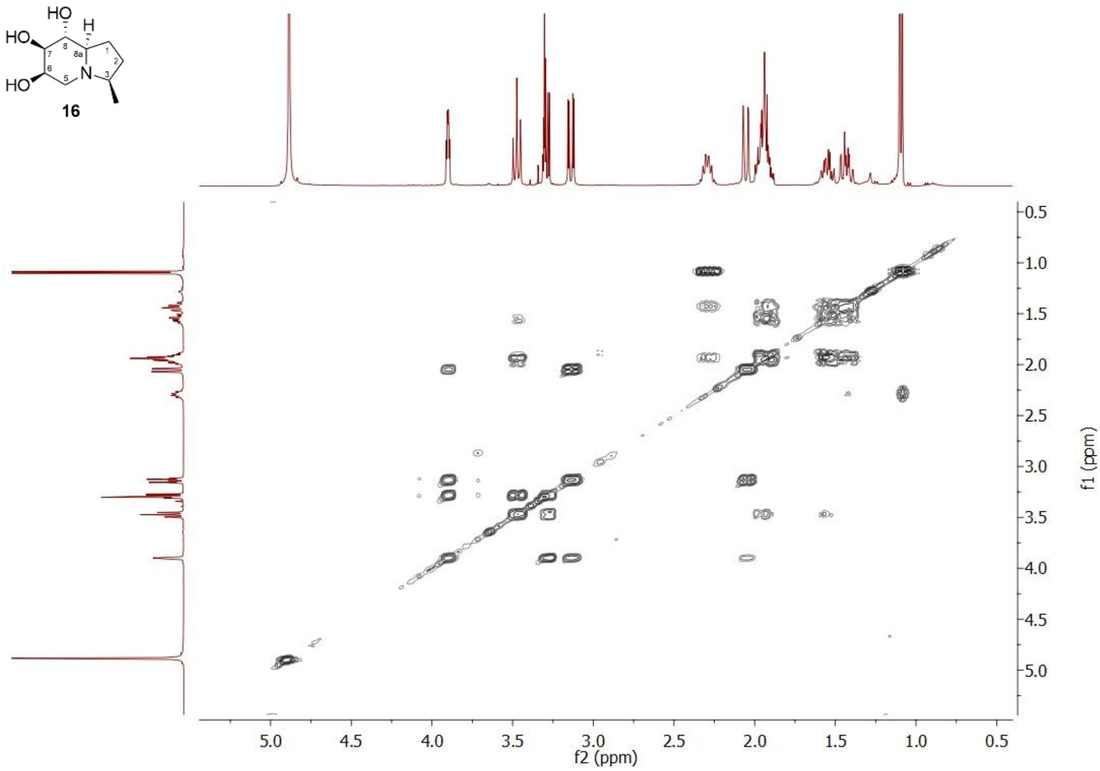

Figure S28. $^{1}H/^{1}H$ gCOSY (400 MHz, CD_3OD) of compound **16**.

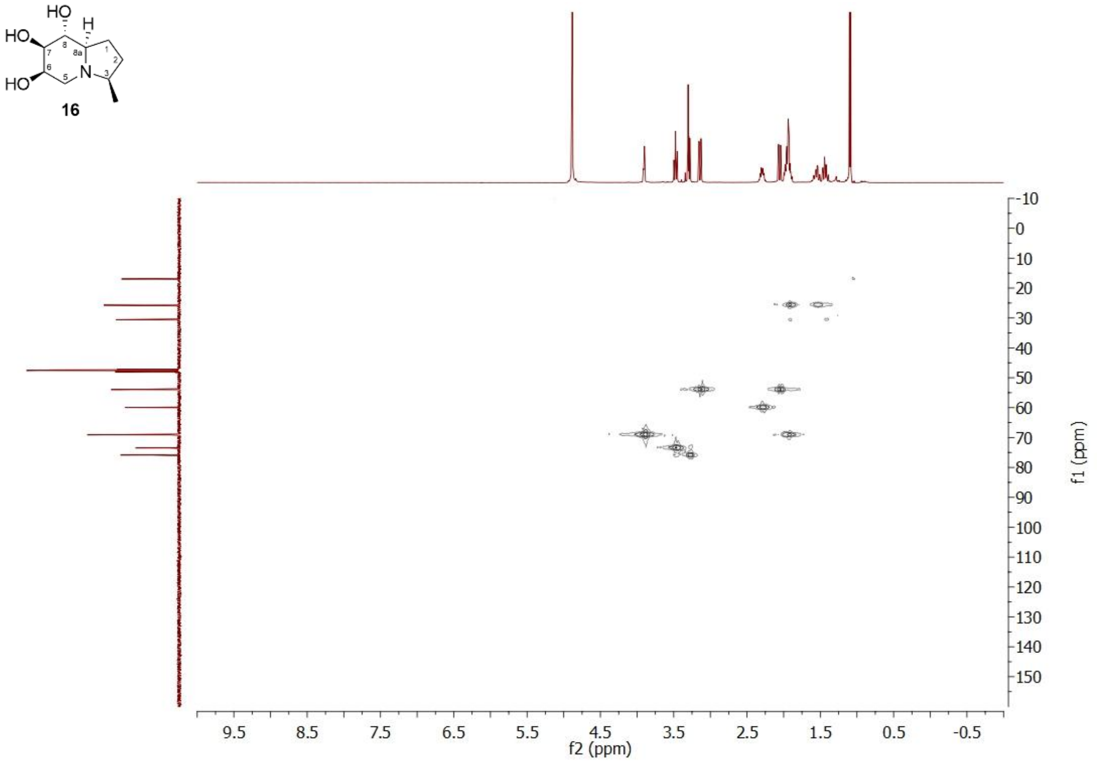

Figure S29. $^{1}H/^{13}C$ gHSQC (400/100 MHz, CD_3OD) of compound **16**.

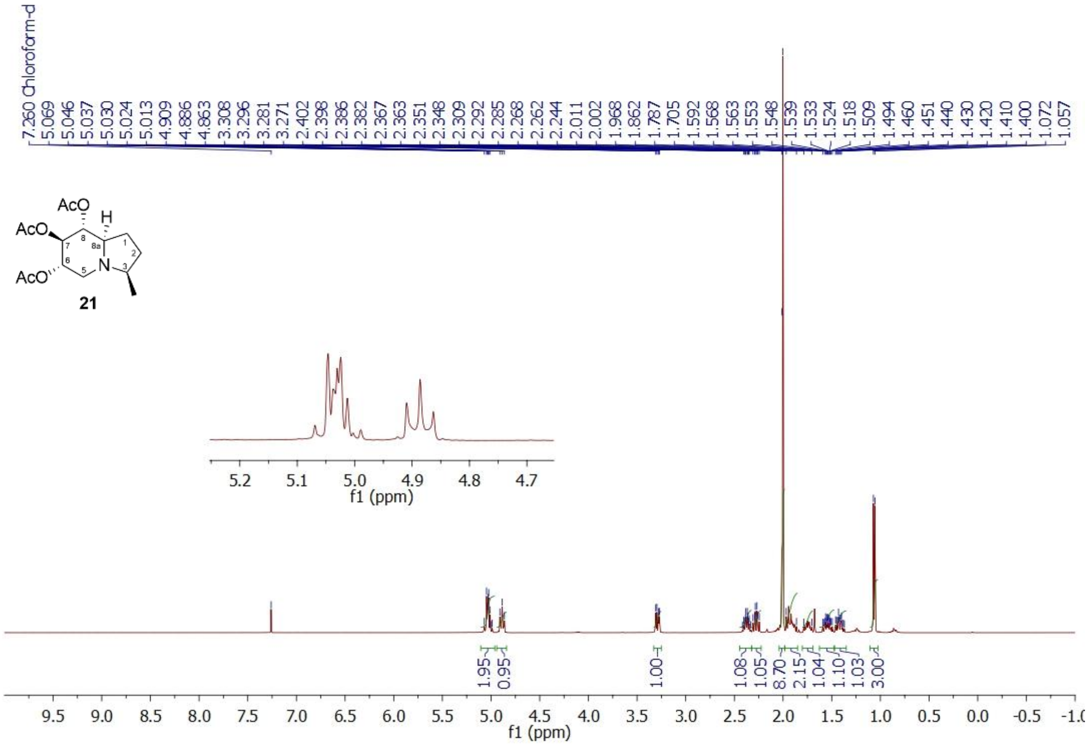

Figure S30. ^{1}H-NMR (400 MHz, $CDCl_3$) of compound **21**.

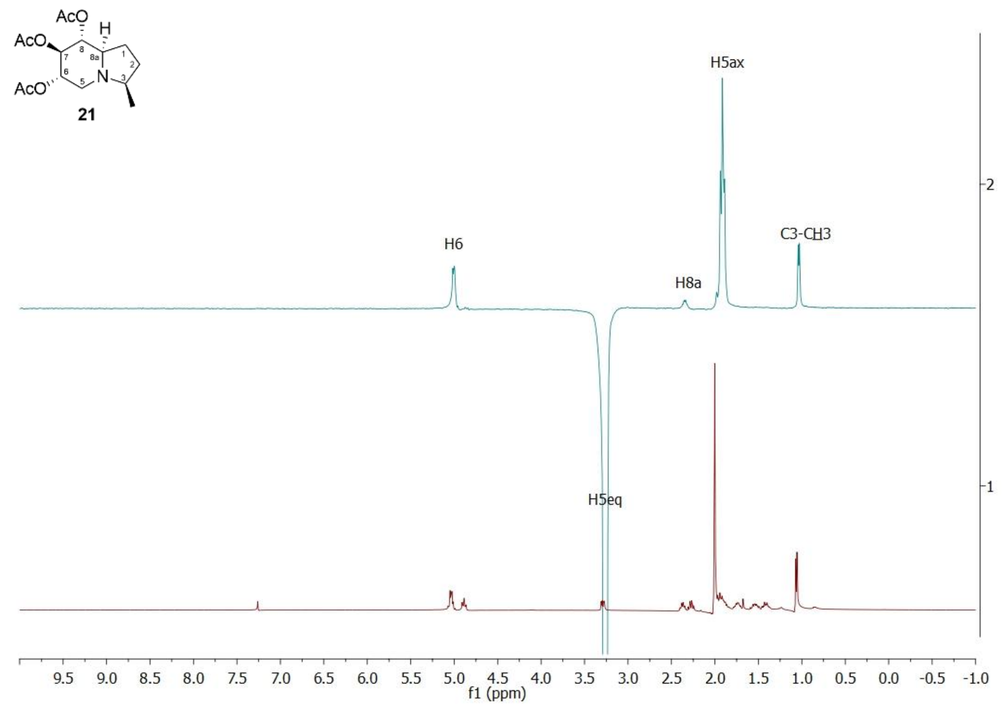

Figure S31. 1D-NOESY spectrum (400 MHz, $CDCl_3$) of compound **21**; selective irradiation of H-5_{eq} (3.29 ppm) gave NOE correlations with H-8a and (C-3)-C$\underline{H}_3$.

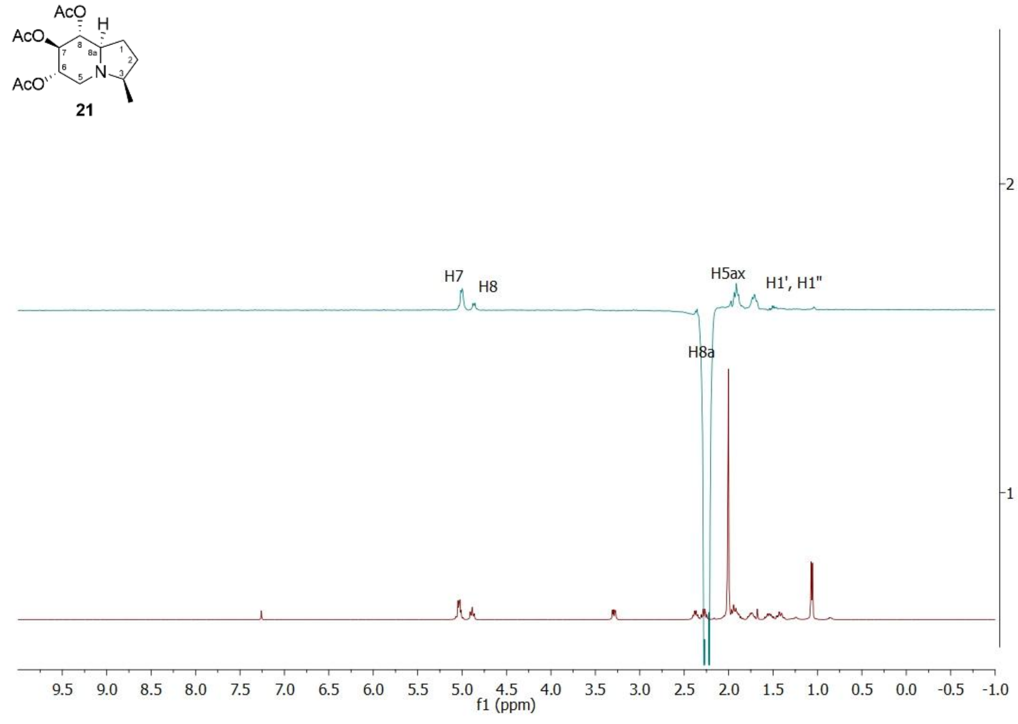

Figure S32. 1D-NOESY spectrum (400 MHz, $CDCl_3$) of compound **21**; selective irradiation of H-8a (2.28 ppm) gave NOE correlations with H-5_{ax} and H-7.

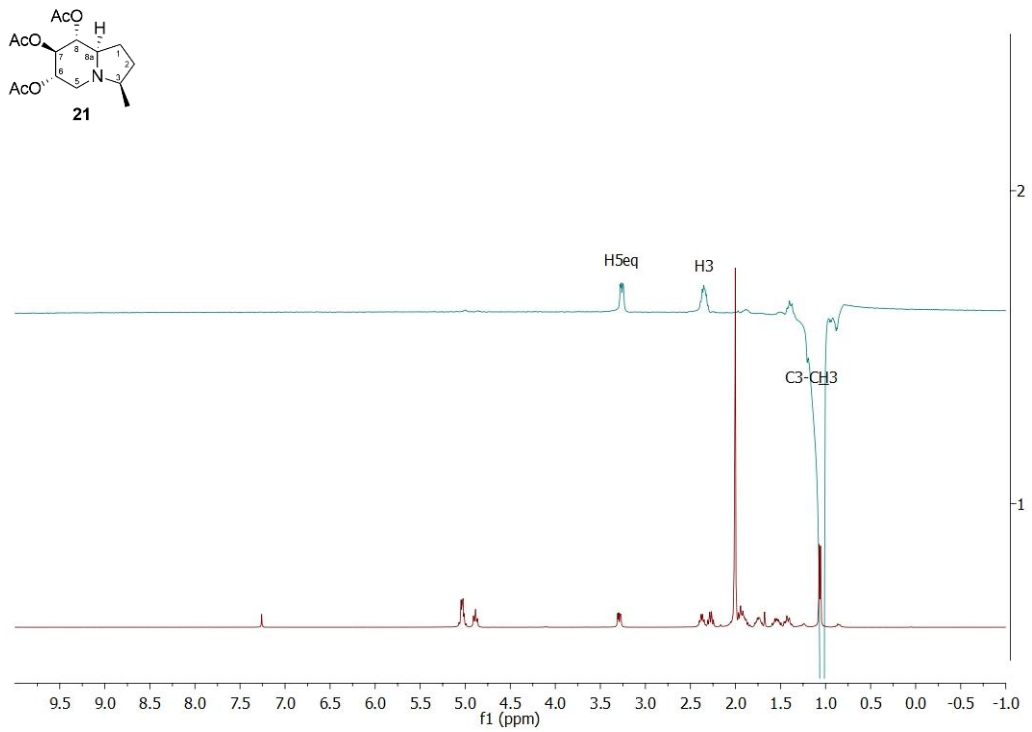

Figure S33. 1D-NOESY spectrum (400 MHz, $CDCl_3$) of compound **21**; selective irradiation of (C-3)-C<u>H</u>$_3$ (1.06 ppm) gave NOE correlation with H-5$_{eq}$.

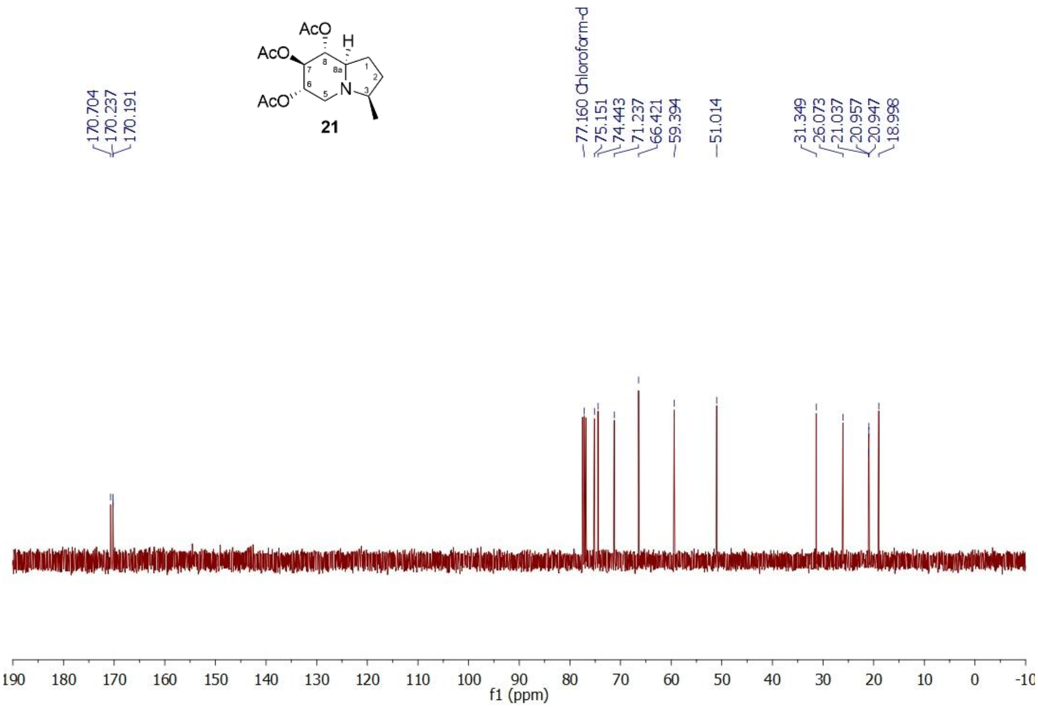

Figure S34. $^{13}C\{^1H\}$-NMR (100 MHz, $CDCl_3$) of compound **21**.

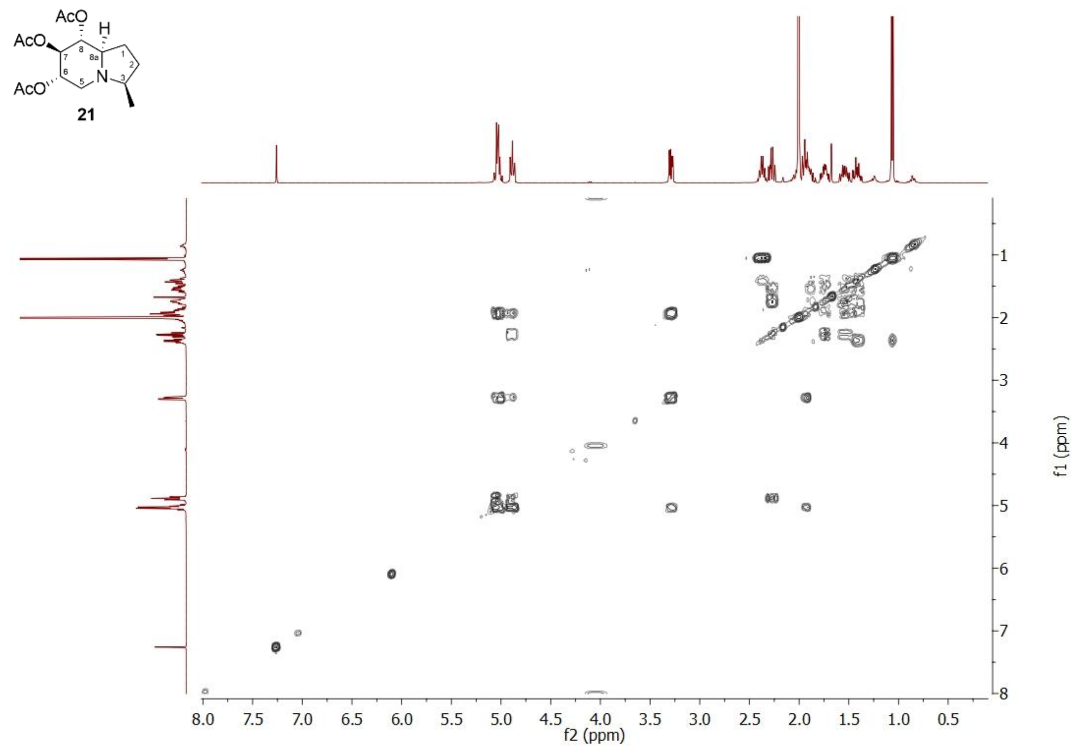

Figure S35. ^{1}H/^{1}H gCOSY (400 MHz, $CDCl_3$) of compound **21**.

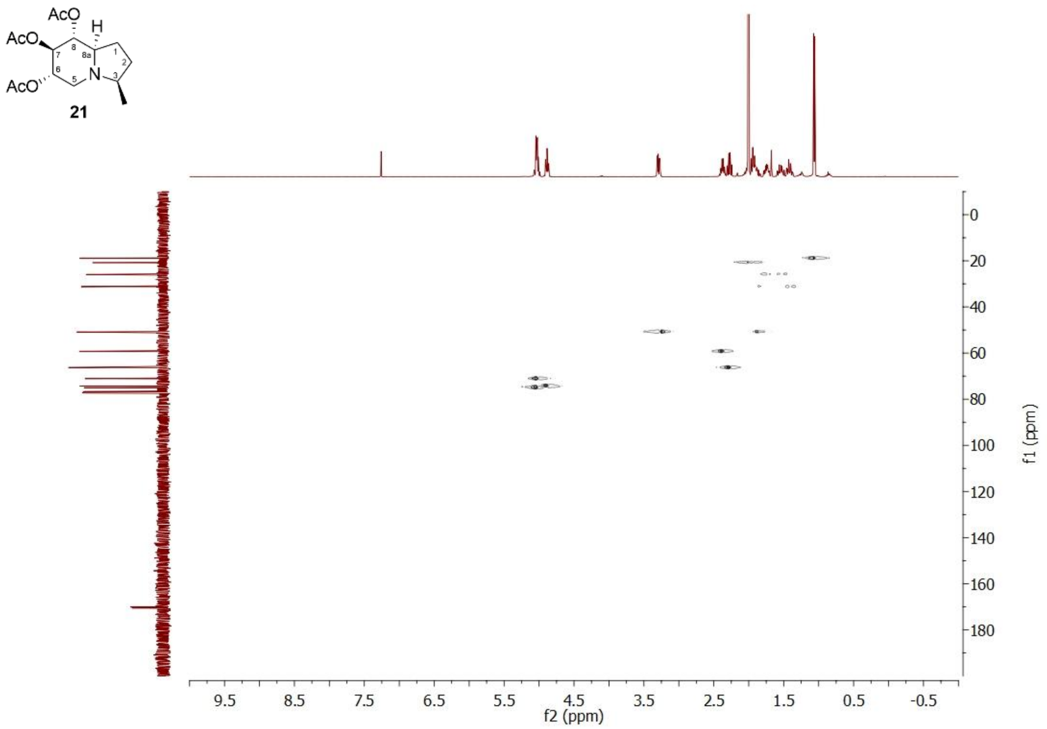

Figure S36. ^{1}H/^{13}C gHSQC (400/100 MHz, $CDCl_3$) of compound **21**.

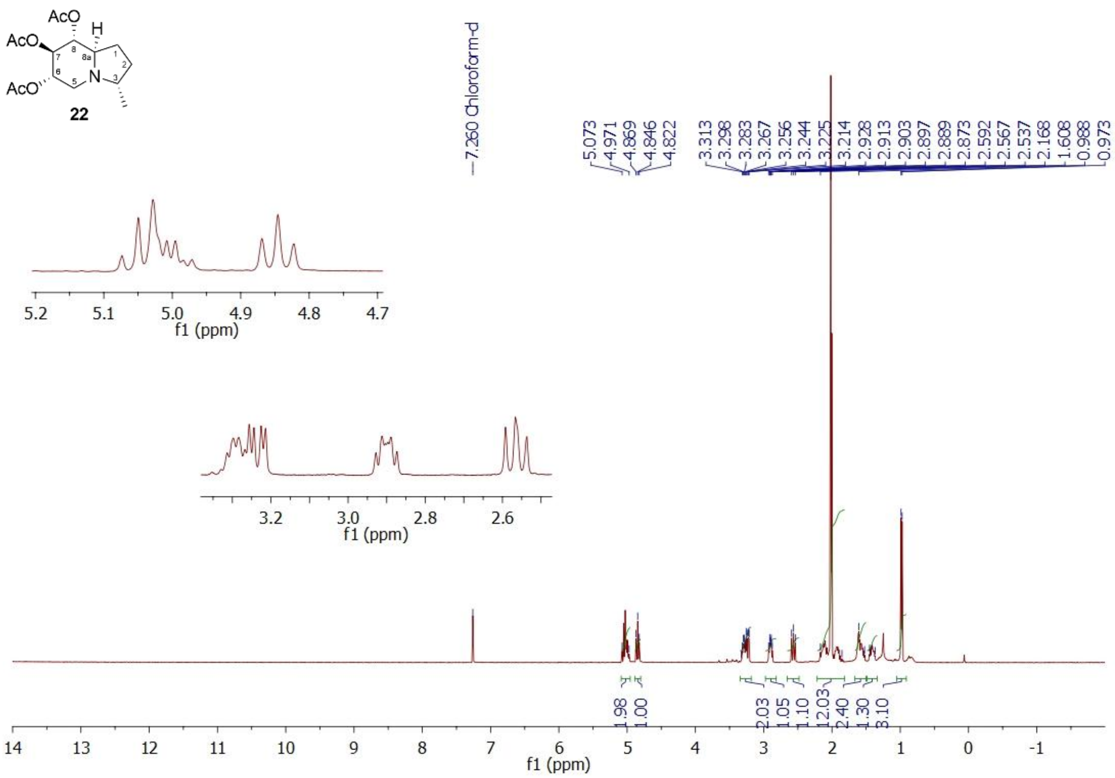

Figure S37. ^{1}H-NMR (400 MHz, $CDCl_3$) of compound **22**.

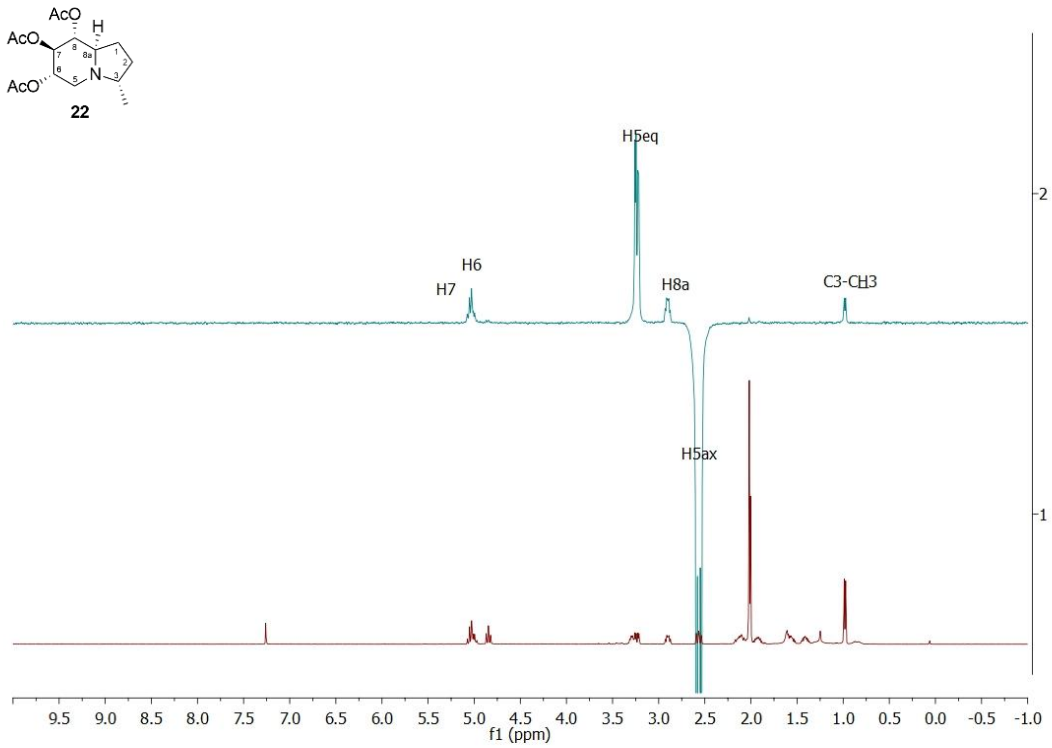

Figure S38. 1D-NOESY spectrum (400 MHz, $CDCl_3$) of compound **22**; selective irradiation of H-5_{ax} (2.56 ppm) gave NOE correlations with H-7, H-8a and (C-3)-C$\underline{H}_3$.

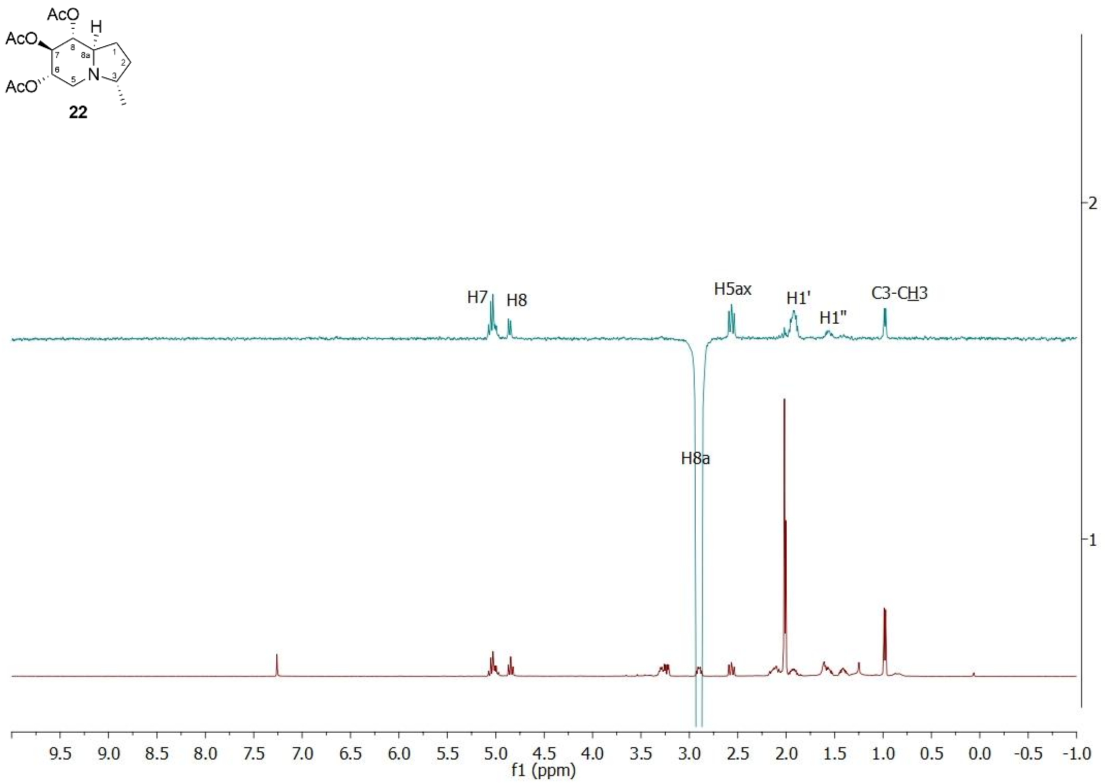

Figure S39. 1D-NOESY spectrum (400 MHz, $CDCl_3$) of compound **22**; selective irradiation of H-8a (2.90 ppm) gave NOE correlations with H-5_{ax}, H-7 and (C-3)-C$\underline{H}_3$.

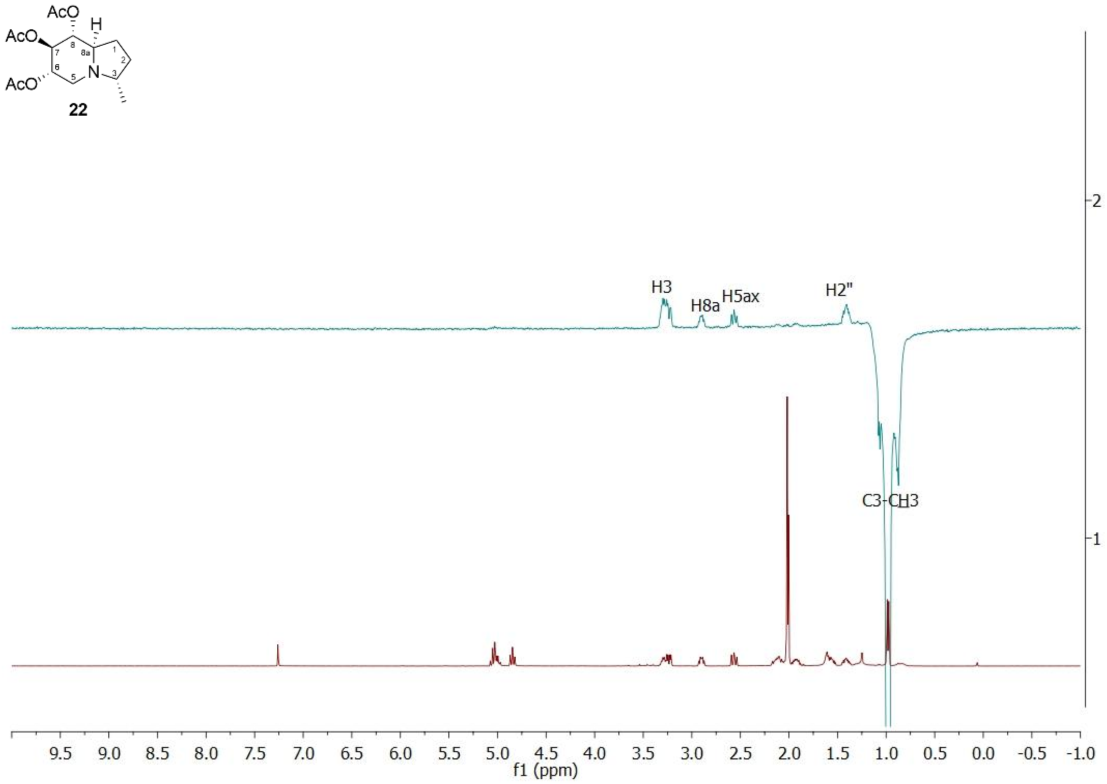

Figure S40. 1D-NOESY spectrum (400 MHz, $CDCl_3$) of compound **22**; selective irradiation of (C-3)-C$\underline{H}_3$ (0.98 ppm) gave NOE correlations with H-5_{ax}, H-8a and H-2''.

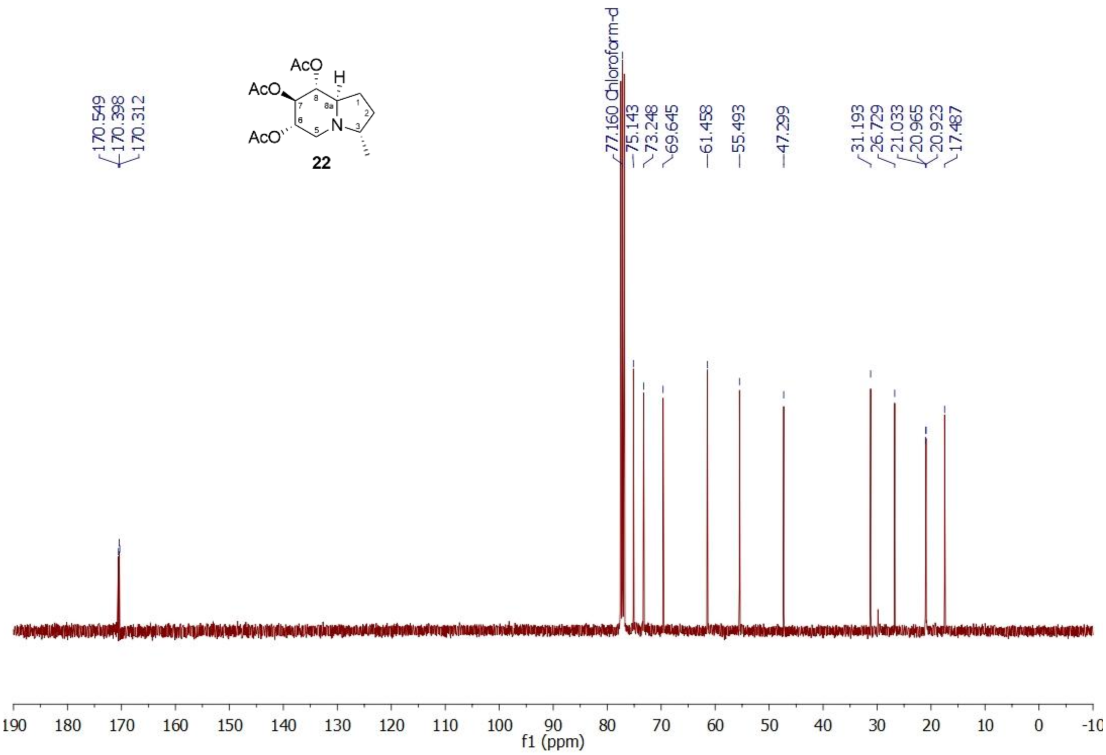

Figure S41. $^{13}C\{^{1}H\}$-NMR (100 MHz, $CDCl_3$) of compound **22**.

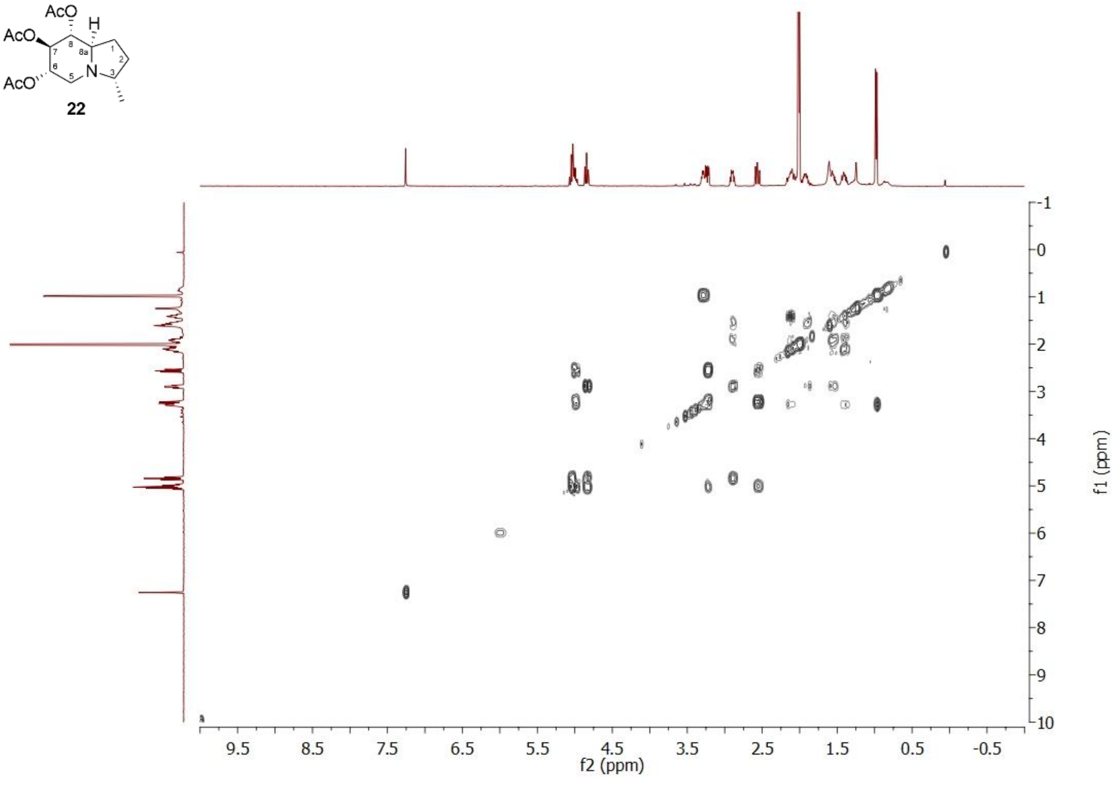

Figure S42. $^{1}H/^{1}H$ gCOSY (400 MHz, $CDCl_3$) of compound **22**.

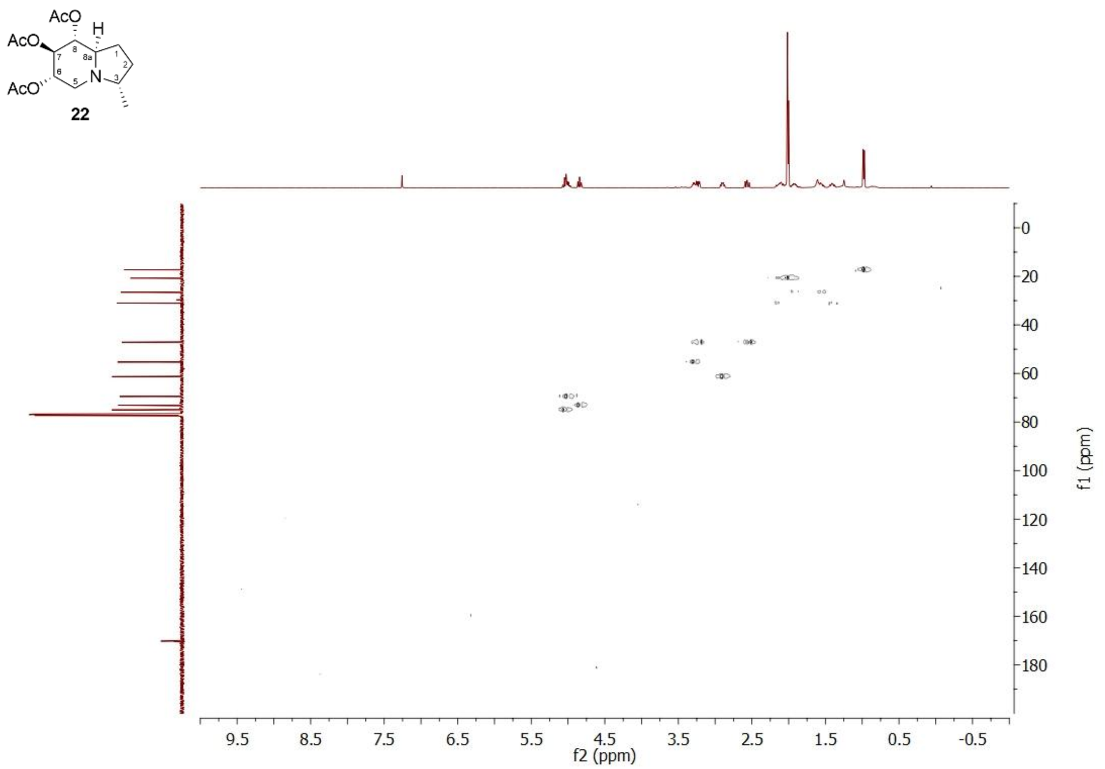

Figure S43. $^1H/^{13}C$ gHSQC (400/100 MHz, $CDCl_3$) of compound **22**.

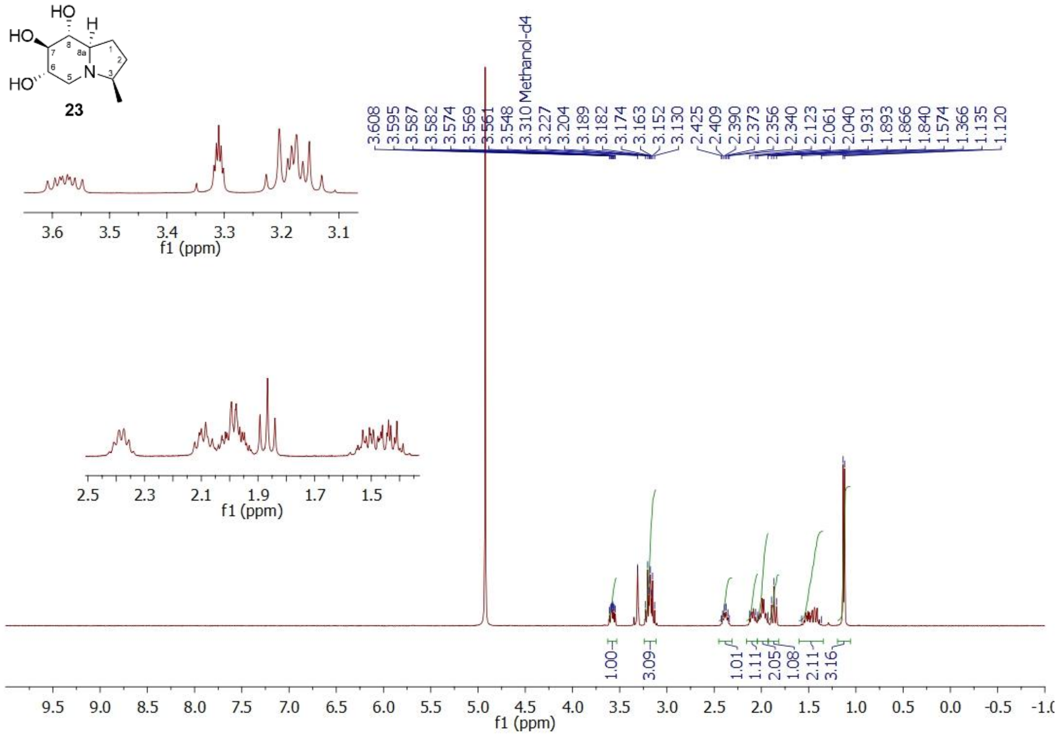

Figure S44. ^1H-NMR (400 MHz, CD_3OD) of compound **23**.

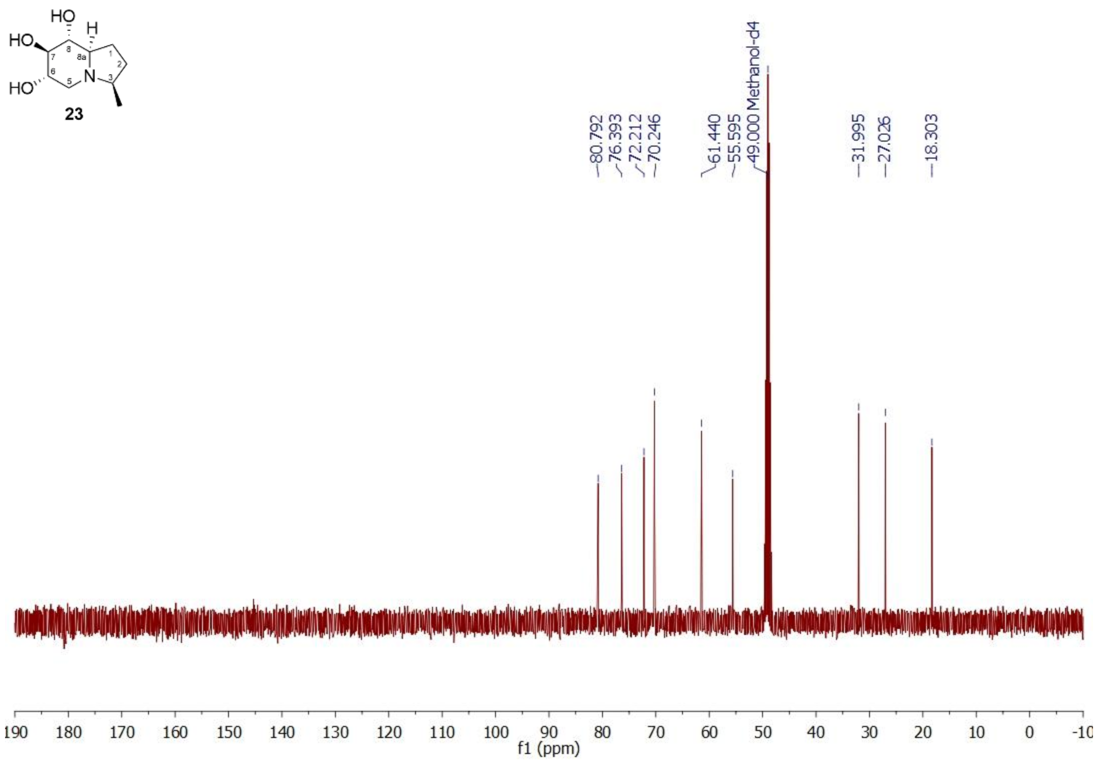

Figure S45. $^{13}C\{^{1}H\}$-NMR (100 MHz, CD_3OD) of compound **23**.

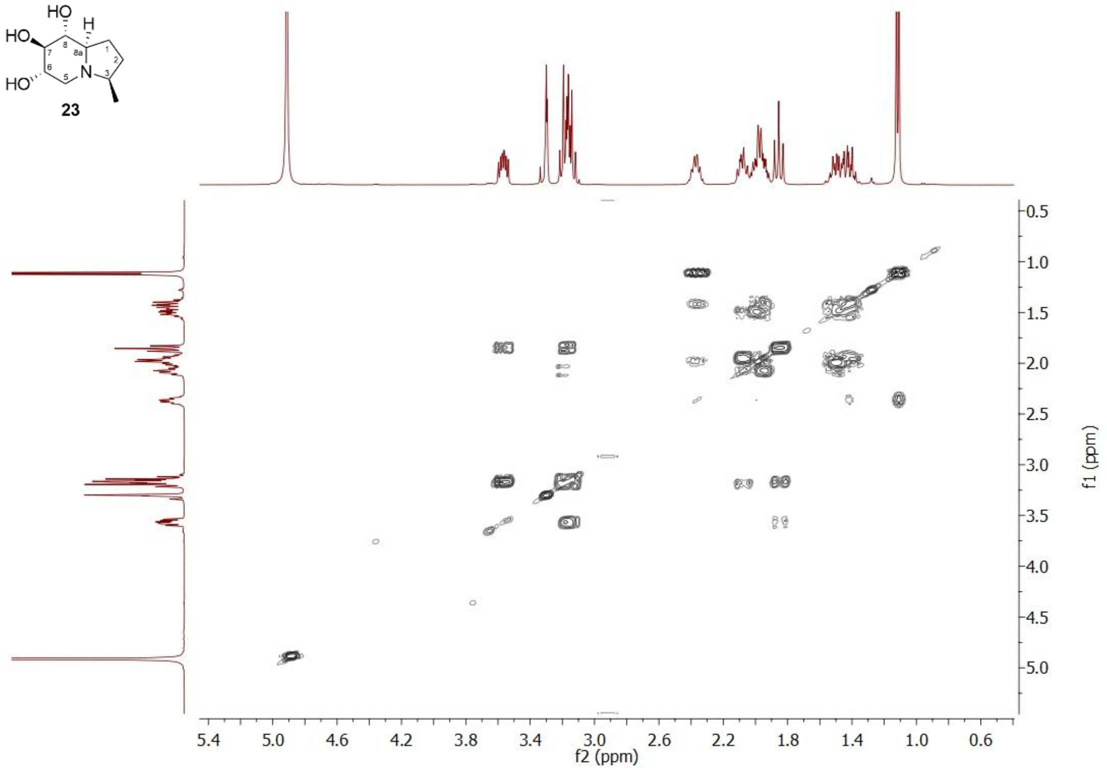

Figure S46. $^{1}H/^{1}H$ gCOSY (400 MHz, CD_3OD) of compound **23**.

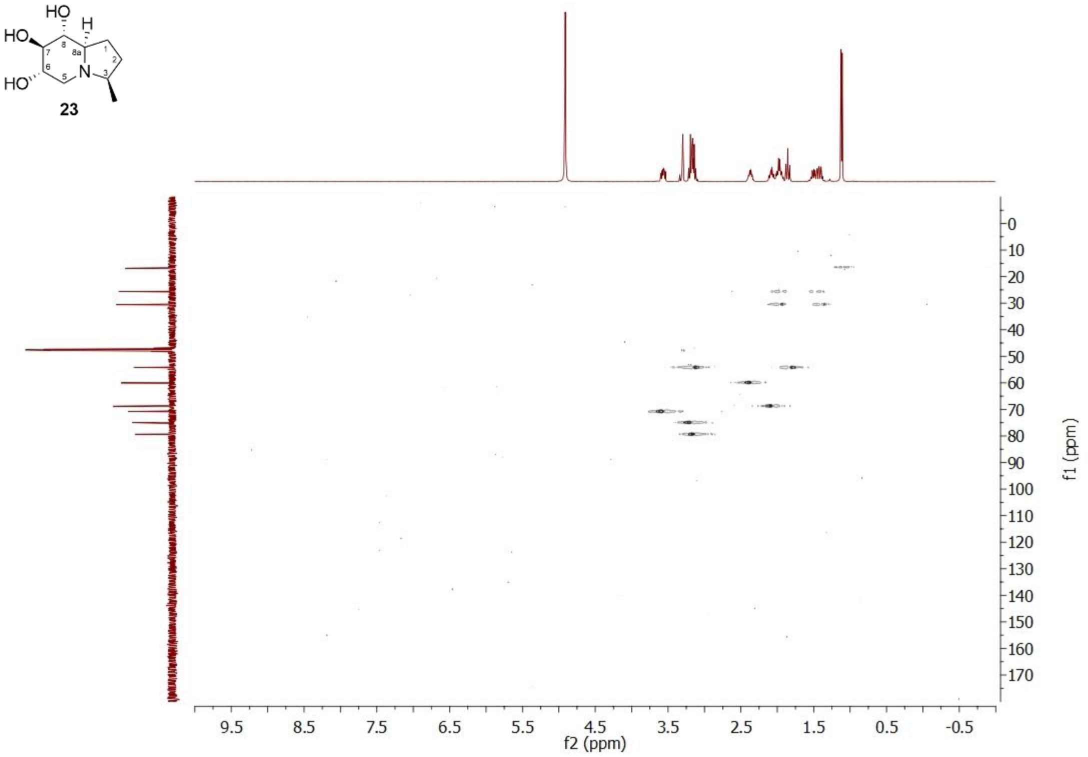

Figure S47. $^{1}H/^{13}C$ gHSQC (400/100 MHz, CD_3OD) of compound **23**.

3. Crystal structure determination for compounds 13 and 14

Single crystals of both compounds were mounted in a loop and coated with a trace of silicone oil.

Data collection were performed at 100° K with a Bruker Apex-II CCD diffractometer, using a Cu–Kα (λ = 1.54184 Å) radiation.

Data were collected, reflections were indexed and processed, and the files scaled and corrected for absorption, using Bruker APEX2, SAINT and SADABS-2016/2[3] routine.

The integrated intensities, measured using the φ and ω scan mode, were corrected for Lorentz and polarization effects.

Structures were solved by direct methods of SIR2019,[4] the refinement was performed using the full-matrix least squares on F^2 provided, within WinGX v.2013.3 routine,[5] by SHELXL2018.[6]

Non-hydrogen atoms were refined anisotropically whereas hydrogen atoms were refined as isotropic.

Data can be obtained free of charge from the Cambridge Crystallographic Data Centre via www.ccdc.cam.ac.uk/data_request/cif, with the deposition numbers indicated for each compound.

Crystallographic data for compound 13:

2x($C_{13}H_{23}NO_4$), Formula Weight =257.32, Orthorhombic, space group P 21 21 21

a=6.6305(1), b=19.9036(4), c=21.4870(5)Å, V=2835.7(1)$Å^3$

Z=4 D_c=1.205, μ=0.726mm^{-1}, F (000) = 1120.

19969 reflections were collected with a 3.026<θ< 68.244 range with a completeness to theta 99.1%; 5114 were unique, the parameters were 325 and the final R index was 0.0401 for reflections having I>2σI.

A translucent, needle shaped crystal (0.200x0.100x0.050), was used for data collection.

Hydrogen atoms were all assigned in calculated positions as riding atoms.

All bond lengths are in normal ranges.

In molecular packing two relevant hydrogen bonds were observed with the following parameters, both intramolecular:

**Donor--H**	**Donor...Acceptor**	**H... Acceptor**	**Donor--H..... Acceptor**
O2A ---H2A	O2A …N1B (0)	H2A ...N1B (0)	O2A ---H2A ...N1B (0)
0.840 Å	2.775 Å	1.937 Å	175.29°

O2B ---H2B	O2B …N1A (0)	H2B ...N1A (0)	O2B ---H2B ...N1A (0)
0.840 Å	2.763 Å	1.923 Å	178.36°

where (0) is the equivalent position: x,y,z

CCDC 2408580 contains the supplementary crystallographic data for this structure.

Table 1. Crystal data and structure refinement for compound **13.**

Identification code	**13**
Empirical formula	2 x (C13 H23 N O4)
Formula weight	257.32
Temperature	100(2) K
Wavelength	1.54178 A
Crystal system, space group	Orthorhombic, P 21 21 21
Unit cell dimensions	a=6.6305(1) A alpha = 90 deg.
	b=19.9036(4) A beta = 90 deg.
	c=21.4870(5) A gamma = 90 deg.
Volume	2835.66(10) A^3
Z, Calculated density	4, 1.205 Mg/m^3
Absorption coefficient	0.726 mm^-1
F(000)	1120
Crystal size	0.2 x 0.1 x 0.05 mm
Theta range for data collection	3.026 to 68.244 deg.
Limiting indices	-7<=h<=7, -23<=k<=23, -25<=l<=24
Reflections collected / unique	19969 / 5114 [R(int) = 0.0679]
Completeness to theta = 67.679	99.1 %
Refinement method	Full-matrix least-squares on F^2
Data / restraints / parameters	5114 / 0 / 325
Goodness-of-fit on F^2	1.003
Final R indices [I>2sigma(I)]	R1 = 0.0401, wR2 = 0.0999
R indices (all data)	R1 = 0.0421, wR2 = 0.1020
Absolute structure parameter	-0.10(8)
Extinction coefficient	n/a
Largest diff. peak and hole	0.203 and -0.246 e.A^-3

Table 2. Atomic coordinates (x 10^4) and equivalent isotropic displacement parameters (A^2 x 10^3) for **13.** U(eq) is defined as one third of the trace of the orthogonalized Uij tensor.

	x	y	z	U(eq)
N(1A)	1938(3)	2227(1)	3660(1)	15(1)
O(1A)	3208(3)	2951(1)	2469(1)	21(1)
O(2A)	2459(3)	4065(1)	2199(1)	23(1)
O(3A)	597(3)	2028(1)	1717(1)	28(1)
O(4A)	2066(4)	2779(1)	1073(1)	36(1)
C(1A)	1188(4)	2740(1)	2624(1)	19(1)
C(2A)	3020(4)	3431(1)	1980(1)	23(1)
C(3A)	1295(4)	3171(1)	1574(1)	26(1)
C(4A)	162(4)	2667(1)	1990(1)	23(1)
C(5A)	1058(5)	2145(1)	1076(1)	31(1)

C(6A)	-859(6)	2178(2)	690(1)	44(1)
C(7A)	2507(6)	1615(1)	865(1)	42(1)
C(8A)	1217(4)	2106(1)	3018(1)	17(1)
C(9A)	-920(4)	1822(1)	3108(1)	24(1)
C(10A)	-1018(4)	1600(1)	3793(1)	28(1)
C(11A)	1139(4)	1658(1)	4026(1)	18(1)
C(12A)	1298(4)	1778(1)	4725(1)	23(1)
C(13A)	4138(4)	2272(1)	3701(1)	21(1)
N(1B)	5338(3)	4608(1)	2990(1)	17(1)
O(1B)	3216(3)	3964(1)	4102(1)	18(1)
O(2B)	147(3)	3384(1)	4108(1)	19(1)
O(3B)	2313(3)	5388(1)	4564(1)	27(1)
O(4B)	1574(3)	4569(1)	5264(1)	31(1)
C(1B)	2462(4)	4528(1)	3752(1)	17(1)
C(2B)	1557(4)	3730(1)	4462(1)	17(1)
C(3B)	573(4)	4371(1)	4710(1)	21(1)
C(4B)	1148(4)	4911(1)	4226(1)	18(1)
C(5B)	2133(5)	5259(1)	5216(1)	26(1)
C(6B)	4167(6)	5356(2)	5510(2)	45(1)
C(7B)	520(5)	5700(1)	5497(1)	35(1)
C(8B)	4154(4)	4948(1)	3482(1)	18(1)
C(9B)	3309(4)	5568(1)	3139(1)	24(1)
C(10B)	4798(4)	5696(1)	2605(1)	30(1)
C(11B)	6446(4)	5170(1)	2693(1)	21(1)
C(12B)	7528(4)	4968(1)	2099(1)	29(1)
C(13B)	6710(4)	4092(1)	3236(1)	25(1)

Table 3. Bond lengths [A] and angles [deg] for **13**.

N(1A)-C(13A)	1.464(3)
N(1A)-C(11A)	1.479(3)
N(1A)-C(8A)	1.478(3)
O(1A)-C(2A)	1.426(3)
O(1A)-C(1A)	1.442(3)
O(2A)-C(2A)	1.397(3)
O(2A)-H(2A)	0.8400
O(3A)-C(4A)	1.430(3)
O(3A)-C(5A)	1.430(3)
O(4A)-C(3A)	1.425(3)
O(4A)-C(5A)	1.428(3)
C(1A)-C(8A)	1.521(3)
C(1A)-C(4A)	1.529(3)
C(1A)-H(1A)	1.0000
C(2A)-C(3A)	1.529(4)
C(2A)-H(2A1)	1.0000
C(3A)-C(4A)	1.541(3)
C(3A)-H(3A)	1.0000
C(4A)-H(4A)	1.0000
C(5A)-C(7A)	1.498(5)
C(5A)-C(6A)	1.518(5)
C(6A)-H(6A1)	0.9800
C(6A)-H(6A2)	0.9800
C(6A)-H(6A3)	0.9800
C(7A)-H(7A1)	0.9800
C(7A)-H(7A2)	0.9800
C(7A)-H(7A3)	0.9800
C(8A)-C(9A)	1.537(3)
C(8A)-H(8A)	1.0000
C(9A)-C(10A)	1.538(3)

C(9A)-H(9A1)	0.9900
C(9A)-H(9A2)	0.9900
C(10A)-C(11A)	1.520(4)
C(10A)-H(10A)	0.9900
C(10A)-H(10B)	0.9900
C(11A)-C(12A)	1.525(3)
C(11A)-H(11A)	1.0000
C(12A)-H(12A)	0.9800
C(12A)-H(12B)	0.9800
C(12A)-H(12C)	0.9800
C(13A)-H(13A)	0.9800
C(13A)-H(13B)	0.9800
C(13A)-H(13C)	0.9800
N(1B)-C(13B)	1.469(3)
N(1B)-C(8B)	1.480(3)
N(1B)-C(11B)	1.484(3)
O(1B)-C(2B)	1.424(3)
O(1B)-C(1B)	1.441(3)
O(2B)-C(2B)	1.390(3)
O(2B)-H(2B)	0.8400
O(3B)-C(4B)	1.423(3)
O(3B)-C(5B)	1.431(3)
O(4B)-C(3B)	1.418(3)
O(4B)-C(5B)	1.426(3)
C(1B)-C(8B)	1.514(3)
C(1B)-C(4B)	1.542(3)
C(1B)-H(1B)	1.0000
C(2B)-C(3B)	1.528(3)
C(2B)-H(2B1)	1.0000
C(3B)-C(4B)	1.543(3)
C(3B)-H(3B)	1.0000
C(4B)-H(4B)	1.0000
C(5B)-C(6B)	1.501(4)
C(5B)-C(7B)	1.509(4)
C(6B)-H(6B1)	0.9800
C(6B)-H(6B2)	0.9800
C(6B)-H(6B3)	0.9800
C(7B)-H(7B1)	0.9800
C(7B)-H(7B2)	0.9800
C(7B)-H(7B3)	0.9800
C(8B)-C(9B)	1.543(3)
C(8B)-H(8B)	1.0000
C(9B)-C(10B)	1.535(4)
C(9B)-H(9B1)	0.9900
C(9B)-H(9B2)	0.9900
C(10B)-C(11B)	1.525(4)
C(10B)-H(10C)	0.9900
C(10B)-H(10D)	0.9900
C(11B)-C(12B)	1.518(4)
C(11B)-H(11B)	1.0000
C(12B)-H(12D)	0.9800
C(12B)-H(12E)	0.9800
C(12B)-H(12F)	0.9800
C(13B)-H(13D)	0.9800
C(13B)-H(13E)	0.9800
C(13B)-H(13F)	0.9800
C(13A)-N(1A)-C(11A)	111.82(18)
C(13A)-N(1A)-C(8A)	112.83(18)
C(11A)-N(1A)-C(8A)	104.81(17)
C(2A)-O(1A)-C(1A)	106.47(18)

C(2A)-O(2A)-H(2A)	109.5
C(4A)-O(3A)-C(5A)	107.05(17)
C(3A)-O(4A)-C(5A)	108.2(2)
O(1A)-C(1A)-C(8A)	111.00(19)
O(1A)-C(1A)-C(4A)	103.64(18)
C(8A)-C(1A)-C(4A)	114.99(19)
O(1A)-C(1A)-H(1A)	109.0
C(8A)-C(1A)-H(1A)	109.0
C(4A)-C(1A)-H(1A)	109.0
O(2A)-C(2A)-O(1A)	112.39(19)
O(2A)-C(2A)-C(3A)	107.4(2)
O(1A)-C(2A)-C(3A)	105.04(18)
O(2A)-C(2A)-H(2A1)	110.6
O(1A)-C(2A)-H(2A1)	110.6
C(3A)-C(2A)-H(2A1)	110.6
O(4A)-C(3A)-C(2A)	110.4(2)
O(4A)-C(3A)-C(4A)	104.87(18)
C(2A)-C(3A)-C(4A)	104.71(19)
O(4A)-C(3A)-H(3A)	112.1
C(2A)-C(3A)-H(3A)	112.1
C(4A)-C(3A)-H(3A)	112.1
O(3A)-C(4A)-C(1A)	111.2(2)
O(3A)-C(4A)-C(3A)	104.1(2)
C(1A)-C(4A)-C(3A)	103.74(19)
O(3A)-C(4A)-H(4A)	112.4
C(1A)-C(4A)-H(4A)	112.4
C(3A)-C(4A)-H(4A)	112.4
O(4A)-C(5A)-O(3A)	104.42(18)
O(4A)-C(5A)-C(7A)	108.7(3)
O(3A)-C(5A)-C(7A)	108.3(2)
O(4A)-C(5A)-C(6A)	110.6(2)
O(3A)-C(5A)-C(6A)	110.7(3)
C(7A)-C(5A)-C(6A)	113.7(2)
C(5A)-C(6A)-H(6A1)	109.5
C(5A)-C(6A)-H(6A2)	109.5
H(6A1)-C(6A)-H(6A2)	109.5
C(5A)-C(6A)-H(6A3)	109.5
H(6A1)-C(6A)-H(6A3)	109.5
H(6A2)-C(6A)-H(6A3)	109.5
C(5A)-C(7A)-H(7A1)	109.5
C(5A)-C(7A)-H(7A2)	109.5
H(7A1)-C(7A)-H(7A2)	109.5
C(5A)-C(7A)-H(7A3)	109.5
H(7A1)-C(7A)-H(7A3)	109.5
H(7A2)-C(7A)-H(7A3)	109.5
N(1A)-C(8A)-C(1A)	112.86(18)
N(1A)-C(8A)-C(9A)	103.96(18)
C(1A)-C(8A)-C(9A)	111.32(19)
N(1A)-C(8A)-H(8A)	109.5
C(1A)-C(8A)-H(8A)	109.5
C(9A)-C(8A)-H(8A)	109.5
C(8A)-C(9A)-C(10A)	105.3(2)
C(8A)-C(9A)-H(9A1)	110.7
C(10A)-C(9A)-H(9A1)	110.7
C(8A)-C(9A)-H(9A2)	110.7
C(10A)-C(9A)-H(9A2)	110.7
H(9A1)-C(9A)-H(9A2)	108.8
C(11A)-C(10A)-C(9A)	104.7(2)
C(11A)-C(10A)-H(10A)	110.8
C(9A)-C(10A)-H(10A)	110.8
C(11A)-C(10A)-H(10B)	110.8

C(9A)-C(10A)-H(10B)	110.8
H(10A)-C(10A)-H(10B)	108.9
N(1A)-C(11A)-C(10A)	102.63(18)
N(1A)-C(11A)-C(12A)	112.31(18)
C(10A)-C(11A)-C(12A)	113.7(2)
N(1A)-C(11A)-H(11A)	109.3
C(10A)-C(11A)-H(11A)	109.3
C(12A)-C(11A)-H(11A)	109.3
C(11A)-C(12A)-H(12A)	109.5
C(11A)-C(12A)-H(12B)	109.5
H(12A)-C(12A)-H(12B)	109.5
C(11A)-C(12A)-H(12C)	109.5
H(12A)-C(12A)-H(12C)	109.5
H(12B)-C(12A)-H(12C)	109.5
N(1A)-C(13A)-H(13A)	109.5
N(1A)-C(13A)-H(13B)	109.5
H(13A)-C(13A)-H(13B)	109.5
N(1A)-C(13A)-H(13C)	109.5
H(13A)-C(13A)-H(13C)	109.5
H(13B)-C(13A)-H(13C)	109.5
C(13B)-N(1B)-C(8B)	112.97(19)
C(13B)-N(1B)-C(11B)	112.04(19)
C(8B)-N(1B)-C(11B)	103.00(17)
C(2B)-O(1B)-C(1B)	105.72(17)
C(2B)-O(2B)-H(2B)	109.5
C(4B)-O(3B)-C(5B)	109.54(17)
C(3B)-O(4B)-C(5B)	109.25(18)
O(1B)-C(1B)-C(8B)	111.88(19)
O(1B)-C(1B)-C(4B)	103.65(17)
C(8B)-C(1B)-C(4B)	113.56(18)
O(1B)-C(1B)-H(1B)	109.2
C(8B)-C(1B)-H(1B)	109.2
C(4B)-C(1B)-H(1B)	109.2
O(2B)-C(2B)-O(1B)	112.59(18)
O(2B)-C(2B)-C(3B)	108.53(19)
O(1B)-C(2B)-C(3B)	104.22(17)
O(2B)-C(2B)-H(2B1)	110.4
O(1B)-C(2B)-H(2B1)	110.4
C(3B)-C(2B)-H(2B1)	110.4
O(4B)-C(3B)-C(2B)	108.9(2)
O(4B)-C(3B)-C(4B)	104.82(18)
C(2B)-C(3B)-C(4B)	104.00(18)
O(4B)-C(3B)-H(3B)	112.8
C(2B)-C(3B)-H(3B)	112.8
C(4B)-C(3B)-H(3B)	112.8
O(3B)-C(4B)-C(3B)	104.82(18)
O(3B)-C(4B)-C(1B)	111.1(2)
C(3B)-C(4B)-C(1B)	103.89(17)
O(3B)-C(4B)-H(4B)	112.2
C(3B)-C(4B)-H(4B)	112.2
C(1B)-C(4B)-H(4B)	112.2
O(4B)-C(5B)-O(3B)	105.33(18)
O(4B)-C(5B)-C(6B)	109.1(2)
O(3B)-C(5B)-C(6B)	108.3(2)
O(4B)-C(5B)-C(7B)	110.3(2)
O(3B)-C(5B)-C(7B)	110.2(2)
C(6B)-C(5B)-C(7B)	113.3(2)
C(5B)-C(6B)-H(6B1)	109.5
C(5B)-C(6B)-H(6B2)	109.5
H(6B1)-C(6B)-H(6B2)	109.5
C(5B)-C(6B)-H(6B3)	109.5

H(6B1)-C(6B)-H(6B3)	109.5
H(6B2)-C(6B)-H(6B3)	109.5
C(5B)-C(7B)-H(7B1)	109.5
C(5B)-C(7B)-H(7B2)	109.5
H(7B1)-C(7B)-H(7B2)	109.5
C(5B)-C(7B)-H(7B3)	109.5
H(7B1)-C(7B)-H(7B3)	109.5
H(7B2)-C(7B)-H(7B3)	109.5
N(1B)-C(8B)-C(1B)	114.51(18)
N(1B)-C(8B)-C(9B)	102.59(18)
C(1B)-C(8B)-C(9B)	110.8(2)
N(1B)-C(8B)-H(8B)	109.6
C(1B)-C(8B)-H(8B)	109.6
C(9B)-C(8B)-H(8B)	109.6
C(10B)-C(9B)-C(8B)	104.8(2)
C(10B)-C(9B)-H(9B1)	110.8
C(8B)-C(9B)-H(9B1)	110.8
C(10B)-C(9B)-H(9B2)	110.8
C(8B)-C(9B)-H(9B2)	110.8
H(9B1)-C(9B)-H(9B2)	108.9
C(11B)-C(10B)-C(9B)	104.8(2)
C(11B)-C(10B)-H(10C)	110.8
C(9B)-C(10B)-H(10C)	110.8
C(11B)-C(10B)-H(10D)	110.8
C(9B)-C(10B)-H(10D)	110.8
H(10C)-C(10B)-H(10D)	108.9
N(1B)-C(11B)-C(12B)	113.3(2)
N(1B)-C(11B)-C(10B)	102.49(19)
C(12B)-C(11B)-C(10B)	114.6(2)
N(1B)-C(11B)-H(11B)	108.7
C(12B)-C(11B)-H(11B)	108.7
C(10B)-C(11B)-H(11B)	108.7
C(11B)-C(12B)-H(12D)	109.5
C(11B)-C(12B)-H(12E)	109.5
H(12D)-C(12B)-H(12E)	109.5
C(11B)-C(12B)-H(12F)	109.5
H(12D)-C(12B)-H(12F)	109.5
H(12E)-C(12B)-H(12F)	109.5
N(1B)-C(13B)-H(13D)	109.5
N(1B)-C(13B)-H(13E)	109.5
H(13D)-C(13B)-H(13E)	109.5
N(1B)-C(13B)-H(13F)	109.5
H(13D)-C(13B)-H(13F)	109.5
H(13E)-C(13B)-H(13F)	109.5

Table 4. Anisotropic displacement parameters (A^2 x 10^3) for **13.**
The anisotropic displacement factor exponent takes the form:
-2 pi^2 [h^2 a*^2 U11 + ... + 2 h k a* b* U12]

	U11	U22	U33	U23	U13	U12
N(1A)	13(1)	19(1)	14(1)	1(1)	1(1)	-1(1)
O(1A)	21(1)	21(1)	20(1)	4(1)	1(1)	-3(1)
O(2A)	29(1)	18(1)	22(1)	-1(1)	-6(1)	-3(1)
O(3A)	47(1)	21(1)	16(1)	0(1)	-1(1)	-10(1)
O(4A)	64(2)	25(1)	19(1)	-3(1)	7(1)	-19(1)
C(1A)	20(1)	20(1)	17(1)	1(1)	0(1)	-1(1)

C(2A)	33(2)	18(1)	19(1)	3(1)	4(1)	-3(1)
C(3A)	39(2)	21(1)	18(1)	2(1)	-2(1)	-6(1)
C(4A)	29(1)	22(1)	19(1)	1(1)	-3(1)	-5(1)
C(5A)	54(2)	22(1)	17(1)	1(1)	0(1)	-14(1)
C(6A)	71(2)	36(1)	24(1)	1(1)	-13(2)	-13(2)
C(7A)	71(2)	30(1)	26(1)	-4(1)	10(2)	-9(1)
C(8A)	16(1)	19(1)	15(1)	-1(1)	1(1)	1(1)
C(9A)	20(1)	33(1)	18(1)	4(1)	-2(1)	-8(1)
C(10A)	21(1)	39(1)	24(1)	11(1)	-2(1)	-8(1)
C(11A)	17(1)	18(1)	20(1)	3(1)	0(1)	1(1)
C(12A)	23(1)	27(1)	19(1)	5(1)	1(1)	1(1)
C(13A)	14(1)	27(1)	20(1)	1(1)	0(1)	-1(1)
N(1B)	12(1)	19(1)	20(1)	1(1)	1(1)	-1(1)
O(1B)	16(1)	17(1)	21(1)	5(1)	-1(1)	1(1)
O(2B)	19(1)	17(1)	20(1)	-3(1)	0(1)	1(1)
O(3B)	40(1)	22(1)	19(1)	-4(1)	5(1)	-8(1)
O(4B)	56(1)	19(1)	18(1)	0(1)	-7(1)	-5(1)
C(1B)	18(1)	16(1)	17(1)	2(1)	-2(1)	2(1)
C(2B)	16(1)	18(1)	18(1)	2(1)	0(1)	0(1)
C(3B)	25(1)	19(1)	18(1)	0(1)	2(1)	-2(1)
C(4B)	19(1)	16(1)	19(1)	1(1)	2(1)	0(1)
C(5B)	42(2)	19(1)	18(1)	-1(1)	2(1)	-5(1)
C(6B)	51(2)	38(2)	46(2)	-12(1)	-18(2)	1(1)
C(7B)	53(2)	26(1)	25(1)	0(1)	9(1)	1(1)
C(8B)	17(1)	19(1)	17(1)	-1(1)	-2(1)	0(1)
C(9B)	25(1)	19(1)	27(1)	5(1)	4(1)	3(1)
C(10B)	30(2)	26(1)	33(1)	11(1)	8(1)	4(1)
C(11B)	19(1)	22(1)	22(1)	4(1)	1(1)	-5(1)
C(12B)	25(1)	35(1)	27(1)	5(1)	6(1)	-1(1)
C(13B)	18(1)	25(1)	31(1)	5(1)	4(1)	5(1)

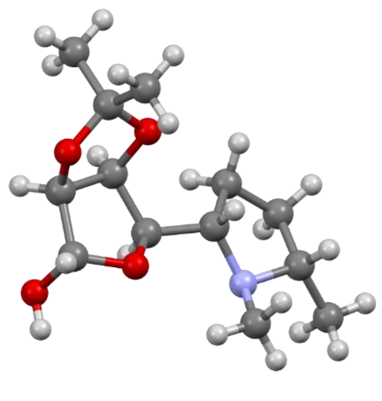

Figure S48. Ball and stick representation of the X-Ray structure of compound **13** (see main text).

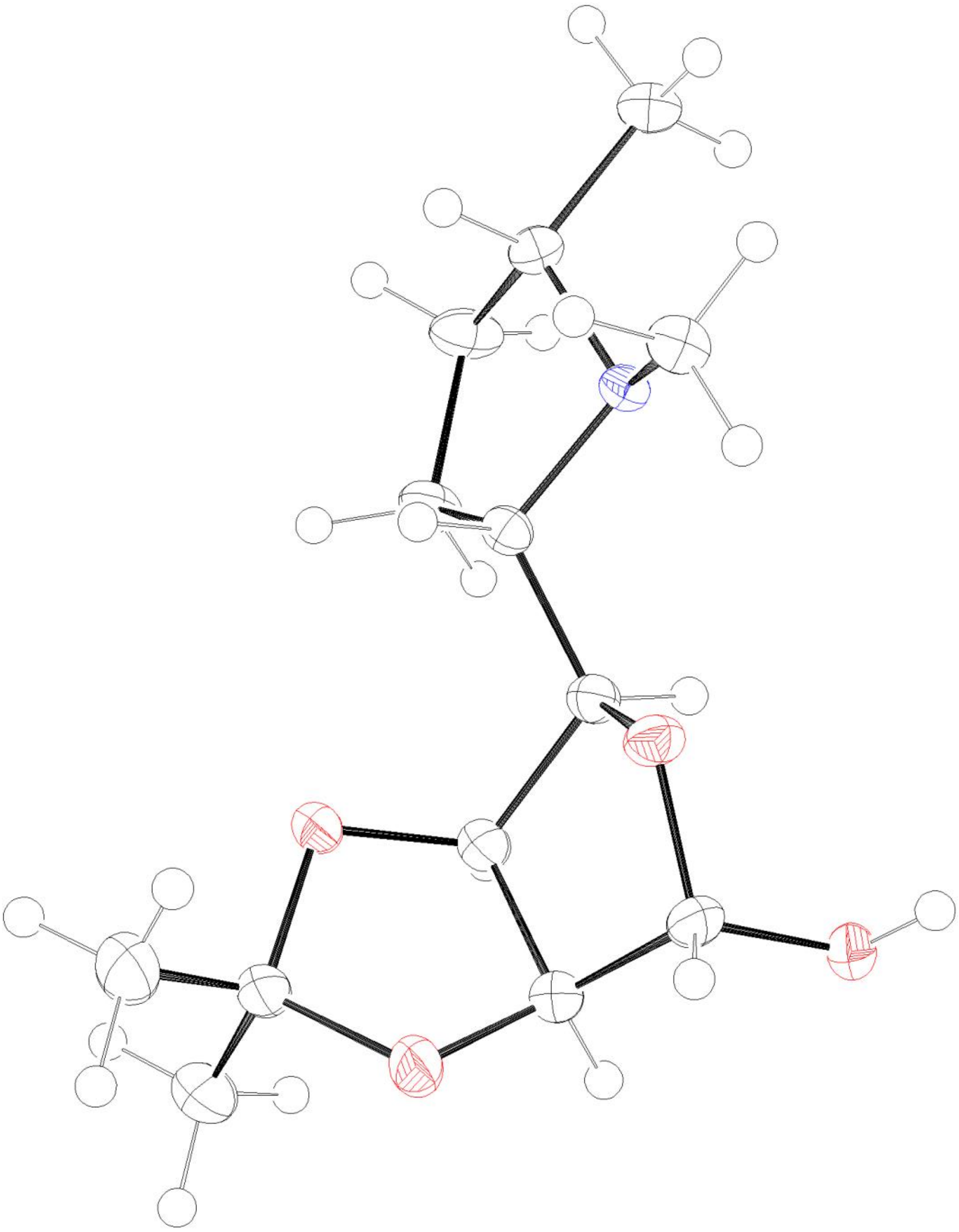

Figure S49. ORTEP drawing of **13** showing thermal ellipsoids at the 50% probability level.

Crystallographic data for compound 14:

$C_9H_{17}NO_3$, Formula Weight=187.23, Monoclinic, space group P 21

a=7.2681(3), *b*=8.6376(4), *c*=8.0771(4)Å, β=114.461(2), V=461.56(4)Å^3, Z=2 D_c=1.347, μ=0.826mm^{-1}, F(000)= 204.

5675 reflections were collected with a 6.692<θ< 70.546 range with a completeness to theta 98.1%; 1671 were unique, the parameters were 126 and the final R index was 0.0546 for reflections having I>2σI.

A colourless, plate shaped crystal (0.300x0.250x0.100) was used for data collection.

Hydrogen atoms were all assigned in calculated positions as riding atoms, except for hydrogen on O1 and O3 that has been assigned from Fourier difference map.

In fact, if assigned in calculated position, the distance between H1 (0) and H3 (1), where (1) is the equivalent position (1-x, ½+y, 1-z), would have been too short (1.38 Å).

Using FD map the distance is 1.89 Å, that is still a short but acceptable value.

All bond lengths are in normal ranges.

In molecular packing three relevant hydrogen bonds were observed with the following parameters:

**Donor--H**	**Donor...Acceptor**	**H... Acceptor**	**Donor--H..... Acceptor**
O1 ---H1	O1 …N1 (1)	H1 ...N1 (1)	O1 ---H1 ...N1 (1)
0.871 Å	2.813 Å	1.996 Å	155.60°

O2 ---H2	O2 …O3 (2)	H2 ...O3 (2)	O2 ---H2 ...O3 (2)
0.840 Å	2.837 Å	2.028 Å	161.44°

O3 ---H3	O3 …O1 (3)	H3 ...O1 (3)	O3 ---H3 ...O1 (3)
0.936 Å	2.859 Å	1.923 Å	177.56°

where the equivalent positions are:

(1) -x+1, +y+1/2, -z+1

(2) -x, +y+1/2, -z+1

(3) -x+1, +y-1/2, -z+1

CCDC 2408585 contains the supplementary crystallographic data for this structure.

Table 5. Crystal data and structure refinement for compound **14**.

Identification code	**14**
Empirical formula	C9 H17 N O3
Formula weight	187.23
Temperature	100(2) K
Wavelength	1.54178 A
Crystal system, space group	Monoclinic, P 21
Unit cell dimensions	a = 7.2681(3)A alpha = 90 deg.
	b = 8.6376(4)A beta = 114.461(2) deg.
	c = 8.0771(4)A gamma = 90 deg.
Volume	461.56(4) A^3
Z, Calculated density	2, 1.347 Mg/m^3
Absorption coefficient	0.826 mm^-1
F(000)	204
Crystal size	0.300 x 0.250 x 0.100 mm
Theta range for data collection	6.692 to 70.546 deg.
Limiting indices	-8<=h<=8, -10<=k<=10, -9<=l<=9
Reflections collected / unique	5675 / 1671 [R(int) = 0.0624]
Completeness to theta = 67.679	98.1 %
Refinement method	Full-matrix least-squares on F^2
Data / restraints / parameters	1671 / 1 / 126
Goodness-of-fit on F^2	1.032
Final R indices [I>2sigma(I)]	R1 = 0.0546, wR2 = 0.1514
R indices (all data)	R1 = 0.0551, wR2 = 0.1520
Absolute structure parameter	0.11(12)
Extinction coefficient	n/a
Largest diff. peak and hole	0.306 and -0.339 e.A^-3

Table 6. Atomic coordinates (x 10^4) and equivalent isotropic displacement parameters (A^2 x 10^3) for **14**. U(eq) is defined as one third of the trace of the orthogonalized Uij tensor.

	x	y	z	U(eq)
N(1)	3496(4)	4310(3)	2355(4)	16(1)
O(1)	6303(4)	6136(3)	6811(3)	19(1)
O(2)	2294(4)	7077(3)	5027(3)	20(1)
O(3)	1142(4)	2924(3)	4356(3)	20(1)
C(1)	4920(5)	5382(4)	3632(5)	18(1)
C(2)	4837(5)	5185(3)	5472(4)	16(1)
C(3)	2707(5)	5468(4)	5359(4)	17(1)
C(4)	1113(5)	4508(4)	3805(5)	19(1)
C(5)	1400(5)	4709(4)	2061(4)	18(1)
C(6)	87(5)	3742(5)	394(4)	23(1)
C(7)	1498(5)	3396(4)	-563(5)	22(1)
C(8)	3398(5)	4350(4)	488(4)	18(1)
C(9)	5300(5)	3737(5)	394(4)	23(1)

Table 7. Bond lengths [A] and angles [deg] for **14**.

N(1)-C(1)	1.452(4)
N(1)-C(5)	1.482(4)

N(1)-C(8)	1.480(4)
O(1)-C(2)	1.423(4)
O(1)-H(1)	0.87(8)
O(2)-C(3)	1.424(4)
O(2)-H(2)	0.8400
O(3)-C(4)	1.437(4)
O(3)-H(3)	0.94(8)
C(1)-C(2)	1.521(5)
C(1)-H(1A)	0.9900
C(1)-H(1B)	0.9900
C(2)-C(3)	1.532(5)
C(2)-H(2A)	1.0000
C(3)-C(4)	1.550(4)
C(3)-H(3A)	1.0000
C(4)-C(5)	1.517(4)
C(4)-H(4)	1.0000
C(5)-C(6)	1.535(4)
C(5)-H(5)	1.0000
C(6)-C(7)	1.548(5)
C(6)-H(6A)	0.9900
C(6)-H(6B)	0.9900
C(7)-C(8)	1.529(5)
C(7)-H(7A)	0.9900
C(7)-H(7B)	0.9900
C(8)-C(9)	1.511(5)
C(8)-H(8)	1.0000
C(9)-H(9A)	0.9800
C(9)-H(9B)	0.9800
C(9)-H(9C)	0.9800

C(1)-N(1)-C(5)	110.4(3)
C(1)-N(1)-C(8)	115.4(3)
C(5)-N(1)-C(8)	102.6(2)
C(2)-O(1)-H(1)	118(4)
C(3)-O(2)-H(2)	109.5
C(4)-O(3)-H(3)	111(5)
N(1)-C(1)-C(2)	108.5(3)
N(1)-C(1)-H(1A)	110.0
C(2)-C(1)-H(1A)	110.0
N(1)-C(1)-H(1B)	110.0
C(2)-C(1)-H(1B)	110.0
H(1A)-C(1)-H(1B)	108.4
O(1)-C(2)-C(1)	111.0(3)
O(1)-C(2)-C(3)	111.9(3)
C(1)-C(2)-C(3)	111.8(2)
O(1)-C(2)-H(2A)	107.3
C(1)-C(2)-H(2A)	107.3
C(3)-C(2)-H(2A)	107.3
O(2)-C(3)-C(2)	107.2(3)
O(2)-C(3)-C(4)	109.9(3)
C(2)-C(3)-C(4)	111.2(3)
O(2)-C(3)-H(3A)	109.5
C(2)-C(3)-H(3A)	109.5
C(4)-C(3)-H(3A)	109.5
O(3)-C(4)-C(5)	114.0(3)
O(3)-C(4)-C(3)	110.4(3)
C(5)-C(4)-C(3)	110.3(3)
O(3)-C(4)-H(4)	107.3
C(5)-C(4)-H(4)	107.3
C(3)-C(4)-H(4)	107.3
N(1)-C(5)-C(4)	110.6(3)

N(1)-C(5)-C(6)	103.8(3)
C(4)-C(5)-C(6)	118.5(3)
N(1)-C(5)-H(5)	107.8
C(4)-C(5)-H(5)	107.8
C(6)-C(5)-H(5)	107.8
C(5)-C(6)-C(7)	104.1(3)
C(5)-C(6)-H(6A)	110.9
C(7)-C(6)-H(6A)	110.9
C(5)-C(6)-H(6B)	110.9
C(7)-C(6)-H(6B)	110.9
H(6A)-C(6)-H(6B)	109.0
C(8)-C(7)-C(6)	104.6(3)
C(8)-C(7)-H(7A)	110.8
C(6)-C(7)-H(7A)	110.8
C(8)-C(7)-H(7B)	110.8
C(6)-C(7)-H(7B)	110.8
H(7A)-C(7)-H(7B)	108.9
N(1)-C(8)-C(9)	112.6(3)
N(1)-C(8)-C(7)	101.8(3)
C(9)-C(8)-C(7)	114.7(3)
N(1)-C(8)-H(8)	109.2
C(9)-C(8)-H(8)	109.2
C(7)-C(8)-H(8)	109.2
C(8)-C(9)-H(9A)	109.5
C(8)-C(9)-H(9B)	109.5
H(9A)-C(9)-H(9B)	109.5
C(8)-C(9)-H(9C)	109.5
H(9A)-C(9)-H(9C)	109.5
H(9B)-C(9)-H(9C)	109.5

Table 8. Anisotropic displacement parameters (A^2 x 10^3) for **14.**
The anisotropic displacement factor exponent takes the form:
-2 pi^2 [h^2 a*^2 U11 + ... + 2 h k a* b* U12]

	U11	U22	U33	U23	U13	U12
N(1)	16(2)	12(1)	19(1)	-1(1)	4(1)	0(1)
O(1)	19(1)	10(1)	23(1)	-3(1)	4(1)	-1(1)
O(2)	19(1)	12(1)	29(1)	-1(1)	11(1)	2(1)
O(3)	25(1)	11(1)	28(1)	0(1)	14(1)	-3(1)
C(1)	17(2)	10(2)	25(2)	-3(1)	7(1)	-2(1)
C(2)	15(2)	7(1)	20(2)	-1(1)	2(1)	-2(1)
C(3)	17(2)	10(2)	22(2)	0(1)	7(1)	1(1)
C(4)	18(2)	14(2)	22(2)	1(1)	5(1)	2(1)
C(5)	18(2)	13(2)	22(2)	1(1)	7(1)	1(1)
C(6)	18(2)	24(2)	25(2)	-3(2)	7(1)	-3(1)
C(7)	21(2)	23(2)	19(2)	-3(1)	6(1)	-2(1)
C(8)	21(2)	14(2)	16(1)	1(1)	6(1)	-1(1)
C(9)	21(2)	24(2)	25(2)	-2(2)	9(1)	4(2)

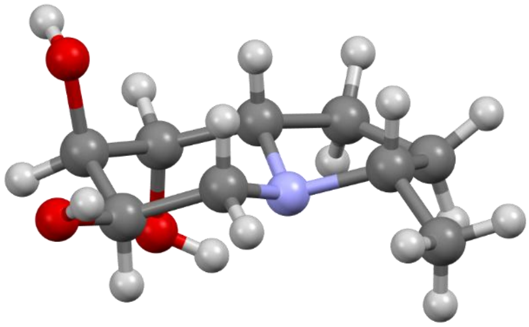

Figure S50. Ball and stick representation of the X-Ray structure of compound **14** (see main text).

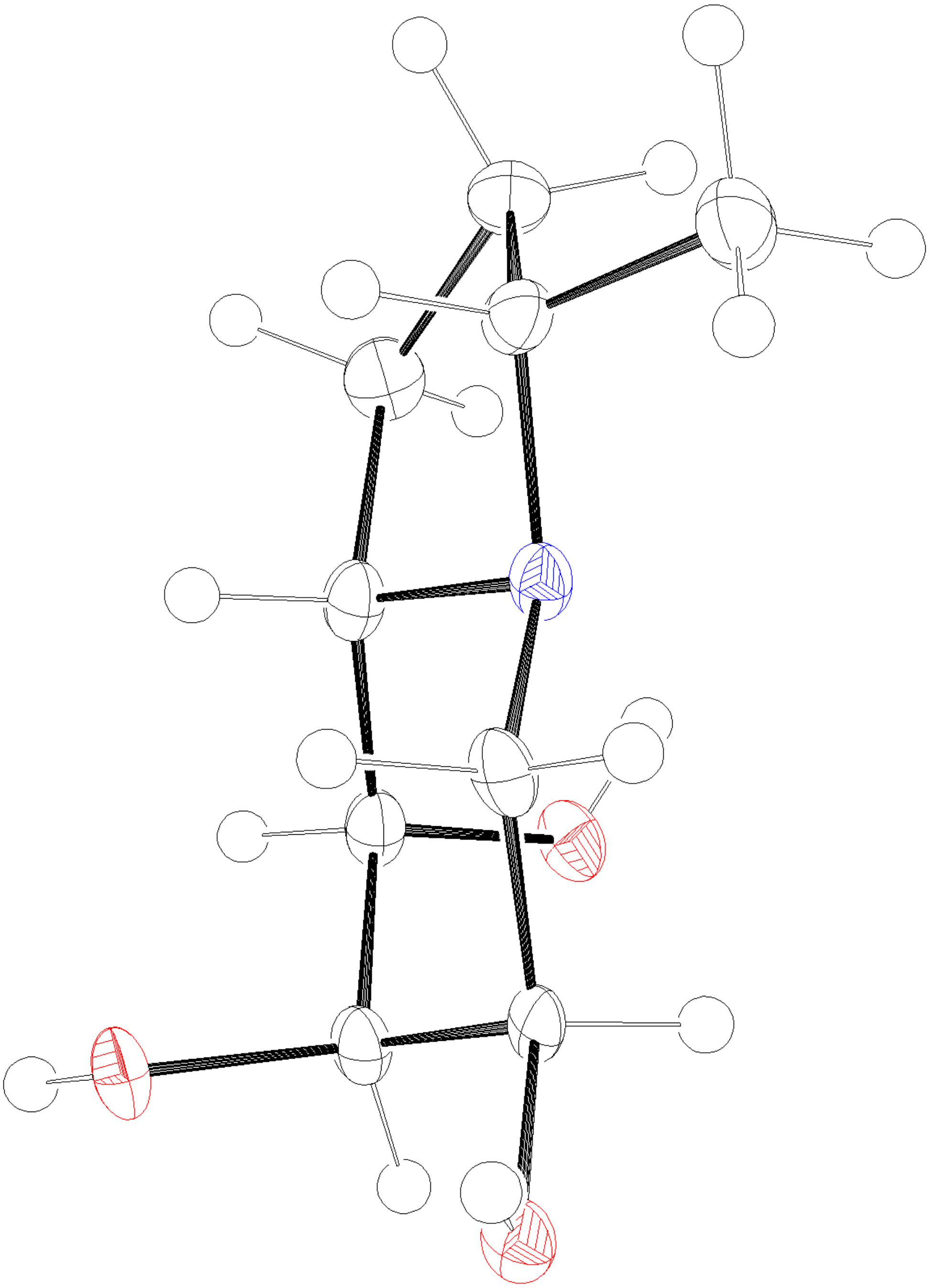

Figure S51. ORTEP drawing of **14** showing thermal ellipsoids at the 50% probability level.

4. References

[1] Mirabella, S.; Fibbi, G.; Matassini, C.; Faggi, C.; Goti, A.; Cardona, F., Accessing 2-substituted piperidine iminosugars by organometallic addition/intramolecular reductive amination: aldehyde vs. nitrone route. *Org. Biomol. Chem.* **2017**, *15*, 9121-9126.

[2] Dondoni, A.; Franco, S.; Junquera, F.; Merchán, F. L.; Merino, P.; Tejero, T. Synthesis of N-Benzyl Nitrones. *Synth. Commun.* **1994**, *24*(18), 2537–2550.

[3] Bruker: APEX2, SAINT and SADABS. Bruker AXS Inc., Madison, Wisconsin, USA, 2009.

[4] M.C. Burla, R. Caliandro, B. Carrozzini, G. L. Cascarano, C. Cuocci, C. Giacovazzo, M. Mallamo, A. Mazzone and G. Polidori "Crystal structure determination and refinement via SIR2014", *J. Appl. Cryst.* **2015**, *48*, 306–309.

[5] WinGX v.2013.3, L. J. Farrugia, *J. Appl. Cryst.* **2012**, *45*, 849-854.

[6] Sheldrick, G.M. SHELXL-2018 (2018) Program for Crystal Structure Refinement. University of Göttingen, Göttingen.
